# Supplementary material for: A modular model integrating metabolism, growth, and cell cycle predicts that fermentation is required to modulate cell size in yeast populations
Source: PLoS Comput Biol. 2025 Jul 21;21(7):e1013296. doi: 10.1371/journal.pcbi.1013296 (PMC12377586; doi:10.1371/journal.pcbi.1013296)
Supplement: S1 Text — Fig A. Comparison of the MeGro-vs-MeGro-2 building blocks. Besides being a by-product of the fermentation, ethanol may be a metabolic source for MeGro-2, and it can be exchanged with the external environment (in both directions) by means of a suitable membrane transport mechanism. External and internal ethanol, as well as the new ethanol respiration and the gluconeogenic pathway producing pyruvate from ethanol, are highlighted in bold black in MeGro-2. Fig B. Correlation between experimental and iMeGroCy-2 simulated values for the growth rate λ parameter. Values (obtained under various nutritional conditions) are reported as relative to the 2% glucose growth media, taken as reference. The green bisector line indicates the ideal, perfect correlation between the two data series. Fig C. Pairs of (K2, τ2) satisfying the constraint of Eq.(S27) for fixed values of (λ,ρ). The red markers refer to the values of τ2 coming from Table D in S1 Text; the black circle refers to the chosen setting. Fig D. Protein distributions for different low glucose and ethanol concentrations. Curves drawn for GroCy-2 model parameters inherited from glucose 2%, without any further tuning than the one provided by the MeGro-2 interconnection (green curves), with also a further (though minimal) tuning (blue curves), compared to the experimental ones (red curves). The grey shade refers to the glucose 2% experimental distribution reference. Fig E. Correlation between MeGro-2-vs-iMeGroCy-2 growth rate. Dots are growth rate λ values obtained under different fermentative conditions (F ratio between [0, 1], color-coded) in various growth media (from 0.05% to 5% glucose). Figure F. Simulated parameters in evolving and steady state populations. A, B) time course of the average protein content, for fast (glucose 2%, panel A) and slow (glucose 0.05%, panel B) growth conditions. C, D) time course of the budding index, for fast (glucose 2%, panel C) and slow (glucose 0.05%, panel D) growth conditions. The con [file pcbi.1013296.s001.docx]

***iMeGroCy-2*: A coarse-grained model of metabolism, growth and cell cycle describing the structure of exponentially growing yeast populations**

1. ***iMeGroCy-2* in a nutshell**
2. ***iMeGroCy-2 vs iMeGroCy***
3. ***The MeGro2 module***
   1. *The optimization problem associated to pure glucose environment (*$c_{{EtOH}_{ex}}=0$*)*
   2. *The optimization problem associated to pure ethanol environment (*$c_{{glc}_{ex}}=0$*)*
4. ***The GroCy-2 module***
   1. *Asymmetrical division and genealogical age heterogeneity*
5. ***Working of iMeGroCy-2 and parameter setting***
   1. *MeGro-2 parameter setting*
   2. *GroCy-2 parameter setting*
   3. *Simulations of cell populations through iMeGroCy-2*
   4. *Sensitivity analysis*
   5. *Multi-parametric sensitivity analysis*
   6. *Simulations of cell populations through Hy-iMeGroCy-2*
6. ***Simulation of mutants populations***
   1. *Mutant hxk1 hxk2*
   2. *Mutant TM6**
   3. *Mutant rsa1*
   4. *Mutants whi5^Δ^ and whi5^4E^ (iMeGroCy-2 simulations)*
   5. *Mutants whi5^Δ^ and whi5^4E^ (Hy-iMeGroCy-2 simulations)*
7. ***Additional experimental Tables and Figures***

**1 - *iMeGroCy-2* in a nutshell**

*iMeGroCy-2* is an integrated Metabolism, Growth and Cycle model that quantitatively describes the complex interrelations connecting metabolism, growth and cell cycle at the single-cell level. It consists of the interconnection of two modules: (i) a Metabolism & Growth (*MeGro-2*) module, enlisted to exploit the external nutrient concentration in order to set the growth features as the outputs of an optimization algorithm; (ii) a Growth & Cycle (*GroCy-2*) module, fed by the *MeGro-2* outputs, describing the ribosome and protein dynamics, as well as the cell cycle phases through a sequence of timers whose length is modulated by the cell size as well. *iMeGroCy-2* comes out as the extension of an analogous model concept (namely, *iMeGroCy* [1], where the Metabolism & Growth module (named *MeGro* in the old version) has been deeply modified in order to enrich the application field not only to glucose, but also to ethanol environments. The Growth & Cycle module is the same as [1] at a cellular level, but here we extend it to a population level: indeed, differently from *iMeGroCy*, this extended version is conceived to simulate also populations with a pedigree structure, thus allowing to reproduce *in silico* the experimental features of cell populations in balanced or perturbed exponential growth.

*iMeGroCy-2* is conceived to be modular and hierarchical. The growth activity combined with the other two main cellular activities of metabolism (*MeGro-2*) and cycle (*GroCy-2*) define the modular building blocks constituting the coarse-grained backbone of *iMeGroCy-2* (see Fig 2). The wiring among the modular building blocks is functional to the integrated model's correct setting and working modes: *MeGro-2* is enlisted to exploit the environmental nutrient (i.e., the external glucose or the external ethanol) and processes the ethanol yield information coming from experimental data in order to determine the exponential growth rate (*λ*) and the ribosome-over-protein ratio (*ρ*) at steady-state as outputs of an optimization algorithm that maximizes the growth rate (see Section 3 for details on *MeGro-2*). The *MeGro-2* outputs (i.e., *λ* and *ρ*) enter *GroCy-2* as inputs, allowing to set the ribosome and protein dynamic parameters of the growth module (i.e., the very own *ρ*, which is a *GroCy-2* parameter, and the protein synthesis rate constant). The growth module is an Ordinary Differential Equation (ODE) system, describing the evolution of the overall protein and ribosome content, as well as of a couple of players (the upstream cyclin Cln3 and its inhibitor Far1) that constitute the molecular trigger activating the events eventually leading to the G_1_/S transition. In particular, the growth-to-trigger link is rendered by constraining the total amount of Cln3 to the overall protein content, while the trigger-to-cycle link is rendered by the regulation of the first part of the G_1_ length (named *T_1a_* in our model) whose end is set by the time instant when free Cln3 exceeds its inhibitor Far1. Beyond *T_1a_*, the ODE system of the growth module runs for a time length spanning 3 timers: *T_1b_*, *T_2_* and *T_B_*. *T_1b_* length is regulated by the initial size of the cell, allowing larger cells to have a shorter *T_1b_* period, whilst *T_2_* and *T_B_* (the former constituting the G_1_ phase together with *T_1a_* and *T_1b_*, the latter rendering the whole S+G_2_+M-budded phase) are set without constraining them to the size of the cell. At cell division, ribosome and protein content divide asymmetrically and feed the newborn daughter cell and the old parent cell starting a new cycle (see the Section 4 for details on *GroCy-2*).

**2 - *iMeGroCy-2 vs iMeGrocy***

The model here proposed extends the *MeGro* and *GroCy* modules of [1] making the formermodulefruitful for a much wider range of *in silico* experiments, and the latter modulesuitable for populations. Straightforwardly, the novelmodules and their interconnection are here called *MeGro-2*, *GroCy-2* and *iMeGroCy-2*, respectively. *MeGro-2* is conceived to overcome drawbacks and limitations of *MeGro*, which was able to exploit only glucose as cell nutrient. Conversely, *MeGro-2* is now able to sustain the synthesis of building blocks also without extracellular glucose, by means of two new metabolic pathways fed by ethanol: (1) the ethanol respiration and (2) the gluconeogenic pathway producing pyruvate from ethanol, which in turn can feed both fermentation and respiration. So, the internal ethanol can be both a by-product of the fermentation and a metabolic source for *MeGro-2*, and it can be exchanged with the external environment (in both directions) by means of a suitable membrane transport mechanism. These modifications are of paramount importance to investigate how cells modulate their size according to different environments and/or genetic perturbations, that is the biological insight of the manuscript. A comparison of the *MeGro*-vs-*MeGro-2* building blocks is reported in Fig A to better highlight the differences between the two modules.

**Fig A. Comparison of the *MeGro*-vs-*MeGro-2* building blocks.** Besides being a by-product of the fermentation, ethanol may be a metabolic source for *MeGro-2*, and it can be exchanged with the external environment (in both directions) by means of a suitable membrane transport mechanism. External and internal ethanol, as well as the new ethanol respiration and the gluconeogenic pathway producing pyruvate from ethanol, are highlighted in bold black in *MeGro-2*.

Besides the extension of *MeGro*, in this manuscript we exploited the new *iMeGroCy-2* to build up an *in silico* environment for populations of cells, by introducing in *GroCy-2* a pedigree framework following the destiny of cell clusters originated from a single founder cell. The improvement from mere single-cells (as in [1]) to the proposed population setting is mandatory to reproduce *in silico* the experimental features of cell populations in balanced or perturbed exponential growth, such as protein distributions of growing populations obtained by flow cytometry. Thanks to the novel pedigree population structure, we investigated in populations how metabolism drives the modulation of cell size,

1. according to different nutritional environments: indeed, we managed to do it by exploiting the *MeGro-2* capability of setting the growth rate of the population (as well as its ribosome-over-protein ratio) according to the external nutrient; a coarse tuning of few selected parameters has been also performed on the basis of evidences coming from the literature;
2. according to genetic perturbations: such a task has been performed in a twofold way. From the one hand, the model has been able to predict the correct experimental behavior of a couple of mutants (defecting of the upper part of the glycolysis and of the glucose transport) by properly varying the correct parameters of the *MeGro-2* module; from the other hand, the opposite game has been played in reverse engineering: given a mutant whose lab experiments provided specific growth and cycle output variations with respect to the wild type case, we exploited the *iMeGroCy-2* model to understand what parameter variations could be responsible for it. See Section 6 for the details on mutant simulations.

Finally, in this manuscript we also show how the proposed model can be used as a scaffold for plug-ins that include molecular details not addressed in the coarse-grained texture of *iMeGroCy-2*, allowing its use to refine predictions from the cellular to the molecular level. Such a potentiality was merely mentioned in [1], whilst now we show how to put it into practice by suitably plugging-in a molecular module for the G_1_/S transition that partly replaces the *GroCy-2* machinery, setting the length of the G_1_ phase. The resulting hybrid model (*Hy-iMeGroCy-2*) quantitatively describes the temporal features and the protein distributions of a wild-type population, as well as of mutants whose genetic perturbation could be readily interpreted by the molecular module: indeed, the use of *Hy-iMeGroCy-2* to deal with mutants of the cycle (not finely detailed by *iMeGroCy-2*) allowed to predict the qualitative behavior emerging from experimental population outputs by suitably tuning specific molecular parameters.

In the main text are reported a couple of Tables aiming at summarizing the *MeGro-2*-vs-*MeGro* differences (Table 1) and the novel *iMeGroCy-2* applications (Table 2).

**3 - *The MeGro-2 module***

*MeGro-2* is the extension of the basic *MeGro* module presented in [1], which was derived from the "self-replicator" model proposed in [2]. Both *MeGro* and *MeGro-2* are conceived to highlight the common patterns connecting growth rate-dependent regulation of cell size, ribosomal content, and metabolic efficiency in yeast *Saccharomyces cerevisiae*, showing how such patterns arise from the growth rate maximization. While *MeGro* can only use glucose as a nutrient, *MeGro-2* allows the metabolic engine to consume both external glucose and ethanol.

*MeGro-2* is a steady-state model where the concentrations of enzymes and metabolites involved in the metabolic conversions are constrained by kinetic laws (linking fluxes to the concentrations of enzymes and metabolites) and algebraic constraints coming from the balance condition (i.e., the exponential growth) of an ODE system describing the dynamics of enzymes and metabolites.

*MeGro-2* accounts for five classes of protein-like players having enzymatic activity and seven kinds of metabolites. The five players with enzymatic activity (square blocks in the *MeGro-2* scheme of Fig 2) are (i) the hexose transporters, *'hxt*,' (ii) the glycolytic enzymes, *'gly*,' (iii) the ribosomes, *'rib*,' the enzymes of (iv) respiration and (v) fermentation pathways, *‘resp’* and *‘ferm’* respectively. Five kinds of metabolites are involved in metabolic conversions (ovals in the *MeGro-2* scheme of Fig 2), namely the (a) extracellular and (b) intracellular glucose, *‘glc_ex_*,' and *‘glc_in_’* respectively, (c) extracellular and (d) intracellular ethanol, *EtOH_ex_* and *EtOH_in_,*respectively, and (e) pyruvate, *‘pyr’*; other two kinds of metabolites are involved in energy production/consumption: (f) ATP and (g) ADP. In the following, we will indicate by $c_{x}$*, x ∈ Prot υ Met,* with *Prot =* {*hxt, gly, rib, resp, ferm*}and *Met =* {*glc_ex_, glc_in_, EtOH_ex_, EtOH_in_, pyr, ATP, ADP*}, the protein/metabolite player concentrations, (mM). The notation is summarized in Table A.

**Table A. *MeGro-2* molecular players**

| **Protein-like players concentrations** | |  | **Metabolites concentrations** | |
| --- | --- | --- | --- | --- |
| $c_{hxt}$ | Hexose transporter |  | $c_{{glc}_{ex}}$ | Extracellular glucose |
| $c_{gly}$ | Glycolytic enzymes |  | $c_{{glc}_{in}}$ | Intracellular glucose |
| $c_{rib}$ | Ribosomes |  | $c_{{EtOH}_{ex}}$ | Extracellular ethanol |
| $c_{resp}$ | Enzymes of respiration pathway |  | $c_{{EtOH}_{in}}$ | Intracellular ethanol |
| $c_{ferm}$ | Enzymes of fermentation pathway |  | $c_{pyr}$ | Pyruvate |
|  | |  | $c_{ADP}$ | ADP |
|  |  |  | $c_{ATP}$ | ATP |

Fluxes involved in *MeGro-2* come out from the following formal chemical reaction scheme.

Indeed, these fluxesaccount for glucose transport from the extracellular compartment inside the cell (*ν_hxt_*), glycolysis (*ν_gly_*), respiration and fermentation from pyruvate (*ν_resp_* and *ν_ferm_*, respectively), biomass production (*ν_rib_*), ethanol transport from the extracellular compartment inside the cell and vice versa (*ν_EtOHtr_*), gluconeogenesis (*ν_gly2_*) and respiration from ethanol (*ν_resp2_*). They will be denoted by *ν_x_* (mM/h), *x ∈ Prot2*, with *Prot2* = {*hxt, gly, gly2, rib, resp, resp2, ferm*} and are reported in Table B.

**Table B. *MeGro-2* fluxes**

| **Fluxes** | |
| --- | --- |
| $v_{hxt}$ | Glucose transport |
| $v_{gly}$ | Glycolysis |
| $v_{resp}$ | Respiration from pyruvate |
| $v_{ferm}$ | Fermentation |
| $v_{rib}$ | Biomass production |
| $v_{{EtOH}_{tr}}$ | Ethanol transport |
| $v_{gly2}$ | Gluconeogenesis |
| $v_{resp2}$ | Respiration from ethanol |

More in details, by looking at the *MeGro-2* scheme depicted in Fig 2, but also at its equations reported in the following, we note that the model accounts for two respiratory pathways catalyzed by the same class of proteins, $c_{resp}$ (represented by the square “resp” in Fig 2), but fed by two different metabolites, i.e. pyruvate, $c_{pyr}$, and ethanol,$c_{{EtOH}_{in}}$. The mentioned respiratory pathways produce two distinct fluxes, i.e. *ν_resp_* and *ν_resp2_*. Moreover, two opposite pathways concerning glucose metabolism are taken into account by the present model formulation: (i) the glycolysis, converting glucose into pyruvate, represented by the flux *ν_gly_*; (ii) the gluconeogenesis, producing glucose from ethanol, modelled by the flux *ν_gly2_*. The same class of glycolytic enzymes, $c_{gly}$ (represented in Fig 2 by the "gly" square), catalyzes both fluxes. Including the gluconeogenesis pathway in the present model formulation allows *MeGro-2* to sustain the synthesis of the building blocks of the cell by two distinct pathways,one fed by the external glucose and the other one by the external ethanol.

As previously mentioned, *MeGro-2* solves an optimization algorithm aiming at maximizing the growth rate *λ* (h^-1^), subject to a pair of model inputs (namely, the external glucose $c_{{glc}_{ex}}$ and external ethanol $c_{{EtOH}_{ex}}$, considered constant by assuming a growth medium with an infinite volume) and to a set of constraints that are found out below. First constraints come out from the exponential growth conditions and link the protein-like concentrations with the total ribosomal flux *ν_rib_* (see [1] and [2] for more details):

$\lambda c_{hxt}- \alpha_{hxt} \upsilon_{rib}=0$,

$\lambda c_{gly}- \alpha_{gly} \upsilon_{rib}=0$,

$\lambda c_{rib}- \alpha_{rib} \upsilon_{rib}=0$, (S1)

$\lambda c_{resp}- \alpha_{resp} \upsilon_{rib}=0$,

$\lambda c_{ferm}- \alpha_{ferm} \upsilon_{rib}=0$,

where the protein synthesis is modeled as a fraction *α_x_* of *ν_rib_* with

$\sum_{x \epsilon Prot} \alpha_{x}=1$ with $\alpha_{x}\geq0$. (S2)

Conversely, the dynamics of the internal metabolites $c_{{glc}_{in}}$, $c_{{EtOH}_{in}}$, $c_{pyr}$, and $c_{ATP}$ are determined by the combination of the fluxes of synthesis and degradation, written according to the stoichiometry coming from the chemical reaction scheme:

$\frac{dc_{{glc}_{in}}}{dt}= \upsilon_{hxt}-\upsilon_{gly}+ \upsilon_{gly2}$, $\frac{dc_{pyr}}{dt}= 2\upsilon_{gly}- \upsilon_{ferm}- \upsilon_{resp}-600\upsilon_{rib}$ (S3)

$\frac{dc_{ATP}}{dt}= 2\upsilon_{gly}+ 10\upsilon_{resp}+5\upsilon_{resp2}-2000\upsilon_{rib}- 8\upsilon_{gly2}$,

$\frac{dc_{{EtOH}_{in}}}{dt}= \upsilon_{ferm}- 2\upsilon_{gly2}- \upsilon_{resp2}-\upsilon_{{EtOH}_{tr}}$,

so that, at steady-state, the following constraints on the metabolic fluxes can be written:

$\upsilon_{hxt}-\upsilon_{gly}+ \upsilon_{gly2}=0$,

$2\upsilon_{gly}- \upsilon_{ferm}- \upsilon_{resp}-600\upsilon_{rib}=0$, (S4)

$2\upsilon_{gly}+ 10\upsilon_{resp}+5\upsilon_{resp2}-2000\upsilon_{rib}- 8\upsilon_{gly2}=0$,

$\upsilon_{ferm}- 2\upsilon_{gly2}- \upsilon_{resp2}-\upsilon_{{EtOH}_{tr}}=0$.

The total amount of ADP and ATP is constant according to the following relationship

$c_{ADP}+c_{ATP}=1$. (S5)

All protein/metabolite concentrations $c_{x}$ are such that $c_{x}$≥ 0.

Concerning the kinetic laws of the metabolic fluxes, they are modelled using the Michaelis-Menten formalism:

$\upsilon_{hxt}=k_{cat,hxt} \frac{\frac{c_{{glc}_{ex}}}{K_{M,hxt}}}{\left( \frac{c_{{glc}_{ex}}}{K_{M,hxt}} + 1 \right)\left( \frac{c_{{glc}_{in}}}{K_{I,glc}} + 1 \right)} c_{hxt}= g_{hxt}(c_{Met2};c_{{glc}_{ex}})c_{hxt}$,

$\upsilon_{gly}=k_{cat,gly} \frac{\frac{c_{ADP}}{K_{M,glyADP}} \frac{c_{{glc}_{in}}}{K_{M,gly}}}{\left( \frac{c_{ADP}}{K_{M,glyADP}} + 1 \right)\left( \frac{c_{{glc}_{in}}}{K_{M,gly}} + 1 \right)\left( \frac{c_{pyr}}{K_{I,pyr}} + 1 \right)} c_{gly}= g_{gly}(c_{Met2})c_{gly}$,

$\upsilon_{resp}=k_{cat,resp} \frac{\frac{c_{ADP}}{K_{M,respADP}} \frac{c_{pyr}}{K_{M,resp}}}{\left( \frac{c_{ADP}}{K_{M,respADP}} + 1 \right)\left( \frac{c_{pyr}}{K_{M,resp}} + 1 \right)} c_{resp}= g_{resp}(c_{Met2})c_{resp}$,$\upsilon_{ferm}=k_{cat,ferm} \frac{\frac{c_{pyr}}{K_{M,ferm}}}{\left( \frac{c_{pyr}}{K_{M,ferm}}+ 1 \right)} c_{ferm}= g_{ferm}(c_{Met2})c_{ferm}$, (S6)

$\upsilon_{rib}=k_{cat,rib} \frac{\frac{c_{ATP}}{K_{M,ribATP}} \frac{c_{pyr}}{K_{M,rib}}}{\left( \frac{c_{ATP}}{K_{M,ribATP}} + 1 \right)\left( \frac{c_{pyr}}{K_{M,rib}} + 1 \right)} c_{rib}= g_{rib}(c_{Met2})c_{rib}$,$\upsilon_{{EtOH}_{tr}}=k_{cat,{EtOH}_{tr}} \frac{\frac{c_{{EtOH}_{in}}- c_{{EtOH}_{ex}}}{K_{M,{EtOH}_{tr}}}}{\left( \frac{c_{{EtOH}_{in}}}{K_{M,{EtOH}_{tr}}} + 1 \right)\left( \frac{c_{{EtOH}_{ex}}}{K_{M,{EtOH}_{tr}}} + 1 \right)}=g_{{EtOH}_{tr}}(c_{Met2};c_{{EtOH}_{ex}})$,$\upsilon_{gly2}=k_{cat,gly2} \frac{\frac{c_{ATP}}{K_{M,gly2ATP}} \frac{c_{{EtOH}_{in}}}{K_{M,gly2}}}{\left( \frac{c_{ATP}}{K_{M,gly2ATP}} + 1 \right)\left( \frac{c_{{EtOH}_{in}}}{K_{M,gly2}} + 1 \right)\left( \frac{c_{{glc}_{in}}}{K_{I2,glc}} + 1 \right)} c_{gly}= g_{gly2}\left( c_{Met2} \right)c_{gly},$ $\upsilon_{resp2}=k_{cat,resp2} \frac{\frac{c_{ADP}}{K_{M,resp2ADP}} \frac{c_{{EtOH}_{in}}}{K_{M,resp2}}}{\left( \frac{c_{ADP}}{K_{M,resp2ADP}} + 1 \right)\left( \frac{c_{{EtOH}_{in}}}{K_{M,resp2}} + 1 \right)} c_{resp}= g_{resp2}(c_{Met2})c_{resp}$,

where $c_{Met2}$ is the vector of the five internal metabolite concentrations *Met2* = {*glc_in_, EtOH_in_, pyr, ATP, ADP*}, which are unknown variables of the optimization algorithm. Conversely, the external metabolite concentrations $c_{{glc}_{ex}}$ and $c_{{EtOH}_{ex}}$ are fixed, so that they can be considered as constant parameters of the functions $g_{hxt}(c_{Met2};c_{{glc}_{ex}})$ and, respectively, $g_{{EtOH}_{tr}}(c_{Met2};c_{{EtOH}_{ex}})$. Equations from (S1) to (S6) define the set of algebraic differential equations of *MeGro-2*. The exponential growth rate *λ* is maximized as a function of the external glucose concentration $c_{{glc}_{ex}}$, or of the external ethanol concentration, $c_{{EtOH}_{ex}}$, (or even a combination of both inputs) with the fractions *α_x_* as optimization variables, and subject to exponential growth constraints, equations (S1), flux balance constraints, equations (S4), Michaelis-Menten flux equations (S6), and feasible constraints, equations (S2), (S5).

The optimal value of *λ* and the fractions *α_x_*, the protein/metabolite concentrations, and the protein fluxes provide a first level of *MeGro-2* outputs. Additional outcomes, computed by properly exploiting concentrations and fluxes, are:

(i) the *fermentative ratio F*,

$F=\frac{\upsilon_{ferm}}{\left( \upsilon_{ferm} + \upsilon_{resp} + \upsilon_{resp2} \right)}$; (S7)

(ii) the *ribosome-over-protein ratio ρ,* given by the following expression

$\rho=\frac{c_{rib}}{\left( c_{hxt}+c_{gly}+c_{resp}+c_{ferm} \right)*N_{aa/prot} + c_{rib}*N_{prot/rib}*N_{aa/prot\_rib}},$ (S8)

where $N_{prot/rib}$ is the average number of ribosomal proteins per ribosome, $N_{aa/prot\_rib}$ is the average number of amino acids per ribosomal protein, and $N_{aa/prot}$ is the average number of amino acids per generic protein; so the expression (S8) returns the *ribosome-over-protein ratio* in terms of number of ribosomes over the total amount of polymerized amino acids within the five macro-classes of synthesized players. Giving the total amount of proteins expressed as the total number of polymerized amino acids allows *MeGro-2* to straightforwardly communicate with *GroCy-2*, which also accounts for cell size in terms of polymerized amino acids;

(iii) the yield of ethanol *Y_EtOH/glc_* produced in the presence of glucose nutrient:

$Y_{EtOH/glc}=\frac{\upsilon_{EtOHtr}}{\upsilon_{hxt}}.$ (S9)

(iv) the protein investments

$\gamma_{j}=\frac{c_{j}}{c_{hxt}+c_{gly}+c_{resp}+c_{ferm}+ c_{rib}*N_{prot/rib}}, j\in\left\{ hxt, gly, resp, ferm \right\}$, and

$\gamma_{rib}=\frac{c_{rib}*N_{prot/rib}}{c_{hxt}+c_{gly}+c_{resp}+c_{ferm}+ c_{rib}*N_{prot/rib}},$ (S10)

which provides the percentage of proteins the cell produces within each class of players (see Fig 5).

**3.a - The optimization problem associated to pure glucose environment (**$\boldsymbol{c}_{\boldsymbol{EtOH}_{\boldsymbol{ex}}}\boldsymbol{=0}$**)**

As far as the numerical implementation of the optimization problem is concerned, disregarding the equations of the additional model outputs (S8-S10), the problem counts (i) 25 unknown variables, i.e. the 5 concentrations of the protein players, 5 metabolite concentrations (considering only the unknown metabolites, since $c_{{glc}_{ex}}$, $c_{{EtOH}_{ex}}$ are fixed based on the external environment), the 8 fluxes, the 5 fractions of ribosome flux $\alpha_{j}$, *j* *∈* *Prot*, the growth rate $\lambda$ and the fermentative ratio *F*; (ii) 20 algebraic constraints, i.e. Eqs.(S1-S2) and (S4-S6), including also the equation of *F* (S7). So, from the theoretical point of view, the optimization problem has 5 degrees of freedom and is formally set as:

$\begin{matrix} \max\\ ,{}_{Prot},c_{Prot},c_{Met2},v_{Prot2},F \end{matrix}$ such that (S1), (S2), (S4), (S5), (S6), (S7)

In the following, we propose a way to simplify the optimization problem: by suitably exploiting the model constraints we reduce the initial problem to a simpler equivalent one, where the optimization is performed w.r.t. the 6 independent unknowns $F, \upsilon_{rib},c_{{glc}_{in}}, c_{pyr},c_{{EtOH}_{in}}$ and $c_{ATP}$ and only one algebraic constraint. Indeed, from Eqs.(S1-S2) we obtain the explicit expression of the growth rate

$\lambda=\frac{\upsilon_{rib}}{c_{hxt} + c_{gly} +c_{resp} + c_{ferm} + c_{rib}}$, (S11)

as well as the equations $\alpha_{j}=c_{j}/\left( c_{hxt} + c_{gly} +c_{resp} + c_{ferm} + c_{rib} \right)$, $j \in Prot$, allowing us to derive $\alpha_{j}$ from the knowledge of the concentrations $c_{j}$. Therefore, the optimization algorithm simplifies into:

$\begin{matrix} max \\ c_{Prot},c_{Met2},v_{Prot2},F \end{matrix} \frac{\upsilon_{rib}}{c_{hxt} + c_{gly} +c_{resp} + c_{ferm} + c_{rib}}$ such that (S4), (S5), (S6), (S7)

Combining the kinetic equations of $\upsilon_{gly}$, $\upsilon_{gly2}$ and $\upsilon_{resp}$, $\upsilon_{resp2}$ in (S6), we get the relations

$\upsilon_{gly2}= \frac{g_{gly2}\left( c_{Met2} \right)}{g_{gly}\left( c_{Met2} \right)}v_{gly}=\eta\left( c_{Met2} \right) v_{gly}$,

$\upsilon_{resp2}= \frac{g_{resp2}\left( c_{Met2} \right)}{g_{resp}\left( c_{Met2} \right)}v_{resp}=\xi\left( c_{Met2} \right)v_{resp}$,

where the two ratios$\eta\left( c_{Met2} \right)$, $\xi\left( c_{Met2} \right)$ are explicit functions of the internal metabolite concentrations only. These two new kinetic equations can be substituted to the last two equations of system (S6), so rewriting it as

$\upsilon_{hxt}= g_{hxt}(c_{Met2};c_{{glc}_{ex}})c_{hxt}$,

$\upsilon_{gly}= g_{gly}(c_{Met2})c_{gly}$,

$\upsilon_{resp}= g_{resp}(c_{Met2})c_{resp}$,

$\upsilon_{ferm}= g_{ferm}(c_{Met2})c_{ferm}$, (S12)

$\upsilon_{rib}= g_{rib}(c_{Met2})c_{rib}$,

$\upsilon_{{EtOH}_{tr}}=g_{{EtOH}_{tr}}(c_{Met2};c_{{EtOH}_{ex}})$,

$\upsilon_{gly2}= \eta\left( c_{Met2} \right) v_{gly}$,

$\upsilon_{resp2}=\xi\left( c_{Met2} \right)v_{resp}$.

Exploiting the last two constraints in (S12), the flux balance constraints (S4) and the fermentative ratio definition (S7) we can express all the fluxes in terms of *F*, the ribosomal flux $\nu_{rib}$ and the metabolite concentrations as follows:

$\upsilon_{hxt}={10}^{3}\frac{\left( 1-\eta\left( c_{Met2} \right) \right)\left[ F\left( 1+\xi\left( c_{Met2} \right) \right) + \left( 1-F \right)\left( 4+1.5\xi\left( c_{Met2} \right) \right) \right]}{F\left( 1+\xi\left( c_{Met2} \right) \right)\left( 1-4\eta\left( c_{Met2} \right) \right) + \left( 1-F \right)\left( 11+5\xi\left( c_{Met2} \right)-4\eta\left( c_{Met2} \right) \right)}v_{rib}= \psi_{hxt}\left( F, c_{Met2} \right) v_{rib}$,

$\upsilon_{gly}={10}^{3}\frac{F\left( 1+\xi\left( c_{Met2} \right) \right) + \left( 1-F \right)\left( 4+1.5\xi\left( c_{Met2} \right) \right)}{F\left( 1+\xi\left( c_{Met2} \right) \right)\left( 1-4\eta\left( c_{Met2} \right) \right) + \left( 1-F \right)\left( 11+5\xi\left( c_{Met2} \right)-4\eta\left( c_{Met2} \right) \right)}v_{rib}= \psi_{gly}\left( F, c_{Met2} \right) v_{rib}$,

$\upsilon_{resp}={10}^{3}\frac{\left( 1-F \right)\left( 1.4+2.4\eta\left( c_{Met2} \right) \right)}{F\left( 1+\xi\left( c_{Met2} \right) \right)\left( 1-4\eta\left( c_{Met2} \right) \right) + \left( 1-F \right)\left( 11+5\xi\left( c_{Met2} \right)-4\eta\left( c_{Met2} \right) \right)}v_{rib}=\psi_{resp}\left( F, c_{Met2} \right) v_{rib}$,

$\upsilon_{ferm}={10}^{3}\frac{F\left( 1+\xi\left( c_{Met2} \right) \right)\left( 1.4+2.4\eta\left( c_{Met2} \right) \right)}{F\left( 1+\xi\left( c_{Met2} \right) \right)\left( 1-4\eta\left( c_{Met2} \right) \right) + \left( 1-F \right)\left( 11+5\xi\left( c_{Met2} \right)-4\eta\left( c_{Met2} \right) \right)}v_{rib}= \psi_{ferm}\left( F, c_{Met2} \right) v_{rib}$,

$\nu_{EtOHtr}={10}^{3}\frac{F\left( 1+\xi\left( c_{Met2} \right) \right)\left( 1.4+0.4\eta\left( c_{Met2} \right) \right) - \left( 1-F \right)\left( 1.4\xi\left( c_{Met2} \right)+8\eta\left( c_{Met2} \right)+5.4\xi\left( c_{Met2} \right)\eta\left( c_{Met2} \right) \right)}{F\left( 1+\xi\left( c_{Met2} \right) \right)\left( 1-4\eta\left( c_{Met2} \right) \right) + \left( 1-F \right)\left( 11+5\xi\left( c_{Met2} \right)-4\eta\left( c_{Met2} \right) \right)} v_{rib}$ $=\psi_{EtOHtr}\left( F, c_{Met2} \right) v_{rib},$ (S13)

$\upsilon_{gly2}={10}^{3}\eta\left( c_{Met2} \right)\frac{F\left( 1+\xi\left( c_{Met2} \right) \right) + \left( 1-F \right)\left( 4+1.5\xi\left( c_{Met2} \right) \right)}{F\left( 1+\xi\left( c_{Met2} \right) \right)\left( 1-4\eta\left( c_{Met2} \right) \right) + \left( 1-F \right)\left( 11+5\xi\left( c_{Met2} \right)-4\eta\left( c_{Met2} \right) \right)}v_{rib}$,

$\upsilon_{resp2}={10}^{3}\xi\left( c_{Met2} \right)\frac{\left( 1-F \right)\left( 1.4+2.4\eta\left( c_{Met2} \right) \right)}{F\left( 1+\xi\left( c_{Met2} \right) \right)\left( 1-4\eta\left( c_{Met2} \right) \right) + \left( 1-F \right)\left( 11+5\xi\left( c_{Met2} \right)-4\eta\left( c_{Met2} \right) \right)}v_{rib}$.

Now, exploiting the first six equations of (S12) (inverting the first five ones w.r.t. $c_{j}$) and taking into account the first five equations of (S13), we get the following explicit expressions for the concentrations of the protein-like players:

$c_{j}=\frac{\psi_{j}\left( F, c_{Met2} \right)}{g_{j}\left( c_{Met2} \right)}v_{rib}=f_{j}\left( F, c_{Met2} \right) v_{rib}$, $j \in\left\{ gly, resp, ferm,rib \right\}$, (S14)

$c_{hxt}=\frac{\psi_{hxt}\left( F, c_{Met2} \right)}{g_{hxt}\left( c_{Met2};c_{{glc}_{ex}} \right)}v_{rib}=f_{hxt}\left( F, c_{Met2};c_{{glc}_{ex}} \right) v_{rib}$,

where $\psi_{rib}$ is formally set equal to 1, and we get also the algebraic constraint

$g_{{EtOH}_{tr}}\left( c_{Met2};c_{{EtOH}_{ex}} \right)= g_{{EtOH}_{tr}}\left( c_{{EtOH}_{in}};c_{{EtOH}_{ex}} \right)=\psi_{EtOHtr}\left( F, c_{Met2} \right) v_{rib}$. (S15)

From Eqs. (S14) and (S11), we get

$\lambda=\frac{1}{f_{hxt}\left( F, c_{Met2};c_{{glc}_{ex}} \right)+\sum_{j \in\left\{ gly, resp, ferm,rib \right\}} f_{j}\left( F, c_{Met2} \right)}$, (S16)

where the explicit dependence on $v_{rib}$ disappears. Among the entries of the vector $c_{Met2}$, $c_{ADP}$ is univocally determined by $c_{ATP}$ from Eq.(S5), so we can substitute the relation $c_{ADP}=1-c_{ATP}$ in all the equations obtained above. As far as the explicit expression of the internal ethanol concentration ($c_{{EtOH}_{in}}$ in $c_{Met2}$) is concerned, in principle it could be obtained from constraint (S15) but the task is computationally challenging. So, finally, the initial optimization problem can be rewritten as

$\begin{matrix} max \\ c_{{glc}_{in}}, c_{pyr}, c_{ATP},c_{{EtOH}_{in}},F,v_{rib} \end{matrix} \frac{1}{f_{hxt}\left( F, c_{Met2};c_{{glc}_{ex}} \right)+\sum_{j \in\left\{ rib, gly, resp, ferm \right\}} f_{j}\left( F, c_{Met2} \right)}$,

such that $g_{{EtOH}_{tr}}\left( c_{{EtOH}_{in}};c_{{EtOH}_{ex}} \right)=\psi_{EtOHtr}\left( F, c_{Met2} \right) v_{rib}$,

with 6 optimization variables and 1 constraint.

Although the degrees of freedom of the problem are actually 5, we verified by numerical simulation that $\lambda$ is “practically” independent of $v_{rib}$ in a glucose environment (i.e., $c_{{EtOH}_{ex}}=0$) so that the actual degrees of freedom of the optimization problem is 4. This fact is somehow implied by the tuning of the model parameters achieved in order to have $\upsilon_{gly2}\ll\upsilon_{gly}$ and $\upsilon_{resp2}\ll\upsilon_{resp}$ when $c_{{EtOH}_{ex}}=0$, that means, in order to make the transport of ethanol more favorable than its recycling in glucose environment. This property has been numerically verified by preliminary simulations performed for $c_{{EtOH}_{ex}}=0$ and $c_{{glc}_{in}}\epsilon(0, 300]$ (with $F \epsilon\left[ 0, 1 \right]$ and $c_{{glc}_{in}}, c_{pyr}, c_{ATP}\geq0$), revealing that $\eta\left( c_{Met2} \right)$, $\xi\left( c_{Met2} \right)$ assume consistently low values, at most of the order of magnitude of 10^-3^. This numerical property implies that $\psi_{j}\left( F, c_{Met2} \right)\approx\psi_{j}\left( F \right)$ (see equations (S13)) and then $f_{j}\left( F, c_{Met2} \right)=\psi_{j}\left( F \right)/g_{j}\left( c_{Met2} \right)$, $j \in\left\{ gly, resp, ferm, rib \right\}$ and $f_{hxt}\left( F, c_{Met2};c_{{glc}_{ex}} \right)=\psi_{hxt}\left( F \right)/g_{j}\left( c_{Met2};c_{{glc}_{ex}} \right)$. Therefore, since $g_{j}$, $j \in Prot$, are not dependent on $c_{{EtOH}_{in}}$, we can conclude that $\lambda$ is “practically” independent of $v_{rib}$ and then the numerical optimization can be performed w.r.t. the 5 variables $F, c_{{glc}_{in}}, c_{pyr}, c_{ATP},c_{{EtOH}_{in}}$ (fixing $v_{rib}$ to an arbitrary value, for instance, 1).

Similarly to the old version presented in [1], *MeGro-2* can also treat the fermentative ratio *F* as an input rather than an output (reducing the number of the optimization variables to 4), thus allowing the modeler to compute the optimal growth rate (as well as all the other model outputs) according to the fixed glucose and different values of *F*.

In order to perform data-driven numerical experiments, it is beneficial to derive a tool that allows the translation of experimental data into model inputs. In particular, from the model equations above, it is possible to derive an algebraic constraint relating the fermentative ratio *F* to the yield of ethanol $Y_{EtOH/glc}$. More in detail, according to the ethanol yield definition (S9) and the expressions of $\nu_{EtOHtr}$ and $\nu_{hxt}$ given by Eqs. (S13), we get

$Y_{EtOH/glc}=\frac{F\left( 1+\xi\left( c_{Met2} \right) \right)\left( 1.4+0.4\eta\left( c_{Met2} \right) \right) - \left( 1-F \right)\left( 1.4\xi\left( c_{Met2} \right)+8\eta\left( c_{Met2} \right)+5.4\xi\left( c_{Met2} \right)\eta\left( c_{Met2} \right) \right)}{F\left( 1+\xi\left( c_{Met2} \right) \right) + \left( 1-F \right)\left( 4+1.5\xi\left( c_{Met2} \right) \right)}$ . $\nu_{\mathrm{ferm}}= 1.4*{10}^{3}\frac{F}{1+10 \left( 1-F \right)} \nu_{\mathrm{rib}}$(S17)

Since, with the given parameter setting, it is $\eta\left( c_{Met2} \right), \xi\left( c_{Met2} \right) \ll1$ when $c_{{EtOH}_{ex}}=0$, $\nu_{EtOHtr}$ and $\upsilon_{hxt}$ in Eqs. (S13) can be reasonably approximated as:

$\nu_{EtOHtr}\approx{10}^{3}\frac{1.4 F}{11 - 10F}v_{rib}$, (S18)

$\upsilon_{hxt}\approx{10}^{3}\frac{4 - 3F}{11 - 10F}v_{rib}$, (S19)

providing the following approximated expression for the ethanol yield:

$Y_{EtOH/glc}\approx\frac{1.4 F}{4 - 3F}$. $\nu_{\mathrm{ferm}}= 1.4*{10}^{3}\frac{F}{1+10 \left( 1-F \right)} \nu_{\mathrm{rib}}$(S20)

Eq. (S20) is bijective. Thus, it can be inverted, providing the fermentative ratio *F* associated with a given ethanol yield:

$F\approx20\frac{Y_{EtOH/glc}}{7+ 15 Y_{EtOH/glc}}$. (S21)

This last equation is exploited to feed *MeGro-2* with the fermentative ratio associated with experimentally measured values of the ethanol yield (in this way, the experimental yield becomes an input of *MeGro-2*).

**3.b - The optimization problem associated to pure ethanol environment (**$\boldsymbol{c}_{\boldsymbol{glc}_{\boldsymbol{ex}}}\boldsymbol{=0}$**)**

When $c_{{glc}_{ex}}$ vanishes, from the kinetic equation (S6), it is $\upsilon_{hxt}=0$ and (by substituting $\upsilon_{hxt}=0$ in the first Eq. of (S4)) it is $\upsilon_{gly2}=\upsilon_{gly}$, which means $\eta\left( c_{Met2} \right)=1$ for any value of $c_{Met2}$. Since the condition $\upsilon_{hxt}=0$ is satisfied for any value of $c_{hxt}$, the optimization routine would bring $c_{hxt}$ (as well as $\alpha_{hxt}$) to zero for maximizing $\lambda$ (see Eq.(S11)). However, in order to perform more sound and realistic numerical experiments it is possible to fix the investment $\gamma_{hxt}$ on the transporter ‘hxt’ (see Eq. (S10)) based on experimental data: for populations growing in ethanol, we fixed it to 0.01. Therefore, adopting a data-driven approach, we can set $\gamma_{hxt}$ to the experimental percentage of proteins that the cell allocates for the class 'hxt,' so inferring the corresponding concentration from the data as

$c_{hxt}=\frac{\gamma_{hxt}}{\left( 1-\gamma_{hxt} \right)}\left( c_{gly}+c_{resp}+c_{ferm}+ c_{rib}*N_{prot/rib} \right)$. (S22)

Moreover, to simulate the growth in ethanol, it is reasonable to set $F=0$, which implies $\nu_{ferm}=c_{ferm}=\alpha_{ferm}=\gamma_{ferm}=0$. Note that such a condition is the best option for optimization if *F* is not fixed. Indeed, preliminary simulations showed that $\lambda$ increases for decreasing (fixed) values of *F* (while for high values of *F*, i.e., approximately *F* > 0.4, no feasible solution is found).

From Eqs. (S11) and (S22), and taking into account *F* = 0, we get the following expression for the growth rate

$\lambda=\frac{\left( 1-\gamma_{hxt} \right) \upsilon_{rib}}{c_{gly} +c_{resp} + c_{rib}(1-\gamma_{hxt}{+\gamma_{hxt}N}_{prot/rib})},$ (S23)

where ${0 \leq\gamma}_{hxt}\leq1$. Moreover, from Eqs.(S13-S14), we obtain

$\lambda=\frac{1}{\sum_{j \in\left\{ gly, resp, rib \right\}} \tilde{f}_{j}\left( 0, c_{Met2} \right)}$, with $\tilde{f}_{j}\left( 0, c_{Met2} \right)= \left. f_{j}\left( 0, c_{Met2} \right) \right|_{\eta\left( c_{Met2} \right)=1}$, (S24)

where the explicit dependence on $v_{rib}$ disappears again. So, the optimization problem in a pure ethanol environment is

$\begin{matrix} max \\ c_{{glc}_{in}}, c_{pyr}, c_{ATP},c_{{EtOH}_{in}},v_{rib} \end{matrix} \frac{1}{\sum_{j \in\left\{ gly, resp, rib \right\}} \tilde{f}_{j}\left( 0, c_{Met2} \right)}$,

such that $g_{{EtOH}_{tr}}\left( c_{Met2};c_{{EtOH}_{ex}} \right)=\psi_{EtOHtr}\left( 0, c_{Met2} \right) v_{rib}$,

which has 5 optimization variables and 1 constraint. So, also in this case, $v_{rib}$ could still indirectly influence the growth rate, from a theoretical point of view, since it is taken into account by the algebraic constraint (S15). Conversely, from preliminary numerical simulations, it turns out that $v_{rib}$ is not practically identifiable, and then it has been arbitrarily fixed to 1 as in the glucose case. Note that the chosen value of $v_{rib}$ influences the absolute value of $c_{{EtOH}_{in}}$, but it has no practical influence on the main *MeGro-2* outputs $\lambda, \rho$ (which are fractional quantities, not dependent on the absolute values of the other model variables, as explained above).

**4 - The *GroCy-2* module**

The equations of *GroCy-2* are briefly recap in this section for the reader’s convenience. All the details on the growth mechanism can be retrieved from the papers [3] and [4], while the details on the trigger mechanism and on the cell cycle timers can be found in [1].

The growth dynamics is modeled by the following ODEs describing the dynamics of proteins *P* and ribosomes *R*

$\frac{dR}{dt}={K_{1}\left[ \rho P-R \right]}^{+}- \frac{R}{\tau_{1}},$ (S25)

$\frac{dP}{dt}=K_{2}R- \frac{P}{\tau_{2}},$ (S26)

where $\left[ x \right]^{+}=\left\{ \begin{matrix} x, \mathrm{for} x>0 \\ 0, \mathrm{for} x\leq0 \end{matrix} \right.$, $K_{1}$ and $K_{2}$ are the rate constants of ribosome and protein synthesis, respectively, and $\tau_{1}$ and $\tau_{2}$ are the time constants of ribosome and protein degradation, respectively. The protein content *P* is expressed in number of polymerized amino acids. The mathematical analysis carried out on the growth equations (see [1,3,4] for details) has shown that, according to an approximation suggested by feasible model parameters, in balanced exponential growth condition

*ρ*K_2_ > 1/τ_2_*

the exponential growth rate comes out as an emergent property of the model, linked to other growth parameters according to the following relationship

* = ρ*K_2_ - 1/τ_2_* (S27)

Growth and cycle are linked through a molecular trigger given by the dynamical interplay between the nuclear Cln3 (the most upstream G_1_ Cdk1 activator) and Far1 (an inhibitor of the Cln3-Cdk1 complex). The model neglects Far1 outside the nucleus, whilst Cln3 is allowed to diffuse from nucleus to cytoplasm and *vice versa*. The interplay of the two molecular players above and of their complex Cln3-Far1is given by the following ODE system

$\frac{dCln3\_Far1}{dt}=\frac{k_{on}}{V_{nuc}} {Cln3}_{nuc} Far1- k_{off}Cln3/Far1$

$\frac{dFar1}{dt}=-\frac{k_{on}}{V_{nuc}} {Cln3}_{nuc} Far1+ k_{off}Cln3\_Far1-\eta\left( \frac{{Cln3}_{nuc}}{Cln3Far1} \right)Far1$

$\frac{d{Cln3}_{nuc}}{dt}=-\frac{k_{on}}{V_{nuc}} {Cln3}_{nuc} Far1+ k_{off}Cln3\_Far1+ k_{cn}{Cln3}_{cyt}- k_{nc}{Cln3}_{nuc}$ (S28)

${Cln3}_{cyt}= {Cln3}_{tot}- \left( {Cln3}_{nuc}+ Cln3\_Far1 \right)$

${Cln3}_{tot}= \theta P$,

where *V_nuc_*, *V_cell_* are nuclear and cell volume, respectively, and $\eta\left( x \right)$ refers to the clearance rate of Far1, supposed to increase when nuclear Cln3 exceeds Far1 according to a Hill-shape saturating function:

$V_{nuc}=hV_{cell}$, $V_{cell}=\frac{P}{H}$, $\eta\left( x \right)= \bar{\eta}\frac{x^{n_{F}}}{1+x^{n_{F}}}$. (S29)

As previously anticipated, the growth-to-trigger link is rendered by constraining the total amount of Cln3 to the overall protein content *P* (last Eq. in (S28)). Conversely, the trigger-to-cycle link is given by the time when the free nuclear Cln3 exceeds its inhibited form Cln3-Far1, as this event influences the length of the first part of the G_1_ phase on the basis of the mechanism described below.

The cycle module comprises three consecutive periods: *T_1_, T_2_*, and *T_B_*. *T_1_* and *T_2_* are named after the definition of [1] and their union provides the G_1_ phase: the former refers to the period a newborn cell takes to activate the G_1_/S regulon and is formally measured by the time the regulon inhibitor Whi5 takes to exit the nucleus; the latter refers to the time cyclins Clb5/6 (responsible for the onset of the S phase) take to get rid of their inhibitor Sic1. The length of *T_1_* is formally divided into two parts, *T_1_* = *T_1a_* + *T_1b_*. The first part *T_1a_* is the duration of the trigger mechanism, i.e. the period from cell birth till the time instant when nuclear Cln3 exceeds its inhibited form Cln3-Far1. Conversely, *T_1b_* starts when nuclear Cln3exceeds Cln3-Far1and its length is related to the size of the cell at the end of *T_1a_*, i.e.

$T_{1b}=max\left\{ T_{1b,min},W_{0}-W_{1}ln\left( P_{T_{1a}} \right) \right\}$, (S30)

with $P_{T_{1a}}$ denoting the size of the cell at the end of *T_1a_*, so that larger cells have smaller *T_1b_* and vice versa. Finally, *T_B_* includes the rest of the cycle, namely, phases S, G_2_, and M. Differently from *T_1a_* and *T_1b_*, *T_2_* and *T_B_* lengths are set by the modeler, and do not vary with the size of the cell.

When the 80% of the budded period has elapsed, the nuclear division (but not cell division) occurs and the G_1_* phase begins. The last 20% of timer *T_B_* refers to the G_1_^*^ phase, according to which two nuclei (and an undivided cytoplasm) are present in a yet single cell. At the beginning of the G_1_* phase a RESET function takes place producing a discontinuity in the nuclear players of the ODE describing the molecular trigger: an instantaneous synthesis of Far1 resets its content to a higher value (i.e. Far1_reset_, numerically equal to Far1(0)) and the total amount of Cln3-Far1, Far1 and nuclear Cln3 is partitioned in two nuclei whose volume is half of the original volume before RESET (*V_nuc_* = *hV_cell_*/2). So, in the G_1_* phase the variables *Cln3_Far1*, *Far1*, *Cln3_nuc_* describe the amounts of the three players in each one of the two nuclei. Moreover, in this last phase the Far1 degradation is inhibited (*η* = 0 in *Far1* dynamics, Eq.(S28)), and Cln3 diffusion from the cytoplasm into the nucleus is strongly reduced (*k_cn_* reduces of 5 orders of magnitude).

**4.a Asymmetrical division and genealogical age heterogeneity**

When a yeast cell buds, a chitin ring, called bud scar, builds up at the bud isthmus and remains on the parent cell after the bud has separated (see[5]). Bud scars on intact cells can be visualized by fluorescent dyes (Calcofluor, Primulin) in fluorescence microscopy . Since each new bud starts at a new site, it is possible to determine the number of bud scarspresent on the surface of a parent cell and consequently to establish the genealogical age ‘*k’* of the parent cell, meaning the age of the parent cell equal to the number of daughters it has generated (i.e., *k = s*). So, denoting by “*P_k_*" a parent cell of age*' k’*, a cell *P_1_* has one bud scar since it has completed a cycle, a cell *P_2_* has two bud scars since it has completed two cycles, and so on. On the other hand, a cell without bud scars (*s* = 0) is a daughter cell, and it has not yet completed a cycle. *iMeGroCy-2*, however, distinguishes also the genealogical age of the daughter cells: it can be 1 if the daughter is born from another daughter, while it is *k > 1* if the daughter is born from a parent *P_k-1_*. We denote a daughter of genealogical age '*k’* as “*D_k_*”. See the pedigree maps reported in Fig 2.

At division, each parent receives the mass it had at budding, whereas the mass synthesized during the budding phase goes to the newborn daughter. It follows that in parents, cell mass at budding increases with genealogical age. Experimental evidence shows that, as the genealogical age (i.e., the number of bud scars) increases, the increase in size at budding from one generation to the other decreases (reviewed in [5]). The reduction with genealogical age of the cell size increase has been explained by mechanical stress of the cell wall, which increases with cell size [1,4]. To account for the behavior mentioned above, when dealing with a parent cell *P_k_*, *iMeGroCy-2* reduces both rates of protein synthesis and time constant of protein degradation during the pre-budded period (G_1_ phase), with the amount of the reduction increasing according to the parent genealogical age. To this end, *K_2_* and *τ_2_* are decreased to lower and lower values during G_1_, according to the parent genealogical age. We define the *K_2_* and *τ_2_* parametersfor a parent cell with genealogical age ‘*k*' as *K_2_^k^* and *τ_2_^k^*. At the end of timer T_2_ - coincident with the end of the G_1_ phase and with the onset of the budded phase - the values of *K_2_^k^* and *τ_2_^k^* return to the nominal values of *K_2_* and *τ_2_* so that the parent cell *P_k_* grows again with the steady-state exponential rate approximately given by Eq. (S27). Daughter cells (of any genealogical age) are not affected by such mechanical stress; therefore, their growth parameters are not modified. We model the aforementioned mechanical stress by setting the growth rate *λ_k_* of a parent cell *P_k_* in the G_1_-phase as a percentage of *λ*, the growth rate shared by all daughter cells, and all parent cells in the budded phase. In particular, we fixed:* λ_1_* = 0.85***, *λ_2_* = 0.40***, *λ_3_* = 0.10***, *λ_4_* = 0.05***, *λ_5_* = 0.025***, and *λ_k_* = 0.005*** for *k* > 5. *τ_2_* is modified for parent cells *P_k_* within the G_1_-phase, i.e., *τ_2_^k^* is 50% of *τ_2_.*

**5 – Working of *iMeGroCy-2* and parameter setting**

The working mode of *iMeGroCy-2* aims at mimicking the cellular organization as a flow of information: different nutritional environments (experimental data) stimulate different metabolic responses (*MeGro-2* outputs), which eventually modulate downstream growth and cycle processes (*GroCy-2* setting). Experimental data entering *iMeGroCy-2* as model inputs are:

1. the ethanol, $c_{{EtOH}_{ex}}$, and glucose concentration, $c_{{glc}_{ex}}$, this last ranging 5%, 2%, 0.5%, 0.2%, 0.1%, 0.05%;
2. the yield of ethanol, *Y_EtOH/glc_*, for glucose concentrations.

These experimental data are integrated with the fermentative ratio *F* (i.e., the fraction of fermentative flux provided for different glucose concentrations), which *MeGro-2* univocally associates with the ethanol yield, Eq. (S21), thus providing a bijection from the external glucose concentration to *F*. In order to utilize a smoother *F*-vs-$c_{{glc}_{ex}}$ relationship, we used the best-fitting saturating function

*F* = *β **$c_{{glc}_{ex}}$/($c_{{glc}_{ex}}$+*α*), (S31)

where the values of *α* and *β* have been estimated by minimizing the mean square error with respect to the available experimental data. The estimated values of the above parameters are *α* = 0.6059 and *β* = 0.9790, and the fitting curve is depicted by the blue solid line in Fig 4B, where the squares correspond to the experimental data. The smoothed values of *F* will be exploited instead of the raw experimental ones to filter out measurement noises. In the case of ethanol nutrient, *F* = 0.

The fermentative ratio *F* and the glucose/ethanol concentration feed *MeGro-2* as inputs. In turn, *MeGro-2* solves an optimization algorithm providing the maximum growth rate *λ* (and the related ribosome-over-protein ratio *ρ*) according to the inputs above and to the model constraints that involve fluxes and metabolite concentrations (as stated in Section 3). Then, the pair (*λ, ρ*) is exploited to achieve the protein dynamics parameters (*K_2_*,*τ_2_*) from Eq. (S27), feeding *GroCy-2* as input parameters. The mathematical structure of *GroCy-2* (ODE + timers) allows to implement pedigree populations of cells, according to the asymmetrical division rules discussed in Subsection 4.a, providing *in silico* population features comparable with the experimental one.

**5.a – *MeGro-2* parameter setting**

A leading idea exploited when setting the *iMeGroCy-2* parameters is to use the largest set of common parameters for different nutritional environment, leaving apart just the ones that are apparent to vary from experimental facts. As a consequence, the same values of the *MeGro-2* parameters have been fixed and used for all the nutritional environments investigated: similarly to what happens in real cells, the metabolism sets the growth dynamics according to different nutritional environments. *MeGro-2* input parameters are reported in Table C. The parameter values of the metabolic routes that *MeGro-2* shares with *MeGro* are inherited from [1], where they had been chosen following the same criteria developed in [6] with minor modifications, mostly related to the use of different units. Concerning the new parameters, dealing with pure glucose environment (the only nutrient taken into account by *MeGro*), they have been set in order to ensure that *MeGro-2* produces outputs consistent with *MeGro*. More in detail, the parameters involved by the transport of ethanol in $\nu_{{EtOH}_{tr}}$ (i.e. $k_{cat,{EtOH}_{tr}}$ and $K_{M,{EtOH}_{tr}}$) are chosen to make the exit transport of $c_{{EtOH}_{in}}$ more favorable than the recycling throughout *ν_gly2_* and *ν_resp2_* in the absence of external ethanol (i.e., when $c_{{EtOH}_{ex}}=0)$. To this end, we fixed the parameters $k_{cat,{EtOH}_{tr}}$ and $K_{M,{EtOH}_{tr}}$to a high value (about 150 times $k_{cat,ferm}$) and, respectively, to a low value (about 100 times lower than $K_{M,ferm}$). So, when glucose is the only nutrient, it is $\upsilon_{gly2}\ll\upsilon_{gly}$ and $\upsilon_{resp2}\ll\upsilon_{resp}$, that is $\nu_{{EtOH}_{tr}} \approx\nu_{ferm}$.

**Table C. *MeGro-2* input parameters**

| **Parameter** | **Meas. unit** | **Value** | **Reference** |
| --- | --- | --- | --- |
| ***k_cat,hxt_*** | h^-1^ | 37492 | [1] |
| ***k_cat,gly_*** | h^-1^ | 4166 | [1] |
| ***k_cat,resp_*** | h^-1^ | 99 | [1] |
| ***k_cat,ferm_*** | h^-1^ | 6427 | [1] |
| ***k_cat,rib_*** | h^-1^ | 670 | [1] |
| ***k_cat,EtOHtr_*** | h^-1^ | 964080 | set by the authors |
| ***k_cat,gly2_*** | h^-1^ | 12497 | fine tuned |
| ***k_cat,resp2_*** | h^-1^ | 221 | fine tuned |
| ***K_M,hxt_*** | mM | 20 | [1] |
| ***K_M,gly_*** | mM | 0.2 | [1] |
| ***K_M,gly2_*** | mM | 0.2 | set by the authors |
| ***K_M,rib_, K_I,pyr_, K_I,glc_*** | mM | 1 | [1] |
| ***K_I2,glc_*** | mM | 1 | set by the authors |
| ***K_M,ADPgly_,K_M,ADPresp_***  ***K_M,resp_, K_M,ATPrib_*** | mM | 0.5 | [1] |
| ***K_M,ATPgly2_, K_M,ADPresp2_*** | mM | 0.5 | set by the authors |
| ***K_M,ferm_*** | mM | 5 | [1] |
| ***K_M,EtOHtr_*** | mM | 0.05 | set by the authors |
| ***K_M,resp2_*** | mM | 0.05 | fine tuned |
| ***Nprot/rib*** | #prot/#rib | 79 | [7] |
| ***Naa/prot_rib*** | #aa/#prot_rib_ | 157.5 | yeastgenome.org |
| ***Naa/prot*** | #aa/#prot | 441.5 | yeastgenome.org |

Conversely, as regards the simulation of *MeGro-2* in ethanol environment, we set the kinetic parameters of the new branches of *MeGro-2* fed by the internal ethanol, i.e. $\upsilon_{gly2}$ (*k_cat,gly2_, K_M,gly2_, K_M,ATPgly2_, K_I2,glc_*) and $\upsilon_{resp2}$ (*k_cat,resp2_, K_M,resp2_, K_M,ADPresp2_*) in order to replicate the experimental data on the Mass Duplication Time (MDT) of the WT CEN.PK yeast strain in an ethanol 2%. To this end, without loss of generality*,* we set the values of the Michaelis-Menten parameters involved in $\upsilon_{gly2}$ equal to their homologues values in $\upsilon_{gly}$, whilst the other free *MeGro-2* parameters have been tuned to best fit the aforementioned experimental value. It worths noticing that the achieved *k_cat_* parameters are compatible with the bounds coming from proteomic-constrained GEMs, where *k_cat_* parameters span 11 orders of magnitude in *Saccharomyces cerevisiae*, with median values in 70.9s^-1^ = 255*10^3^h^-1^ [8]. Moreover, differently from the glucose environment where $\gamma_{hxt}$ is a free variable of the optimization algorithm, according to what explained in Subsection 3.b, we fix $\gamma_{hxt}=0.01$ for simulations in ethanol.

Finally, as regards the number of ribosomal proteins per ribosome, $N_{prot/rib}$, the average number of amino acids per ribosomal protein, $N_{aa/prot\_rib}$, and the average number of amino acids per generic protein $N_{aa/prot}$, they have been set according to literature data [7] (see also yeastgenome.org).

As the graph below shows (Fig B), *MeGro-2* accurately predicts the growth rate *λ* in all the simulated conditions. This indicates that the parameter set used in *MeGro-2* captures growth rate modulations of yeast cells in various environmental conditions, which gives solid grounds for the robustness of this parameter set.

**Fig B. Correlation between experimental and *iMeGroCy-2* simulated values for the growth rate** *λ* **parameter**. Values (obtained under various nutritional conditions) are reported as relative to the 2 % glucose growth media, taken as reference. The green bisector line indicates the ideal, perfect correlation between the two data series.

**5.b – GroCy-2 parameter setting**

*MeGro-2* outputs help to set some of *GroCy-2*'s growth parameters. For any nutrient condition, *MeGro-2* provides the optimal pair (***λ****,ρ*), so allowing to directly feed *ρ* into *GroCy-2* and to set the protein rate constant $K_{2}$ by solving Eq. (S27) for given values of ***λ****, ρ* (coming from *MeGro-2*), and $\tau_{2}$, whose value is set to a biologically meaningful value in [1] for glucose 2%, and here is applied to any nutritional environment. Clearly, there exist infinite pairs (*K_2_*,$\tau_{2}$) satisfying the constraint of Eq. (S27), whose manifold is reported in the blue line in the figure below for the glucose 2% case (Fig C). The five red markers refer to the pair of values chosen for the glucose 2% (the central marker) and for other 4 choices of pairs (*K_2_*,$\tau_{2}$) satisfying constraint ***λ*** = ρ**K_2_* - 1/$\tau_{2}$, obtained by arbitrarily varying $\tau_{2}$ as reported in Table D. It is apparent from simulations results reported in Table D that the model is robust with respect to the way the growth parameters are chosen, since there are only negligible variations in population outputs.


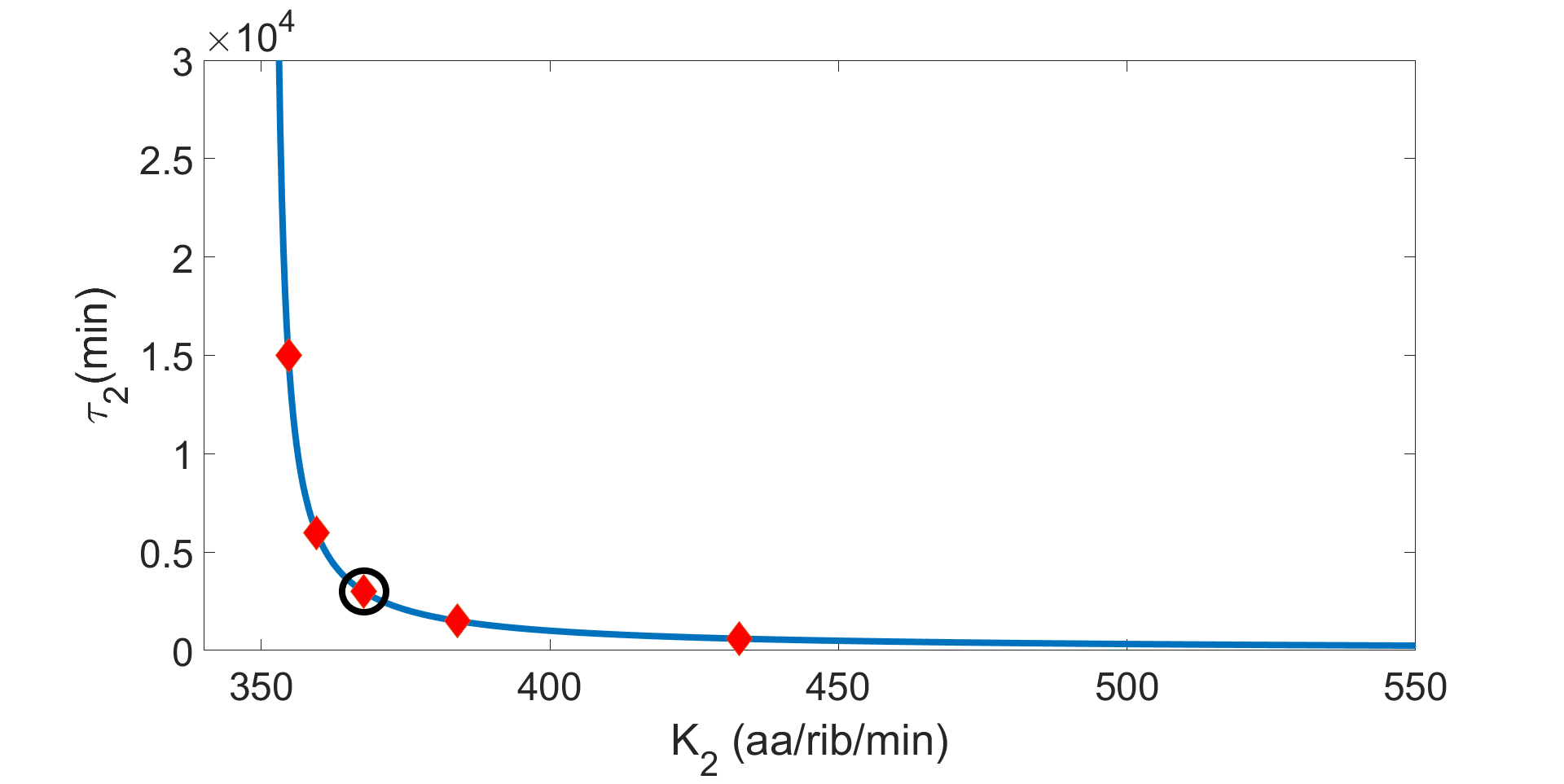


**Fig C. Pairs of (*K_2_*,**$\boldsymbol{\tau}_{\boldsymbol{2}}$**) satisfying the constraint of Eq.(S27) for fixed values of (*λ,ρ*).** The red markers refer to the values of $\tau_{2}$coming from Table D; the black circle refers to the chosen setting

**Table D. *iMeGroCy-2* population output parameters for pairs** (*K_2_*,$\tau_{2}$) satisfying the constraint of Eq. (S27) for fixed values of (*λ,ρ*).

| Glucose 2% | <P>, aa | MDT, min | <TG1>, min | <TG1D>, min | <TG1P>, min | Budding  Index |
| --- | --- | --- | --- | --- | --- | --- |
| *_2_* = 15000min  *K_2_* = 354.68 aa/rib/min | 4.46e10 | 99.24 | 14.30 | 16.70 | 11.81 | 0.81 |
| *_2_* = 6000min  *K_2_* = 359.56 aa/rib/min | 4.43e10 | 99.24 | 14.36 | 16.79 | 11.83 | 0.81 |
| *_2_* = 3000min  *K_2_* = 367.69 aa/rib/min | 4.57e10 | 99.29 | 14.34 | 16.78 | 11.81 | 0.81 |
| *_2_* = 1500min  *K_2_* = 383.95 aa/rib/min | 4.39e10 | 99.32 | 14.52 | 17.07 | 11.87 | 0.81 |
| *_2_* = 600min  *K_2_* = 432.73 aa/rib/min | 4.36e10 | 99.42 | 14.77 | 17.46 | 11.95 | 0.80 |

Similarly to $\tau_{2}$, also the other growth parameters unrelated to *MeGro-2* (namely $\tau_{1}$, $\tau_{2}^{k}$, *K_1_*), are set according to the values inherited from [1], where they had been set for glucose 2%, and here they are not supposed to vary according to different media, as we do not have experimental evidences suggesting a variation with respect to the nutrient. The same reasoning adopted for $K_{2}$ works for parameters $K_{2}^{k}$ related to the unbudded phase of parent cells of any genealogical age *k*: they are computed by solving Eq. (S27) with the growth rate ***λ***suitably reduced of a percentage depending of the number of scars (see Subsection 4a for details).

Concerning the remaining *GroCy-2* parameters, the ones related to glucose 2% environment are as well inherited from [1]; regarding to the ones related to richer (glucose 5%) or poorer (glucose 0.5%, 0.2%, 0.1%, 0.05%, ethanol) media, we chose to keep them fixed to the values of glucose 2% in the absence of experimental evidence suggesting a variation with respect to the nutrient. Indeed, a change in the values of the growth parameters coming from *MeGro-2* alone, satisfactorily reproduces the output features of yeast populations growing in 5% and 0.5% glucose (Fig 4C-E). For lower glucose concentrations (i.e. glucose 0.2%, 0.1%, 0.05%) and ethanol, this approach captures the reduction in overall dimension and the decrease in growth rate. Still, it leaves space for further improvements, as shown for protein distributions in Fig D.


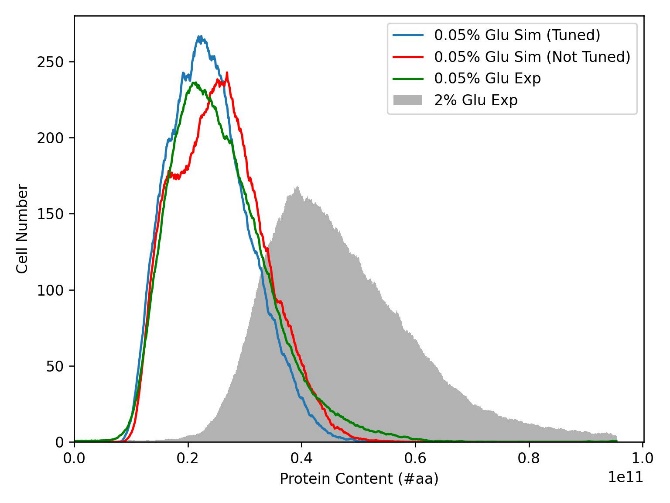

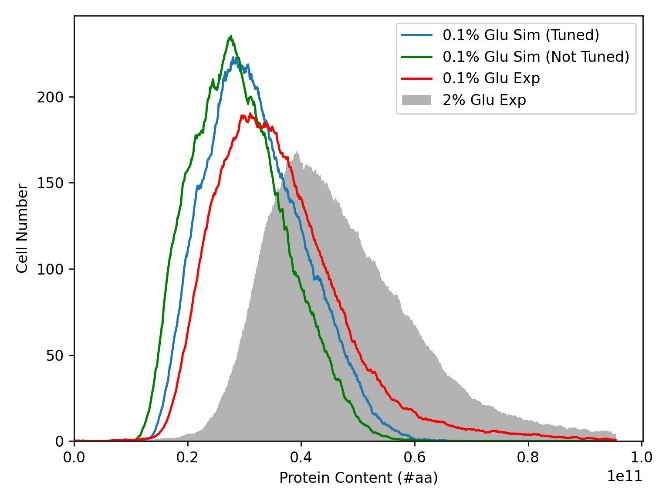


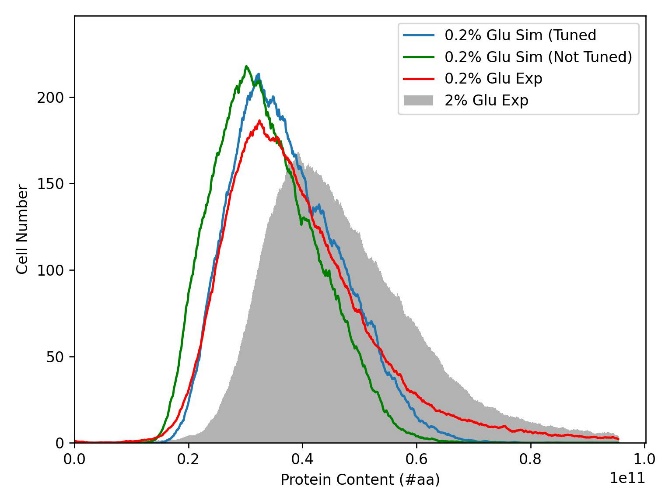

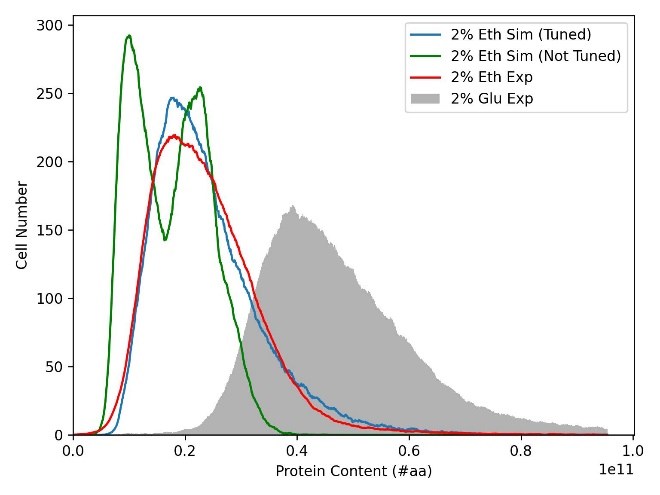


**Fig D. Protein distributions for different low glucose and ethanol concentrations.** Curves drawn for *GroCy-2* model parameters inherited from glucose 2%, without any further tuning than the one provided by the *MeGro-2* interconnection (green curves), with also a further (though minimal) tuning (blue curves), compared to the experimental ones (red curves). The grey shade refers to the glucose 2% experimental distribution reference.

Below are reported details on which parameters we decided to vary and why. As regards the molecular trigger mechanism, the only exception is given by the initial amount of Far1, which is set equal to 240 molecules for glucose environments from 0.1% up to 5%, and it is reduced in poorest mediums to 170 molecules for 0.05% glucose concentration or to 110 molecules for ethanol 2%. This is consistent with reported experimental data that indicate that the level of Far1 decreases at slow growth rates [9].The parameters *H* (protein-over-volume ratio) and $\theta$ (Cln3-over-protein ratio) are also slightly decreased in ethanol according to [1,9]. Regarding the timer lengths, budding yeast literature [10] and our own experiments (see Table L, panel A) tell us that the budded and the unbudded phases undergo very slight variations according to different glucose environments, showing instead a not negligible variation when passing from glucose to ethanol media. Straightforwardly, we decided to:

- cluster *T_B_* length into high glucose (5%, 2%, 0.5%), low glucose (0.2%, 0.1%, 0.05%) and ethanol media according to our experimental data (see Table L, panel A and [1] from which the glucose 2% value is inherited);
- keep *T_2_* length fixed for all glucose concentrations to the value set in [1] for glucose 2%, allowing it to increase only for ethanol media;
- keep *T_1_* input parameters (*W_0_, W_1_, T_1bmin_*) fixed to the values set in [1] for glucose 2% for quite all glucose concentrations, except for *W_0_* that is varied for glucose 0.05% and ethanol, and for *T_1bmin_* that is varied only for ethanol; this few last adjustments are made in order to catch the marked extension of the G_1_ length in the poorest media, as suggested by experimental data (see Table L, panel A).

Table E reports the numerical values used for all the glucose concentrations and for the ethanol environment.

**Table E. *iMeGroCy-2* input parameters**

| **GroCy-2 inputs (computed from MeGro)** | | | | | | | | |
| --- | --- | --- | --- | --- | --- | --- | --- | --- |
| **Parameters** | **Meas. unit** | **EtOH 2%** | **glc 0.05%** | **glc 0.1%** | **glc 0.2%** | **glc 0.5%** | **glc 2%** | **glc 5%** |
|  |  | $\boldsymbol{F}\boldsymbol{=0}$ | $\boldsymbol{F}\boldsymbol{=0.804}$ | $\boldsymbol{F}\boldsymbol{=}\mathbf{0.883}$ | $\boldsymbol{F}\boldsymbol{=0.928}$ | $\boldsymbol{F}\boldsymbol{=0.958}$ | $\boldsymbol{F}\boldsymbol{=}\mathbf{0.974}$ | $\boldsymbol{F}\boldsymbol{=0.977}$ |
|  |  | $\boldsymbol{\gamma}_{\boldsymbol{hxt}}\boldsymbol{=0.01}$ | **free invest.** | **free invest.** | **free invest.** | **free invest.** | **free invest.** | **free invest.** |
| ***ρ*** | rib/aa | 8.327e-06 | 1.964e-05 | 2.015e-05 | 2.050e-05 | 2.075e-05 | 2.084e-05 | 2.085e-05 |
| ***K****_2_* | aa/rib/min | 526.80 | 304.17 | 317.17 | 332.37 | 351.10 | 367.69 | 372.07 |
| ***K****_2_^1^* | aa/rib/min | 493.82 | 278.06 | 288.62 | 301.21 | 316.91 | 330.93 | 334.65 |
| ***K****_2_^2^* | aa/rib/min | 274.77 | 148.82 | 153.33 | 158.96 | 166.15 | 172.67 | 174.41 |
| ***K****_2_^3^* | aa/rib/min | 128.74 | 62.66 | 63.14 | 64.12 | 65.64 | 67.16 | 67.58 |
| ***K****_2_^4^* | aa/rib/min | 104.40 | 48.30 | 48.11 | 48.32 | 48.89 | 49.58 | 49.78 |
| ***K****_2_^5^* | aa/rib/min | 92.23 | 41.12 | 40.60 | 40.42 | 40.51 | 40.78 | 40.87 |
| ***K****_2_^s^, s > 5* | aa/rib/min | 82.49 | 35.37 | 34.58 | 34.09 | 33.81 | 33.75 | 33.75 |
| **GroCy-2 inputs (tuned by the modeler)** | | | | | | | | |
| **Parameters** | **Meas. unit** | **EtOH 2%** | **glc 0.05%** | **glc 0.1%** | **glc 0.2%** | **glc 0.5%** | **glc 2%** | **glc 5%** |
| ***Far1(0)*** | molec | 110 | 170 | 240 | 240 | 240 | 240 | 240 |
| ***Cln3****_nuc_****(0)*** | molec | 0 | 0 | 0 | 0 | 0 | 0 | 0 |
| ***Cln3Far1(0)*** | molec | 0 | 0 | 0 | 0 | 0 | 0 | 0 |
| ***Far1****_reset_* | molec | 110 | 170 | 240 | 240 | 240 | 240 | 240 |
| ***K****_1_* | min^-1^ | 1 | 1 | 1 | 1 | 1 | 1 | 1 |
| ***τ****_1_* | min | 4000 | 4000 | 4000 | 4000 | 4000 | 4000 | 4000 |
| ***τ****_2_* | min | 3000 | 3000 | 3000 | 3000 | 3000 | 3000 | 3000 |
| ***τ****_2_^s^, s ≥ 1* | min | 1500 | 1500 | 1500 | 1500 | 1500 | 1500 | 1500 |
| ***k****_on_* | (molec/L)^-1^/min | 1.63e-15 | 1.63e-15 | 1.63e-15 | 1.63e-15 | 1.63e-15 | 1.63e-15 | 1.63e-15 |
| ***k****_off_* | min^-1^ | 25 | 25 | 25 | 25 | 25 | 25 | 25 |
| ***h*** | - | 0.07 | 0.07 | 0.07 | 0.07 | 0.07 | 0.07 | 0.07 |
| ***H*** | aa/L | 6.18e23 | 7.09e23 | 7.09e23 | 7.09e23 | 7.09e23 | 7.09e23 | 7.09e23 |
| ***n****_F_* | - | 10 | 10 | 10 | 10 | 10 | 10 | 10 |
| $\bar{\eta}$ | min^-1^ | 1 | 1 | 1 | 1 | 1 | 1 | 1 |
| ***Θ*** | molec/aa | 2.66e-8 | 3.02e-8 | 3.02e-8 | 3.02e-8 | 3.02e-8 | 3.02e-8 | 3.02e-8 |
| ***k****_cn_* | min^-1^ | 1.5 | 1.5 | 1.5 | 1.5 | 1.5 | 1.5 | 1.5 |
| ***k****_nc_* | min^-1^ | 0.6 | 0.6 | 0.6 | 0.6 | 0.6 | 0.6 | 0.6 |
| ***T****_1b,min_* | min | 15 | 1 | 1 | 1 | 1 | 1 | 1 |
| ***W****_0_* | min | 1466 | 1496 | 1503 | 1503 | 1503 | 1503 | 1503 |
| ***W****_1_* | min | 62.1 | 62.1 | 62.1 | 62.1 | 62.1 | 62.1 | 62.1 |
| ***T****_2_* | min | 35 | 10 | 10 | 10 | 10 | 10 | 10 |
| ***T****_B_ (simulating strain CEN.PK in Fig 4, Fig O and W303 in Fig P)* | min | 133 | 90 | 90 | 90 | 85 | 85 | 85 |
| ***T****_B_ (simulating CEN.PK in Figs 7-8)* | min | 133 | - | 87 | - | - | 81 | - |
| **Protein content for the initial cells of the 10 germinal lines providing the *iMeGroCy-2* population**  **(tuned by the modeler)** | | | | | | | | |
| ***P_0_(D_1_)_gl1_*** | aa | 1.46e10 | 1.46e10 | 2.10e10 | 1.99e10 | 2.54e10 | 3.03e10 | 3.76e10 |
| ***P_0_(D_1_)_gl2_*** | aa | 1.15e10 | 1.15e10 | 2.20e10 | 2.30e10 | 2.21e10 | 3.18e10 | 2.71e10 |
| ***P_0_(D_1_)_gl3_*** | aa | 1.03e10 | 1.03e10 | 1.86e10 | 2.73e10 | 2.44e10 | 2.68e10 | 3.37e10 |
| ***P_0_(D_2_)_gl4_*** | aa | 1.48e10 | 1.48e10 | 2.67e10 | 2.85e10 | 3.01e10 | 3.14e10 | 2.77e10 |
| ***P_0_(D_3_)_gl5_*** | aa | 1.66e10 | 1.66e10 | 2.40e10 | 2.64e10 | 2.80e10 | 3.13e10 | 3.50e10 |
| ***P_0_(P_1_)_gl6_*** | aa | 1.62e10 | 1.62e10 | 2.18e10 | 2.73e10 | 3.04e10 | 3.65e10 | 4.03e10 |
| ***P_0_(P_1_)_gl7_*** | aa | 2.08e10 | 2.08e10 | 2.38e10 | 3.06e10 | 3.26e10 | 3.57e10 | 3.51e10 |
| ***P_0_(P_1_)_gl8_*** | aa | 1.92e10 | 1.92e10 | 2.29e10 | 2.89e10 | 3.39e10 | 3.32e10 | 3.73e10 |
| ***P_0_(P_2_)_gl9_*** | aa | 2.63e10 | 2.63e10 | 2.77e10 | 2.95e10 | 3.13e10 | 3.87e10 | 3.35e10 |
| ***P_0_(P_3_)_gl10_*** | aa | 2.49e10 | 2.49e10 | 3.00e10 | 3.52e10 | 3.11e10 | 3.95e10 | 4.42e10 |

**5.c Simulations of cell populations through *iMeGroCy-2***

*iMeGroCy-2* can generate pedigree populations of yeast, distinguishing among the many features of daughter and parent cells.

For any chosen glucose concentration, a preliminary “*long period*” simulation is run, starting from a newborn daughter cell and letting it evolve cycle after cycle for enough time to reach steady-state conditions (i.e., exponential growth): in this way, the population properties do not depend on the properties of the initial daughter cell. This single germinal line is exploited to build up a population of 10 germinal lines, each seed starting from 10 different initial cells (sampled at the end of the "*long period*” simulation) and simulated over time to generate a complete pedigree. The 10 initial cells include daughter or parent cells of different genealogical ages (*k* = 1,2,3,…), different initial protein and ribosome content (*P_0_, R_0_*), and different positions in the cell cycle at the time of their virtual sampling. We chose 5 daughters (3*D_1_*, 1*D_2_*, 1*D_3_*) and 5 parents (3*P_1_*, 1*P_2_*, 1*P_3_*) according to the daughter/parent cell distributions of the first "*long period*” simulation. In particular, for any chosen glucose concentration, the protein contents of the 10 initial cells, *P_0_*(*D_k_*), *P_0_*(*P_k_*), *k* = 1,2,3, are sampled from lognormal distributions, with mean values and standard deviations set according to the protein contents distributions of *D_k_* and *P_k_* cells of the “*long period*” germinal line population in steady-state, grown in the same glucose environment. The selected protein contents of the 10 initial cells are reported in Table E for all the glucose concentrations.

Regarding the position in the cell cycle, each germinal line initial cell is supposed to be virtually sampled at Δ minutes from its birth: without loss of generality, we chose Δ = 10*n min, with n = 0, 1, …, 9, thus assuming to have 10 initial cells sampled each 10 min over an average cycle length of 100 min. Such a choice ensures the maximum entropy among the cells and reduces the probability of unnatural synchronizations. Each pedigree population line is kept proliferating until it reaches a steady state, with a final number of N cells each, that is, a whole population of 10N cells. Number N is strictly related to the generations required to reach a steady state and may depend on the glucose concentration.

We adopted the following additional rules to simulate the growth of the population:

1. all cells evolve according to the equations and rules described for individual cells;
2. the lengths of timers T_2_ and T_B_ are drawn from a lognormal distribution with an average value equal to the values reported in Table E and a Coefficient of Variation (CV) of 0.05 (equal for daughters and parents of any genealogical age);
3. timer T_1b_ length is drawn from a lognormal distribution with the average value given by a function of the protein content at the onset of T_1b_ (see Eq.(S30)) and a given CV of 0.05;
4. any daughter cell coming at division gives birth to a pair of newborn daughter/parent cells with unitary genealogical age, i.e., *D_1_* and *P_1_*; a parent cell *P_k_* gives birth to a pair of newborn daughter/parent cells with incremental genealogical age, i.e., *D_k+1_* and *P_k+1_*; see Fig 1A and Fig 2;
5. at cellular division, each cell is substituted by a pair of newborn daughters and parents;
6. the newborn parent cell starts with an initial protein content *P_0_(P)* drawn from a lognormal distribution with an average value given by the critical size *P_s_* achieved by the parent cell at the onset of the budded phase. The CV is taken to be smaller and smaller for newborn parent cells of increasing genealogical age, that is 0.05 for a parent born from a daughter or from a parent *P_k_*, k = 1,…,5, while CV = 0.04, 0.03 and 0.02 for a parent born from *P_6_*, *P_7_* and *P_k_*, k > 7, respectively; the newborn daughter cell starts with an initial protein content *P_0_(D)* given by the difference of the protein content at division and the newborn parent cell initial size;
7. the newborn daughter/parent cells start with the initial ribosome content determined (from the overall mother cell ribosome content at division *R_CD_*) in order to ensure that the ratios *R_0_(D)/R_CD_* and *R_0_(P)/R_CD_* are equal to the ratios *P_0_(D)/P_CD_* and *P_0_(P)/P_CD_* respectively.

It is worth notice that the population model incorporates noise only according to items ii), iii) and vi). The CVs refer to single-cell variability, providing differences in the properties of individual cells of a given population; these CVs are shared by all nutritional environment (as well as by all simulated mutants) managing to satisfactory resemble the different protein distributions in a unique fashion without further adjustments. We recall that even in the absence of any added variability to timers and parent/daughter protein partition at division, a nontrivial variability in the mass duplication time (i.e., the time from birth to division) appears as well. This heterogeneity - apparent between parents and daughters and within parent and, more so, daughter cells is mainly due to differences in the length of the G_1_ phase. These observations are summarized in Table F reported below.

**Table F. *Impact of variability in MDT and protein partitioning at division***

|  | Daughters | | | Parents | | | |
| --- | --- | --- | --- | --- | --- | --- | --- |
|  | Protein content  (average ± SD) | Cycle length (average ± SD) | G_1_ length  (average ± SD) | Protein content  (average ± SD) | Cycle length  (average ± SD) | G_1_ length  (average ± SD) |  |
| CV=0 | 4.03e10±1.01e10 | 100.2 ± 3.1 | 15.2 ± 3.1 | 4.44e10±1.03e10 | 96.3 ± 0.04 | 11.3 ± 0.04 |  |
| CV=0.05 | 4.28e10±1.12e10 | 100.8 ± 7.1 | 15.7 ± 5.7 | 4.88e10±1.18e10 | 96.7 ± 4.4 | 11.5 ± 1.25 |  |

The outcomes of the population simulations can be clustered into three classes. On the one hand, we have a set of population variables that evolve with time, like (i) the number of cells, (ii) total protein content, (iii) total ribosome content, and (iv) the budding index, defined as the fraction of budded cells. According to these four outputs, further outcomes can be derived, such as the population exponential growth (according to which the MDT is readily derived as MDT = log(2)/λ) or other time-varying variables, such as the average protein (and ribosome) content and the budding index. It is worth noting that the growth rate coming out from MeGro-2 outputs (that is exploited to set the growth module parameters *τ_2_* and *K_2_*) is formally different from the growth rate coming out as an emergent property of the pedigree population of *iMeGroCy-2*; nonetheless, these 2 values are strongly correlated (Pearson coefficient ≈1; see Fig E).


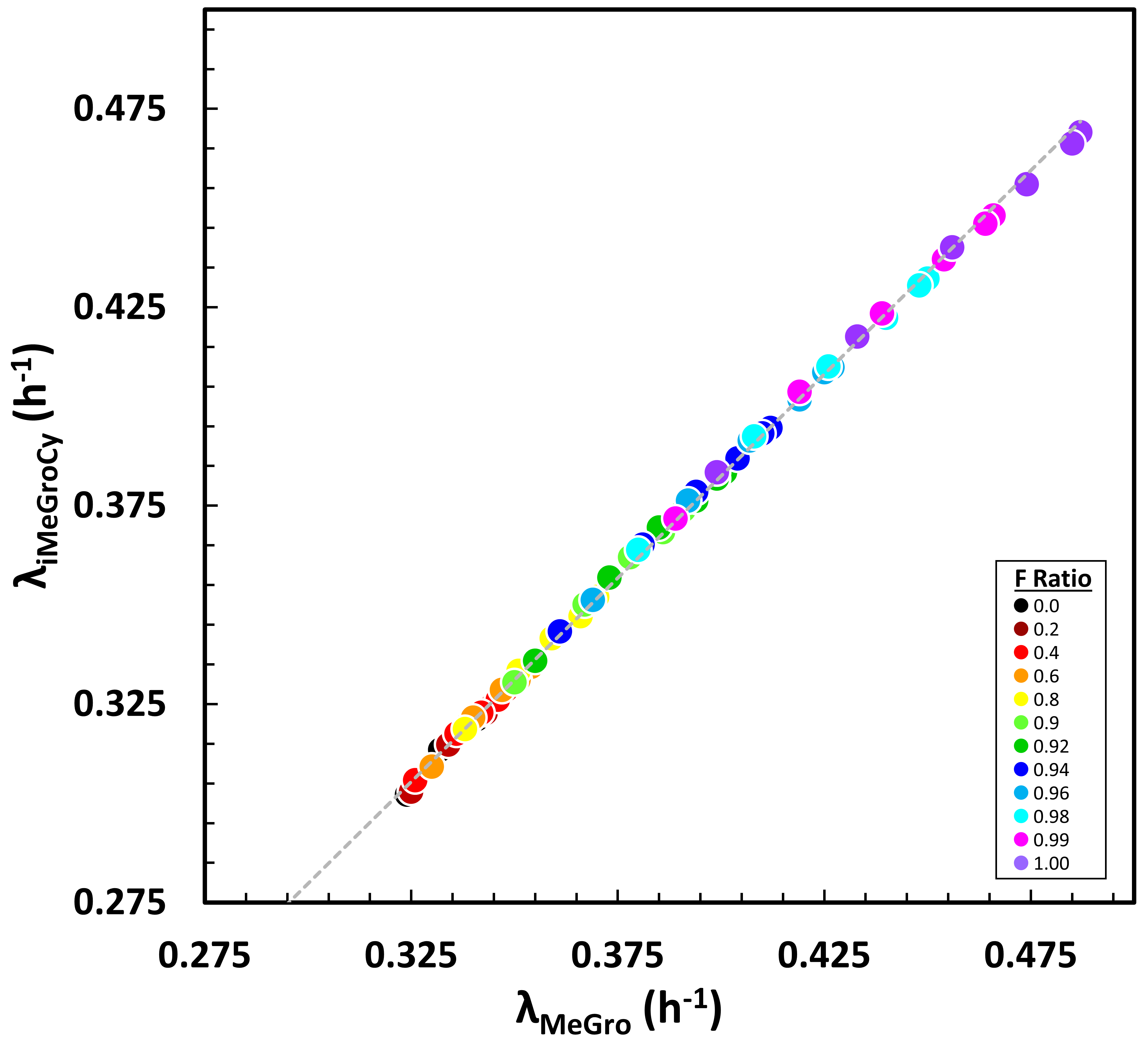


**Fig E. Correlation between *MeGro-2*-vs-*iMeGroCy-2* growth rate**. Dots are growth rate λ values obtained under different fermentative conditions (color-coded), F ratio between [0, 1]) in various growth media (from 0.05% to 5% glucose).

Fig F, panels A-D reports some of these evolutions for 2% and 0.05% glucose, with red curves referring to the average value from the 10-line pedigree populations.

**Fig F. Simulated parameters in evolving and steady state populations**.

**A, B:** time course of the average protein content, for fast (glucose 2%, panel A) and slow (glucose 0.05%, panel B) growth conditions. **C, D:** time course of the budding index, for fast (glucose 2%, panel C) and slow (glucose 0.05%, panel D) growth conditions. The convergence rate depends on the kind of population meta-parameters. The black lines show the time behavior of <P> and BI related to the 10 clusters, while the red line depicts their average value. **E:** simulated vs experimental protein distribution of a yeast population growing in 2% glucose.

Other outputs are provided by selecting a set of population cells. To this end, we have considered a time instant when the population under investigation becomes of about 10*N* cells (with *N* dependent on the glucose environment) and computed statistics concerning the whole cycle of these cells, such as the timer lengths, their critical size or their size at birth and at division. All these values are computed in average and standard deviation and can refer to the whole population or to subsets such as daughter or parent cells (or even subsets like *D_1_, D_2_, P_1_, P_2_* cells, etc.). Most of these outputs can be found in Table G for different glucose concentrations and compared to their corresponding experimental values.

The third class of outputs deals with the protein distributions. We have randomly extracted *M* = 50,000 cells from the complete set of 10*N* cells at a given time *t*. The *M* cells are "*frozen*" from the population simulation to their protein content at the selected time *t*. Each cell is tagged as a daughter or a parent cell; thus, distinct daughter and parent protein distributions can be computed. Fig 4C-I and Fig F, panel E report the protein distributions compared to the corresponding experimental distributions of about 50,000 cells for different glucose and ethanol environments.

**Table G. *iMeGroCy-2* population output parameters. Light cyan rows refer to control experimental data**

| **Parameters** | **Meas. Unit** | **EtOH 2%** | **glc 0.05%** | **glc 0.1%** | **glc 0.2%** | **glc 0.5%** | **glc 2%** | **glc 5%** |
| --- | --- | --- | --- | --- | --- | --- | --- | --- |
| ***MDT*** | min | 187 | 131 | 121 | 112 | 104 | 99 | 98 |
| ***MDT*** | min | 195 ± 5 | 117 ±4 | 109 ± 3 | 104 ±1 | 99 ± 2 | 95 ± 1 | 96 ± 2 |
| **2^-^*^TD/MDT^* + 2^-^*^TP/MDT^*** | - | 1.0000 | 0.996 | 0.999 | 1.000 | 0.999 | 0.999 | 0.997 |
| **2^-^*^TD/MDT^* + 2^-^*^TP/MDT^*** | - | 1.007 ± 0.003 | 1.008 ± 0.004 | 1.006 ± 0.001 | 1.007 ± 0.002 | 1.008 ± 0.002 | 1.003 ± 0.002 | 1.003 ± 0.000 |
| ***T_G1_(D)*** | min | 58 | 55.6 ± 12.6 | 41.8 ± 10.5 | 28.8 ± 8.5 | 25.2 ± 8.0 | 15.9 ± 5.8 | 14.1 ± 4.7 |
| ***T_G1_(D)*** | min | 71 | 35 | 29 | 22 | 23 | 21 | 17 |
| ***T_G1_(P)*** | min | 52 | 28.5 ± 9.5 | 20.7 ± 7.2 | 14.9 ± 4.6 | 13.3 ± 3.4 | 11.5 ± 1.3 | 11.3 ± 0.9 |
| ***T_G1_(P)*** | min | 42 | 11 | 12 | 8 | 7 | 9 | 6 |
| ***T_B_*** | min | 131.80 | 90 ± 5 | 90 ± 5 | 90 ± 5 | 85 ± 4 | 85 ± 4 | 85 ± 4 |
| ***T_B_*** | min | 137 ± 1 | 93 ± 2 | 88 ± 1 | 88 ± 1 | 83 ± 1 | 82 ± 2 | 84 ± 1 |
| ***T_D_*** | min | 190 | 146 ± 13 | 132 ± 12 | 119 ± 10 | 110 ± 9 | 101 ± 7 | 99 ± 6 |
| ***T_D_*** | min | 208 ± 6 | 128 ± 4 | 117 ± 4 | 110 ± 3 | 106 ± 2 | 103 ± 1 | 101 ± 1 |
| ***T_P_*** | min | 183 | 119 ± 11 | 111 ± 9 | 105 ± 6 | 99 ± 5 | 97 ± 4 | 97 ± 4 |
| ***T_P_*** | min | 179 ± 5 | 104 ± 4 | 100 ± 3 | 96 ± 1 | 90 ± 3 | 91 ± 1 | 90 ± 3 |
| ***<P>/<P>_2%_*** | - | 0.51 | 0.53 | 0.71 | 0.83 | 0.87 | 1.0 | 1.08 |
| ***<P>/<P>_2%_*** | - |  | 0.60 | 0.70 | 0.79 | 0.86 | 1.0 | 1.13 |

**5.d - Sensitivity analysis**

In this section we performed a sensitivity analysis of *iMeGroCy-2* w.r.t. the model parameters of *MeGro-2* and *GroCy-2,* changing a single parameter at a time.

First of all, we analyzed the variability of the most important interconnection quantity between *MeGro-2* and *GroCy-2*, i.e., the growth rate *λ*, w.r.t. the variation of some crucial parameters of *MeGro-2*, i.e. the catalytic coefficients *k_cat_* of the kinetic equations of the fluxes. Then, we analyzed the variability of the *iMeGroCy-2* population features w.r.t. the *GroCy-2* parameters.

The sensitivity analysis of *λ* w.r.t. *k_cat,x_*, *x ∈ Prot2*, has been performed by fixing the *MeGro-2* inputs *F* and $c_{{glc}_{ex}}$ to the experimental values related to 2% glucose reported in the heading of Table E. The results of this analysis are depicted in Fig G: each panel shows the variations of *λ* w.r.t. its value in 2% glucose when the nominal *k_cat_* (reported in Table C) is varied of 0.01x, 0.1x, 10x, 100x. The analysis shows that the *k_cat,EtOH_*, *k_cat,resp2_*, *k_cat,gly2_* have marginal effects on *λ* while *k_cat,ferm_*, *k_cat,gly_* are the catalytic coefficients that influence more the *MeGro-2* output.


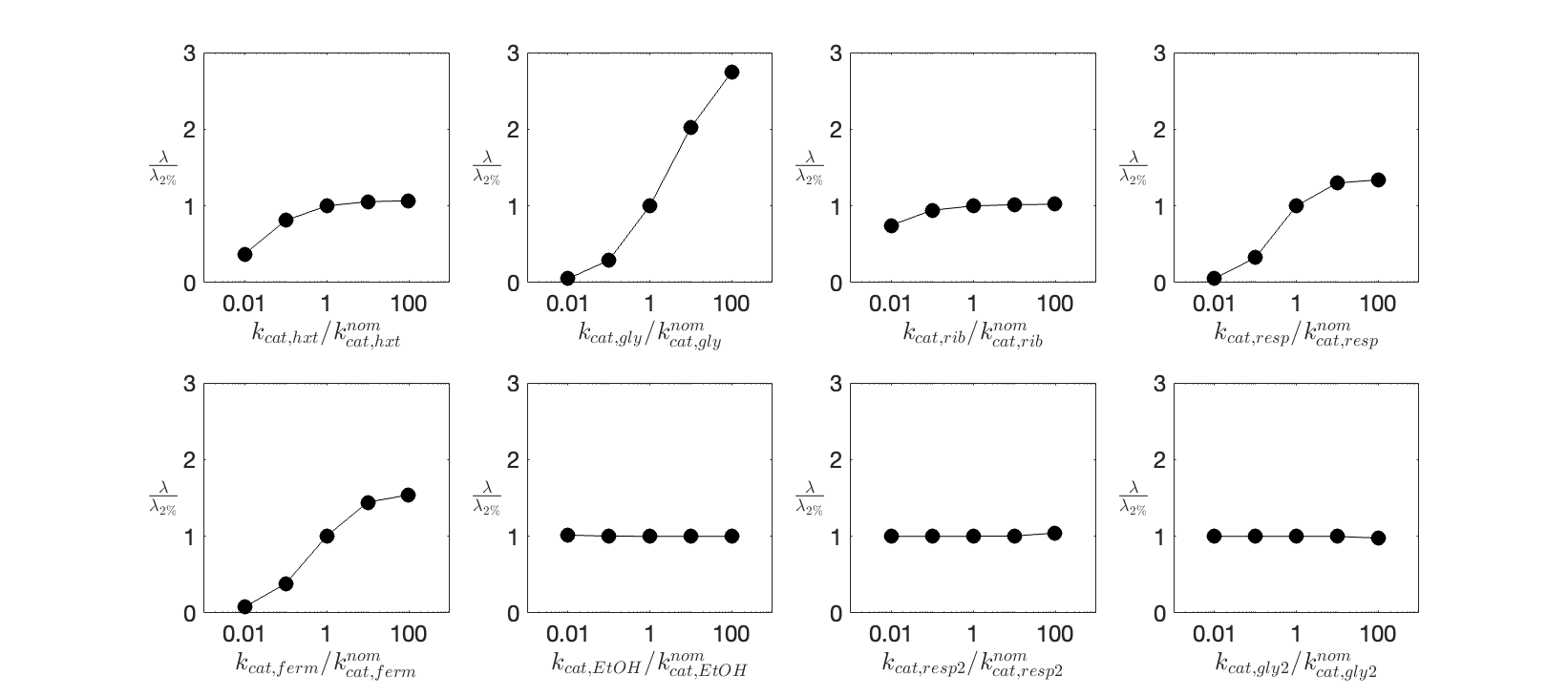


**Fig G. Sensitivity analysis of the *MeGro-2* output *λ* with respect to *k_cat_* parameters** Relative ratios of *λ* (normalized w.r.t. the values in 2% glucose) as a function the coefficient of variation of *k_cat,x_*, *x ∈ Prot2.*

A sensitivity analysis of *iMeGroCy-2* populations has been performed by changing a single parameter at a time in *GroCy-2* while keeping the remaining model parameters at their nominal value. This analysis links the *GroCy-2* model parameters to the selected quantitative features of the cell population. In particular, each parameter has been moved "forward" and "backward" with respect to its nominal value set for 2% glucose. The aim is to find out in which direction the population outputs move, once a given model parameter is “sufficiently” increased or decreased. The meaning of “sufficiently” is as follows. The goal is to vary the model parameter in order to have a significant and coherent modification of the output: for instance, if we increase a model parameter by factors 2x and 4x, do we obtain a variation of the output with the same sign for the two sizes of the parameter alteration and, at least for one of them, greater than, say, 5%? If the output modifications are smaller than 5%, does it help to increase the model parameter by higher factors, as 20x and 40x? How far can we go to have a significant output modification? Clearly, we cannot go too far because numerical problems could arise, or simply the range of the model parameter could go beyond the biological meaning (e.g., the growth parameters cannot be moved to the extent the straightforward growth rate becomes too low or too high). In other words, not all parameter variations are fixed to the same extent: they are “sufficiently” varied in order to show a significant output variation (or do not show a significant variation because they have been moved towards their bounds of meaningful values).

For each parameter setting (resulting from each parameter variation) of the sensitivity analysis, the exponential growth of a yeast population has been simulated (up to ~50,000 cells). The population features extracted from the virtual population are related to the protein distribution and relevant temporal features (such as the average value, standard deviation and coefficient of variation of the protein content, the MDT and the average values of the G_1_ periods of daughter, <T_G1D_>, and parent, <T_G1P_>, cells). Within this analysis, the model showed to be more sensitive to a set of parameters, rather than to others. For instance, *iMeGroCy-2* shows a dramatic dependence on parameters *K_2_*, *τ_2_* and *ρ*, parameters strictly related to the growth rate *λ* (according to Eq. (S27)). So, in order to keep the growth rate within biologically meaningful intervals, the sensitivity analysis for these parameters was reduced within a narrow interval centered on its corresponding nominal value, which was varied of (1/1.1)x, (1/1.25)x, 1.1x and 1.25x. For example, in Fig H, panel E is reported how the protein distribution, the average protein content and G_1_ period vary because of the aforementioned *ρ* variations. In this case it is apparent that we do have significant and coherent variations. To increase/decrease these values beyond the scalar factors reported would provide a growth rate too far from an acceptable qualitative behavior: for instance, it could even become negative according to Eq. (S27). Sensitivity analysis performed on the rate constant of ribosome synthesis, *K_1_* and on the time constant of ribosome degradation, *τ_1_*, shows very little variations when *K_1_* increases (10x and 100x), and pronounced variations when *K_1_* decreases (0.1x and 0.01x; see Fig H, Panel A), whilst the *iMeGroCy-2* outcomes seem to be minimally sensible to *τ_1_* (see Fig H, panel B).

Dealing with the sizer parameters, the results obtained by changing *Far1*(0) reveal that *iMeGroCy*-2 is substantially insensitive to the initial amount of Far1. In the simulations, the initial *Far1* has been varied from the nominal value (240 molecules, for 2% glucose) of ±50, ±100 molecules. As shown in Fig I, panel A, no significant variations are present in the protein content of the populations or in the length of G_1_ phase, even in the presence of a substantial variation (±100) of *Far1*(0). Similarly, no apparent variations are found by varying the clearance rate values of Far1, namely *η* and *n_F_* (Fig J, panels I and J). Concerning the other parameters *θ*, *k_on_*, *k_off_*, *k_cn_*, *k_nc_*, *h*, *H,* the sensitivity analysis for each parameter has been performed multiplying the corresponding nominal value by factors (1/5)x, (1/10)x, 5x and 10x. Results are resumed in Figs I and J.

Regards to timer *T_1b_* parameters *W_0_*, *W_1_*, *T_1min_* of Eq.(S30), we modified their nominal values according to the following variations: i) ±2, ±4, ±8 min for *W_0_*; ii) (1/1.001)x, (1/1.002)x, (1/1.005)x, 1.001x, 1.002x and 1.005x for *W_1_*; iii) (1/2)x, (1/4)x, (1/8)x, 2x, 4x and 8x for *T_1min_*. The very low variations used for *W_1_* are motivated by the marked dependence of the timer *T_1b_* on the value of *W_1_*: a too high value would provide a useless sizer, since *T_1b_* would only depend on *T_1bmin_*; a too low value would make *T_1b_* dependent only on *W_0_*. As it is evident from Fig K, panels B and C, we obtain opposite effects on the protein contents of the populations by varying *W_0_* or *W_1_* in the same direction. In particular, <*P*>, *P_0_*, *P_s_*, *P_cd_* increase, as well as the protein distribution shifts towards higher protein values, when *W_0_* increases, while they decrease when *W_1_* increases. Obviously, we can state that *iMeGroCy-2* is much more sensitive to variations of *W_1_* based on the low coefficients exploited in the sensitivity analysis of *W_1_*. No significant variations are obtained in the length of *T_G1_* by varying *W_0_* or *W_1_*. On the other hand, *iMeGroCy-2* shows a sensible increasing in the protein contents and in the length of *T_G1_* when *T_1min_* increases, see Fig K, panel A. Variations of the timer *T_2_* have been considered in the range (*T_2_^nom^*– 6 min, *T_2_^nom^*+ 6 min), with a time step of 2 minutes. By increasing/decreasing *T_2_* we obtain an overall increase/decrease of the protein content, because of the increase/decrease of the cell cycle length, and an increase in *T_G1_* converging to the same length for parent and daughter cells, Fig K, panel D. Similarly to *T_2_*, variations of timer *T_B_* have been considered in the range (*T_B_^nom^*– 5 min, *T_B_^nom^*+ 5 min), with a time step of 2 minutes. This time, by increasing (or decreasing) the length of the budded phase, we have an overall increase (or decrease) of the protein content, because buds have a longer (or shorter) period to grow. We have light variations (in the shape and in the position) of the protein distribution for values of *T_B_* lower than a critical value (in between 86 and 88 min) according to which, by further increasing *T_B_* we have a substantial change of the shape, as well as a decisive shift in the average value of the protein distribution, see Fig K, panel E. Moreover, in such critical cases we have a substantial decrease in the G_1_-phase variation. This outcome on the G_1_ phase suggests a situation with daughter and parent cells sharing the same shape of the protein distribution since both populations share the same G_1_ and budded period.





**Fig H. Sensitivity analysis with respect to *GroCy-2* parameters related to growth**. **Left panels:** simulated protein distributions drawn for different variations of the selected model parameter, w.r.t. the nominal value of 2% glucose (compared also to the experimental distribution, grey ghost). **Central panels:** protein features (average and initial cell protein content, critical cell size and size at division) extracted from the simulated populations, endowed with their standard deviations. **Right panels:** G_1_ phase length for the whole population and for the subpopulations of daughters and parents, endowed with their standard deviations.





**Fig I. Sensitivity analysis with respect to the parameters of the molecular trigger (part I)**. **Left panels:** simulated protein distributions drawn for different variations of the selected model parameter, w.r.t. the nominal value of 2% glucose (compared also to the experimental distribution, grey ghost). **Central panels:** protein features (average and initial cell protein content, critical cell size and size at division) extracted from the simulated populations, endowed with their standard deviations. **Right panels:** G_1_ phase length for the whole population and for the subpopulations of daughters and parents, endowed with their standard deviations.





**Fig J. Sensitivity analysis with respect to the parameters of the molecular trigger (part II)**. **Left panels:** simulated protein distributions drawn for different variations of the selected model parameter, w.r.t. the nominal value of 2% glucose (compared also to the experimental distribution, grey ghost). **Central panels:** protein features (average and initial cell protein content, critical cell size and size at division) extracted from the simulated populations, endowed with their standard deviations. **Right panels:** G_1_ phase length for the whole population and for the subpopulations of daughters and parents, endowed with their standard deviations.

**

**

**Fig K. Sensitivity analysis with respect to the parameters of the timer sub-model**. **Left panels:** simulated protein distributions drawn for different variations of the selected model parameter, w.r.t. the nominal value of 2% glucose (compared also to the experimental distribution, grey ghost). **Central panels:** protein features (average and initial cell protein content, critical cell size and size at division) extracted from the simulated populations, endowed with their standard deviations. **Right panels:** G_1_ phase length for the whole population and for the subpopulations of daughters and parents, endowed with their standard deviations.

All these data are collected in the heat map of Fig 3D, providing a qualitative/quantitative resume of the sensitivity analysis. The “quality” is provided by the color: ‘grey’ stand for substantial no output variation, ‘red’ stands for substantial increase and ‘blue’ stands for substantial decrease. The “quantity” is provided by the color strength, ranging from 10% up to 100% variation (or even more).

Similarly, we carried out a sensitivity analysis by varying the population parameters provided by the Coefficient of Variation of Timer length and protein/ribosome partitioning at division. Each CV has been increased (x2, x3) and reduced (divided by 2 and by 3). Results are reported in the following heatmap (Fig L) resembling the larger one reported in Fig 3D.


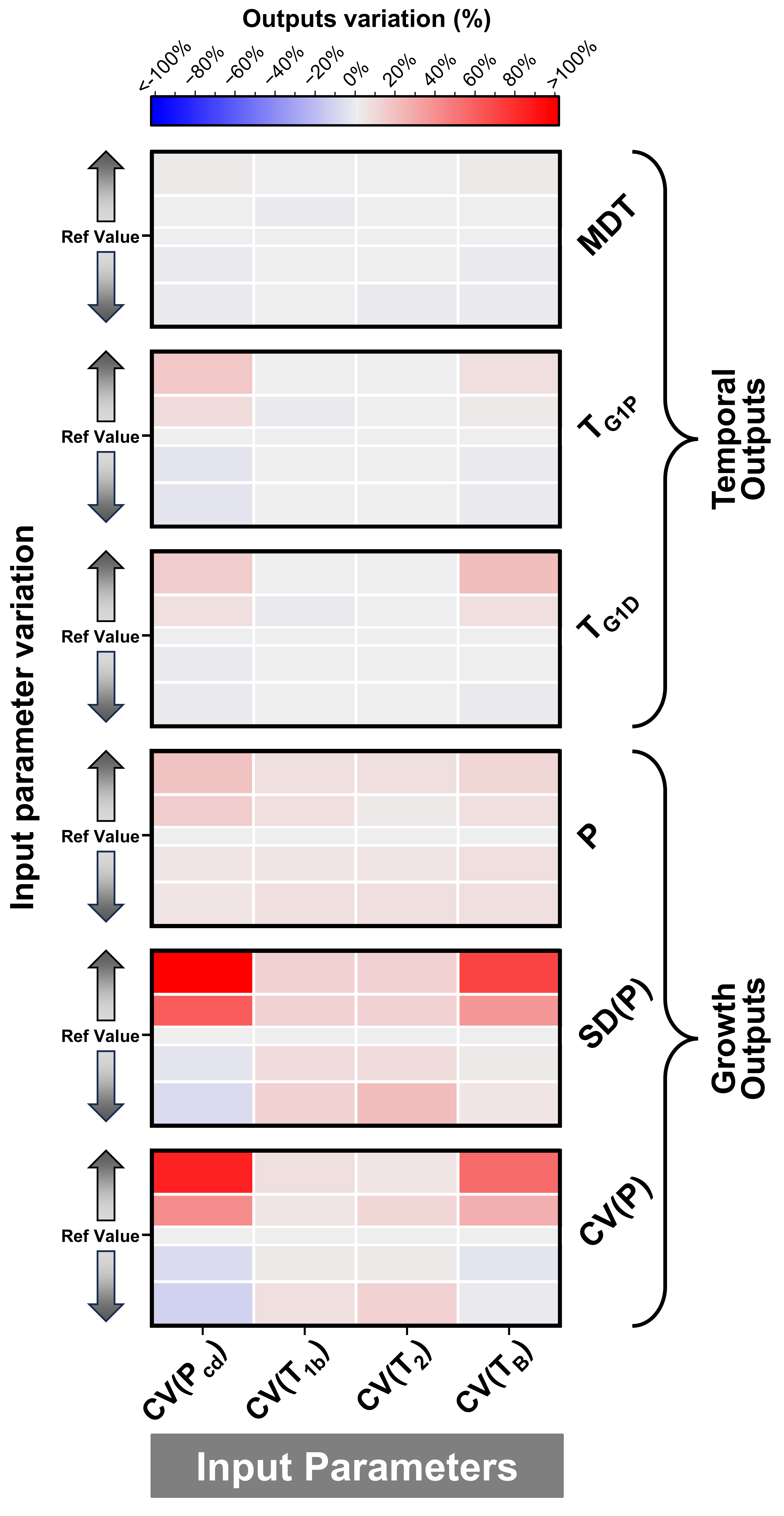


**Fig L. Alterations in major output growth and temporal parameters obtained by changing the GroCy-2 population parameters.** The considered temporal parameters are the population mass duplication time (MDT) and the G1 phase lengths of parent (TG1Par) and daughter cells (TG1Dau). The growth parameters are the average (P), standard deviation (SD(P)) and coefficient of variation (CV(P)) of the cellular protein content.

**5.e – Multi-parametric sensitivity analysis**

To further investigate the robustness of the simulations with respect to a selected set of parameter values, we analyzed what occurs by systematically and simultaneously varying a small set of GroCy parameters. A thorough analysis would involve in a systematic way the whole parameter space, i.e. a set of 20 parameters. Even by assuming a minimal set of 3 values for each (namely, the nominal value, a proper increase and a proper decrease) we would have 3^20^ (i.e. more than 3 billion) cases. As a matter of fact, such an investigation of the model robustness would have gone out of the scope of the manuscript, therefore we resorted in investigating what happens by varying four sets of selected 5 input parameters. Each set includes parameters from the growth module, the molecular interplay between Cln3 and Far1 and the Timer setting. More in details:

- Set 1: K_1_ (*10, /10), τ_1_ (*10, /10), k_on_ (*5, /5), W_1_ (*1.001, /1.001), T_2_ (*1.2, /1.2)
- Set 2: ρ (*1.1, /1.1), τ_2_ (*1.1, /1.1), k_off_ (*5, /5), W_1_ (*1.001, /1.001), T_2_ (*1.2, /1.2)
- Set 3: K_2_ (*1.1, /1.1), τ_1_ (*1.1, /1.1), k_nc_ (*5, /5), W_0_ (*1.001, /1.001), T_2_ (*1.2, /1.2)
- Set 4: K_1_ (*10, /10), ρ (*1.1, /1.1), Far1(0) (*1.2, /1.2), W_1_ (*1.001, /1.001), T_2_ (*1.2, /1.2)

Each parameter is allowed to vary up and down of the same average amount chosen for the single parameter sensitivity map reported in Fig 3D. That means, each set of 5-tuple provides 3^5^ = 243 populations (including the one without any parameter variation). For each parameter set we computed the same six outputs exploited to build the sensitivity heatmap, i.e. the average protein content with its standard deviation and coefficient of variation, the Mass Duplication Time, the average G1 phase for both Daughters and Parents. All these simulations refer to the glucose 2% nutritional environment.

Due to the importance of protein distribution as a fingerprint for each growth condition, Fig M illustrates the average protein content and its standard deviation for each simulation on an XY plane, where each point corresponds to a different simulation, color-coded according to the 5-tuple set. This graph offers a partial yet comprehensive representation of the entire set of simulations. The simulation results provided by the four 5-tuples clusters in two regions. Most simulations (approximately 80%) form a tight cluster with relatively low average protein content and standard deviation (Cluster Low), while a less populated cluster (Cluster High, about 20%) displays significantly higher values for both outputs.





**Fig M. Clustering of virtual populations as coming from multi-parametric sensitivity analysis**. Each point refers to a population. Different sets are reported according to different colors. Besides, the 7 simulated nutritional environments are reported, as well as 4 mutants discussed later.

**Cluster Low** encompasses most nutritional conditions, except for the two poorest ones, i.e., 0.05% glucose and 2% ethanol, which present an even smaller protein content position: the protein content range spans from approximately 3 to 6x10^10^ (with the nominal protein content being around 4.5x10^10^). Table H reports, for completeness, the set of multiparametric variations providing the different nutritional conditions and some mutants discussed later. No such variations involve a significant increase in *ρ* or in *K_2_* (indeed, all these populations are in Cluster Low, or in lower positions). We recall that the different nutritional conditions in Cluster Low have largely been addressed automatically by the model without the action of the modeler, because of the interplay between *MeGro-2* and *GroCy*, the former automatically tuning the growth parameters (*ρ, K_2_*) fed to the latter, according to its embedded optimization program. By getting poorer and poorer nutritional conditions, the *MeGro-2*/*GroCy* automatic adjustments are no longer sufficient, see Subsection 5.b for details.

**Table H. Set of multiparametric variations providing *in silico* different nutritional conditions and mutants**

|  | ρ | K_2_ | Far1(0) | k_on_ | H | Θ | W_0_ | W_1_ | T_2_ | T_B_ |
| --- | --- | --- | --- | --- | --- | --- | --- | --- | --- | --- |
| Glc 5% | *1.0005 | *1.0119 | - | - | - | - | - | - | - | - |
| Glc 0.5% | /1.0043 | /1.0473 | - | - | - | - | - | - | - | - |
| Glc 0.2% | /1.0166 | /1.1063 | - | - | - | - | - | - | - | *1.0588 |
| Glc 0.1% | /1.0342 | /1.1593 | - | - | - | - | - | - | - | *1.0588 |
| Glc 0.05% | /1.0611 | /1.2088 | /1.4118 | - | - | - | /1.0047 | - | - | *1.0588 |
| EtOH 2% | /2.5027 | *1.4327 | /2.1818 | - | /1.1472 | /1.1353 | /1.0252 | - | *3.5 | *1.5647 |
| TM6* | /1.0525 | /1.2252 | - | - | - | - | - | - | - | /1.0759 |
| *hxk2 hxk1* | /1.0493 | /1.3732 | - | - | - | - | - | - | - | *1.1765 |
| *rsa1* | /1.4408 | - | - | *2 | - | - | - | *1.0032 | /2.5 | *1.1765 |
| *whi5* | - | - | - | - | - | - | - | *1.0121 | - | - |

To obtain further details about populations in **Cluster Low**, we analyzed the proximity to the nominal Glucose 2% population based on a distance that considers all six outputs reported in the heatmap. To this end, we utilized the *L_∞_* norm, which provides the maximum percentage displacement among the six outputs. We found 67 populations that have a distance smaller than 5% from the reference population grown in 2% glucose and thus can be considered approximately equivalent to the nominal case.

Among these 67 populations:

- 19 are obtained by varying just 1 parameter. These cases come straightforward from the sensitivity heatmap where we investigated what happened by varying one parameter at a time
- 27 are obtained by varying 2 parameters
- 17 are obtained by varying 3 parameters
- 4 are obtained by varying 4 parameters
- No populations are obtained by varying all 5 parameters

Within these 67 simulated populations, we identified those with protein distributions closest to the nominal one by evaluating the Mean Square Error (MSE) between each distribution and the nominal one. The results show that the closest populations (MSE<50) are:

1. Set 1, 2 varied parameters (τ_1_*10, W_1_*1.001)
2. Set 1, 2 varied parameters (τ_1_/10, k_on_*5)
3. Set 1, 3 varied parameters (τ_1_/10, k_on_*5, W_1_*0.999)
4. Set 2, just 1 varied parameter (k_off_/5)
5. Set 2, 2 varied parameters (τ_2_*0.9091, k_off_*5)
6. Set 2, 3 varied parameters (τ_2_*0.9091, k_off_*5, W_1_*0.999)

Whilst the less close (among the closest, MSE>150) are

1. Set 1, 4 varied parameters (K_1_*10, τ_1_*10, k_on_/5, W_1_*1.001)
2. Set 2, 3 varied parameters (τ_2_*1.1, k_off_*5, W_1_*1.001)
3. Set 3, 2 varied parameters (k_nc_/5, W_0_*0.999)
4. Set 3, 2 varied parameters (τ_1_*1.1, W_0_*0.999)
5. Set 3, 3 varied parameters (τ_1_*1.1, k_nc_/5, W_0_*0.999)

Fig N below presents three of the best protein distributions (items 2, 4, 5) and one of the least accurate ones (item 10) in comparison to the nominal case (light cyan shadow). While the three best protein distributions (red, blue, magenta lines) are indeed superimposable with the nominal case, the less closely simulated population (green line) exhibits a protein distribution that starts to separate significantly from the nominal case. Therefore, this particular case provides a reference context that may help in judging the extent to which a specific data set captures the experimental protein distribution.


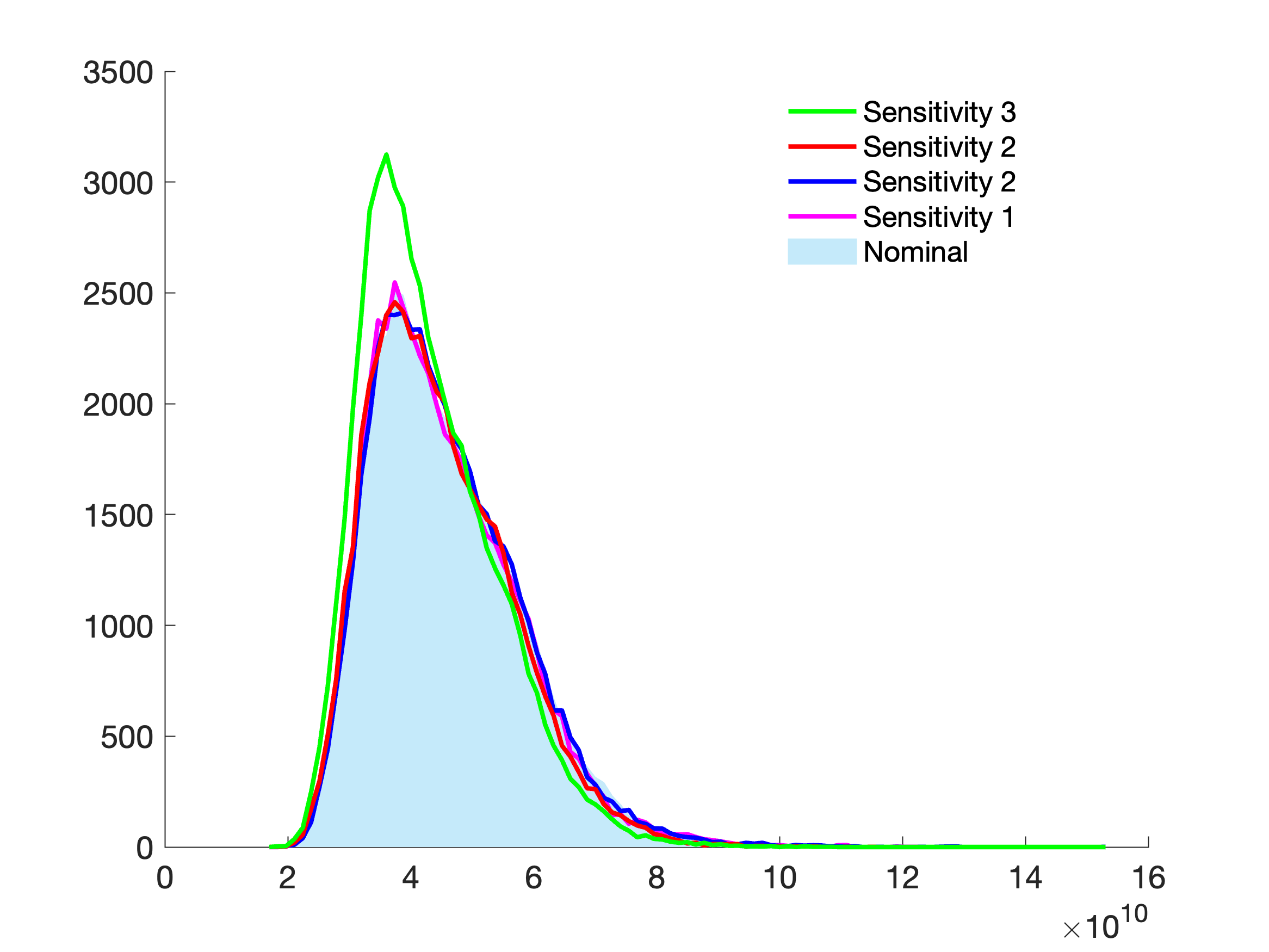


Item 10 (Set 3)

Item 5 (Set 2)

Item 4 (Set2)

Item 2 (Set 1)

Nominal

**Fig N. Protein distributions from Cluster Low.** Comparison of the protein distributions of 4 simulated populations among the 67 ones that have a distance (*L_∞_* norm) smaller than 5% from the reference population (nominal parameters used for WT 2% glucose) compared to the nominal one: red, blue, magenta distributions have a MSE < 50 while the green one has a MSE > 150 w.r.t. the nominal distribution.

Even the smallest average protein values of simulations of **Cluster High** are almost 70% larger than the 5% conditions. Literature data [13,14] indicate that *large* mutants have a size (mean or median volume) that is about twice that of the wild type, likely setting an upper limit for haploid cells. Since the mean volume of diploid cells is about 70%-100% larger than haploid cells [11,12], and cells with higher ploidy (at least up to 4n) roughly increase their size proportionality, populations with an average protein content larger than 10^11^ might represent cells with a diploid DNA content or higher. It is worth noting that achieving an average protein distribution in the **Cluster High** relies significantly on the parameters *ρ* and *K_2_*, whose slight modifications provide considerable variations of the chosen outputs: indeed (i) Set 1 (which does not change *ρ* and *K_2_*) does not exhibit any such case, while (ii) Sets 2-4, which provide populations in **Cluster High**, all demonstrate an increase in *ρ* or *K_2_*. The following Table I reports the kind of parameter variations and the number of simulated populations belonging to the **Cluster High**.

**Table I. Set of multiparametric variations providing *in silico* populations in Cluster High**

| **Simulation set** | **Parameter change** | | | | | | |
| --- | --- | --- | --- | --- | --- | --- | --- |
|  | ρ | τ_2_ | k_off_ | W_1_ | | | T_2_ |
| Set 2: 81 populations | *1.1 | any | any | any | | | any |
|  | | | | | | | |
|  | K_2_ | τ_1_ | k_nc_ | | W_0_ | T_2_ | |
| Set 3: 81 populations | *1.1 | any | any | | any | any | |
|  | | | | | | | |
|  | K_1_ | ρ | Far1(0) | | W_1_ | T_2_ | |
| Set 4: 27 populations | - | *1.1 | any | | any | any | |
| Set 4: 27 populations | *10 | *1.1 | any | | any | any | |

**5.f - Simulations of cell populations through *Hy-iMeGroCy-2***

To illustrate our "whole-cell modeling" concept, we used the *iMeGroCy-2* model as a scaffold for finer molecular plug-ins. To this end, a molecular model of the G_1_/S transition is plugged into the *iMeGroCy-2* model to replace the timers describing the G_1_ phase (i.e., T_1_ and T_2_; Fig 8D). The adopted *G1_S module* [15] describes in detail the molecular mechanisms leading to the activation of the G_1_/S regulon. Similarly to the molecular trigger mechanism of *GroCy-2*, the *G1_S module* links cell growth and molecular events promoting the G_1_/S transition by means of cyclin Cln3 dynamics, whose total amount is proportional to the overall protein content. Cln3 activity is still delayed by its inhibitor Far1. Further molecular events accounted for in the *G1_S module* include regulation of Cln3-Cdk1 activity and transcriptional activation of the G_1_/S regulon - driven by SBF and MBF and inhibited by Whi5. The activation of the G_1_/S regulon leads to the synthesis of Cln1, Cln2, Clb5, Clb6, and Nrm1, which play a direct role in later molecular events. The model includes cytoplasm (cyt), endoplasmic reticulum (ER), and nucleus (nuc) sub-cellular compartments. The G_1_/S regulon activation depends on multisite phosphorylation of Whi5, SBF, and MBF catalyzed by Cln-Cdk1 complexes. Dealing with the timers, according to the *G1_S module*, the *T_1_* period lasts from birth up to when 50% of Whi5 has left the nucleus to the end of the multisite phosphorylation period; then, the *T_2_* period starts and is taken to end when 50% of Sic1 – the inhibitor of the Clb-Cdk1 complexes - has left the nucleus following phosphorylation by Cln1,2,3- and Clb5,6-cdk1 complexes. From an input/output behavior the *Hy-iMeGroCy-2* works as follows. *MeGro-2* is still the master module that exploits the nutritional environment and provides the growth parameters *ρ* and *K_2_* to the Growth module, which is now partially replaced by a larger set of ODE yet requiring the pair (*ρ*, *K_2_*) as input parameters (indeed, the growth equations of the molecular *G1_S module* are the same of *GroCy-2*). The *G1_S module* works providing the lengths of the 3 Timers *T_1a_*, *T_1b_* and *T_2_* according to a detailed molecular machinery: the end of *T_1a_* (that coincides with the onset of *T_1b_*) is still constrained to the time instant when free nuclear Cln3 gets rid of inhibitor Far1 (although according to slightly more complicated machinery involving also chaperon Ydj1); the end of *T_1b_* (that coincides with the onset of *T_2_*) is now related to the time instant when 50% of Whi5 has left the nucleus (rather than to a coarse relationship with the size of the cell), and the end of *T_2_* (that coincides with the onset of the budded phase) is now related to the time instant when 50% of Sic1 has left the nucleus. *T_B_* is set by the modeler, similarly to *iMeGroCy-2*.

The *G1_S module* in [15] simulates and predicts single daughter cells or sets of independent daughter cells: its exploitation to make population simulations of daughter and parent cells of different genealogical ages, is a novelty of the present manuscript, as well as the tuning of its parameters to reproduce different glucose environments.

*G1_S module* simulations in [15] refer to cells growing in high glucose. The related parameter values have been considered the starting point for the glucose 2% case, aiming to make *Hy-iMeGroCy-2* population simulations consistent with *iMeGroCy-2* population simulations. Only a few *G1_S module* parameters had to be slightly modified from our previous work [15]. The *G1_S module* parameters tuning procedure aimed at reproducing timers and protein contents provided by *iMeGroCy-2* cell chains. The use of small cell chains instead of populations has been considered a good compromise between accuracy and computational speed because a trial and error approach would not be affordable for populations of thousands of cells involving the *G1_S module* (see [15] for the computational burden associated to the *G1_S module*).

The *G1_S module* parameters that have the same meaning as the corresponding ones in *GroCy-2* (for instance, Cln3 and Far1 binding/unbinding coefficients or the initial amount of Far1) are set accordingly to *iMeGroCy-2*. Other *G1_S module* parameter values that do not vary with the genealogical age are taken from [15]. Table J reports the parameter values that are supposed to vary according to the type of cell (Daughter, Parent and genealogical age) and to the quality of the glucose nutrient. In general, the smallest number of parameters have been changed compared to [15], in keeping with the notion that cell cycle parameters are likely to change significantly only at the lowest glucose concentrations. In detail:

• *k_14_* (the binding coefficient of the reaction Swi6Swi4 + Whi5 --> Swi6Swi4Whi5) increases, or at least remains constant when the glucose concentration decreases; it is set to higher values in daughter cells rather than in parent cells; it decreases, or at most it remains constant, by increasing the genealogical age;

• *k_21_* (the diffusion coefficient of Whi5 from the nucleus into the cytoplasm) and *k_21p_* (the diffusion coefficient of phosphorylated Whi5 from the nucleus into the cytoplasm) decrease, or at most remain constant when the glucose concentration decreases; they are set to lower values in daughter cells rather than in parent cells; they increase, or at least remain constant, by increasing the genealogical age;

• *k_31_* (the binding coefficient of the reaction Clb6 + Sic1 --> Clb6Sic1), *k_33_* (the binding coefficient of the reaction Clb6 + Sic1p --> Clb6Sic1p), *k_37_* (the binding coefficient of the reaction Clb5 + Sic1 --> Clb5Sic1), *k_39_* (the binding coefficient of the reaction Clb5 + Sic1p --> Clb5Sic1p) increase, or at least they remain constant, when the glucose concentration decreases; they are set to higher values in daughter cells rather than in parent cells; they decrease, or at most they remain constant, by increasing the genealogical age;

• *k_43_* (the diffusion coefficient of Sic1p out of the nucleus) decreases, or at most, it remains constant when the glucose concentration decreases; it is set to lower values in daughter cells rather than in parent cells; it increases, or at least it remains constant, by increasing the genealogical age;

• *Sic1_tot_* (the initial amount of Sic1) has a lower value for parent cells than daughter cells.

Fig O compares protein distributions for the *Hy-iMeGroCy-2* to the corresponding experimental ones for different glucose environments. Table K reports outputs for the *Hy-iMeGroCy-2* population simulations.


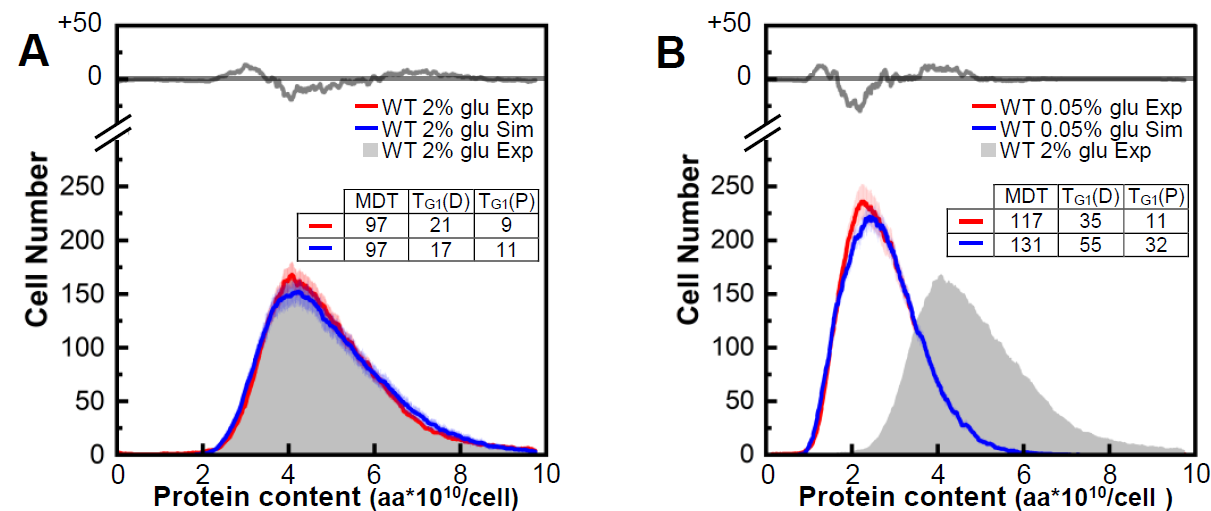


**Fig O.** **The *iMeGroCy-2* coarse-grained model acts as a scaffold for a molecularly detailed module of the G1/S transition.** Protein distributions predicted by *Hy-iMeGroCy-2* model (blue lines), compared to the related experimental distributions (red lines), for wild-type strains grown in 2% (A) and 0.05% (B) glucose media. Mean ± SD values are shown. In each panel, the gray line in the upper insert represents the difference between the experimental and simulated values. The experimental distribution of wild-type cells cultivated in 2% glucose (filled gray) is also reported as a reference. Lower inserts show the temporal parameters for both experimental and simulated populations.

**Table J.** ***G1_S* *module* input parameters**

| **Parameters** | **Meas. unit** | **glc 0.05%** | **glc 0.2%** | **glc 2%** | **glc 5%** |
| --- | --- | --- | --- | --- | --- |
| ***k_14_(D_1_)*** | (molec/L)^-1^min^-1^ | 5.60e-14 | 4.48e-14 | 1.60e-14 | 1.57e-14 |
| ***k_14_(D_2_)*** | (molec/L)^-1^min^-1^ | 5.28e-14 | 4.32e-14 | 7.12e-15 | 5.28e-15 |
| ***k_14_(D_3_)*** | (molec/L)^-1^min^-1^ | 5.12e-14 | 2.72e-14 | 3.14e-15 | 2.32e-15 |
| ***k_14_(D_4_)*** | (molec/L)^-1^min^-1^ | 5.12e-14 | 2.4e-14 | 3.14e-15 | 2.03e-15 |
| ***k_14_(D_5_)*** | (molec/L)^-1^min^-1^ | 4.96e-14 | 2.4e-14 | 2.85e-15 | 1.92e-15 |
| ***k_14_(D_6_)*** | (molec/L)^-1^min^-1^ | 4.96e-14 | 2.4e-14 | 2.70e-15 | 1.84e-15 |
| ***k_14_(D_k_), k > 6*** | (molec/L)^-1^min^-1^ | 4.96e-14 | 2.4e-14 | 2.67e-15 | 1.79e-15 |
| ***k_14_(P_k_), k ≥ 1*** | (molec/L)^-1^min^-1^ | 3.20e-14 | 1.68e-15 | 1.68e-15 | 1.68e-15 |
| ***k_21_(D_1_)*** | min^-1^ | 0.0024 | 0.0062 | 0.11 | 0.31 |
| ***k_21_(D_2_)*** | min^-1^ | 0.0030 | 0.0105 | 2.80 | 4.76 |
| ***k_21_(D_3_)*** | min^-1^ | 0.0033 | 0.0136 | 3.50 | 4.76 |
| ***k_21_(D_4_)*** | min^-1^ | 0.0034 | 0.0145 | 4.20 | 5.18 |
| ***k_21_(D_5_)*** | min^-1^ | 0.0035 | 0.0151 | 4.20 | 5.60 |
| ***k_21_(D_6_)*** | min^-1^ | 0.0035 | 0.0153 | 4.20 | 5.74 |
| ***k_21_(D_k_), k > 6*** | min^-1^ | 0.0035 | 0.0153 | 4.20 | 5.74 |
| ***k_21_(P_1_)*** | min^-1^ | 0.0046 | 0.0336 | 6.02 | 6.02 |
| ***k_21_(P_2_)*** | min^-1^ | 0.0100 | 2.03 | 6.02 | 6.02 |
| ***k_21_(P_3_)*** | min^-1^ | 0.0161 | 4.48 | 6.02 | 6.02 |
| ***k_21_(P_4_)*** | min^-1^ | 0.0196 | 5.53 | 6.02 | 6.02 |
| ***k_21_(P_5_)*** | min^-1^ | 0.0210 | 6.02 | 6.02 | 6.02 |
| ***k_21_(P_6_)*** | min^-1^ | 0.0210 | 6.02 | 6.02 | 6.02 |
| ***k_21_(P_k_), k > 6*** | min^-1^ | 0.0210 | 6.02 | 6.02 | 6.02 |
| ***k_21p_(D_1_)*** | min^-1^ | 0.024 | 0.062 | 1.1 | 3.08 |
| ***k_21p_(D_2_)*** | min^-1^ | 0.030 | 0.105 | 28 | 47.6 |
| ***k_21p_(D_3_)*** | min^-1^ | 0.033 | 0.136 | 35 | 47.6 |
| ***k_21p_(D_4_)*** | min^-1^ | 0.034 | 0.145 | 42 | 51.8 |
| ***k_21p_(D_5_)*** | min^-1^ | 0.035 | 0.151 | 42 | 56.0 |
| ***k_21p_(D_6_)*** | min^-1^ | 0.035 | 0.153 | 42 | 57.4 |
| ***k_21p_(D_k_), k > 6*** | min^-1^ | 0.035 | 0.153 | 42 | 57.4 |
| ***k_21p_(P_1_)*** | min^-1^ | 0.046 | 0.336 | 60.2 | 60.2 |
| ***k_21p_(P_2_)*** | min^-1^ | 0.100 | 20.3 | 60.2 | 60.2 |
| ***k_21p_(P_3_)*** | min^-1^ | 0.161 | 44.8 | 60.2 | 60.2 |
| ***k_21p_(P_4_)*** | min^-1^ | 0.196 | 55.3 | 60.2 | 60.2 |
| ***k_21p_(P_5_)*** | min^-1^ | 0.210 | 60.2 | 60.2 | 60.2 |
| ***k_21p_(P_6_)*** | min^-1^ | 0.210 | 60.2 | 60.2 | 60.2 |
| ***k_21p_(P_k_), k > 6*** | min^-1^ | 0.210 | 60.2 | 60.2 | 6.02 |
| ***k_31_(D_1_)*** | min^-1^ | 2.88e-15 | 2.18e-15 | 1e-15 | 1e-15 |
| ***k_31_(D_2_)*** | min^-1^ | 2.3e-15 | 1.85e-15 | 1e-15 | 8e-16 |
| ***k_31_(D_3_)*** | min^-1^ | 2.23e-15 | 1.8e-15 | 8e-16 | 2e-16 |
| ***k_31_(D_4_)*** | min^-1^ | 2.21e-15 | 1.6e-15 | 3.5e-16 | 2e-16 |
| ***k_31_(D_5_)*** | min^-1^ | 2.21e-15 | 1.59e-15 | 2.5e-16 | 2e-16 |
| ***k_31_(D_6_)*** | min^-1^ | 2.1e-15 | 1.59e-15 | 2.09e-16 | 2e-16 |
| ***k_31_(D_k_), k > 6*** | min^-1^ | 2.08e-15 | 1.58e-15 | 2e-16 | 2e-16 |
| ***k_31_(P_1_)*** | min^-1^ | 2e-15 | 1.56e-15 | 2e-16 | 2e-16 |
| ***k_31_(P_2_)*** | min^-1^ | 2e-15 | 1.56e-15 | 2e-16 | 2e-16 |
| ***k_31_(P_k_), k > 2*** | min^-1^ | 2e-15 | 2e-16 | 2e-16 | 2e-16 |
| ***k_33_(D_1_)*** | min^-1^ | 2.88e-16 | 2.18e-16 | 1e-16 | 1e-16 |
| ***k_33_(D_2_)*** | min^-1^ | 2.3e-16 | 1.85e-16 | 1e-16 | 8e-17 |
| ***k_33_(D_3_)*** | min^-1^ | 2.23e-16 | 1.8e-16 | 8e-17 | 2e-17 |
| ***k_33_(D_4_)*** | min^-1^ | 2.21e-16 | 1.6e-16 | 3.5e-17 | 2e-17 |
| ***k_33_(D_5_)*** | min^-1^ | 2.21e-16 | 1.59e-16 | 2.5e-17 | 2e-17 |
| ***k_33_(D_6_)*** | min^-1^ | 2.1e-16 | 1.59e-16 | 2.09e-17 | 2e-17 |
| ***k_33_(D_k_), k > 6*** | min^-1^ | 2.08e-16 | 1.58e-16 | 2e-17 | 2e-17 |
| ***k_33_(P_1_)*** | min^-1^ | 2e-16 | 1.56e-16 | 2e-17 | 2e-17 |
| ***k_33_(P_2_)*** | min^-1^ | 2e-16 | 1.56e-16 | 2e-17 | 2e-17 |
| ***k_33_(P_k_), k > 2*** | min^-1^ | 2e-16 | 2e-17 | 2e-17 | 2e-17 |
| ***k_37_(D_1_)*** | min^-1^ | 2.88e-15 | 2.18e-15 | 1e-15 | 1e-15 |
| ***k_37_(D_2_)*** | min^-1^ | 2.3e-15 | 1.85e-15 | 1e-15 | 8e-16 |
| ***k_37_(D_3_)*** | min^-1^ | 2.23e-15 | 1.8e-15 | 8e-16 | 2e-16 |
| ***k_37_(D_4_)*** | min^-1^ | 2.21e-15 | 1.6e-15 | 3.5e-16 | 2e-16 |
| ***k_37_(D_5_)*** | min^-1^ | 2.21e-15 | 1.59e-15 | 2.5e-16 | 2e-16 |
| ***k_37_(D_6_)*** | min^-1^ | 2.1e-15 | 1.59e-15 | 2.09e-16 | 2e-16 |
| ***k_37_(D_k_), k > 6*** | min^-1^ | 2.08e-15 | 1.58e-15 | 2e-16 | 2e-16 |
| ***k_37_(P_1_)*** | min^-1^ | 2e-15 | 1.56e-15 | 2e-16 | 2e-16 |
| ***k_37_(P_2_)*** | min^-1^ | 2e-15 | 1.56e-15 | 2e-16 | 2e-16 |
| ***k_37_(P_k_), k > 2*** | min^-1^ | 2e-15 | 2e-16 | 2e-16 | 2e-16 |
| ***k_39_(D_1_)*** | min^-1^ | 2.88e-16 | 2.18e-16 | 1e-16 | 1e-16 |
| ***k_39_(D_2_)*** | min^-1^ | 2.3e-16 | 1.85e-16 | 1e-16 | 8e-17 |
| ***k_39_(D_3_)*** | min^-1^ | 2.23e-16 | 1.8e-16 | 8e-17 | 2e-17 |
| ***k_39_(D_4_)*** | min^-1^ | 2.21e-16 | 1.6e-16 | 3.5e-17 | 2e-17 |
| ***k_39_(D_5_)*** | min^-1^ | 2.21e-16 | 1.59e-16 | 2.5e-17 | 2e-17 |
| ***k_39_(D_6_)*** | min^-1^ | 2.1e-16 | 1.59e-16 | 2.09e-17 | 2e-17 |
| ***k_39_(D_k_), k > 6*** | min^-1^ | 2.08e-16 | 1.58e-16 | 2e-17 | 2e-17 |
| ***k_39_(P_1_)*** | min^-1^ | 2e-16 | 1.56e-16 | 2e-17 | 2e-17 |
| ***k_39_(P_2_)*** | min^-1^ | 2e-16 | 1.56e-16 | 2e-17 | 2e-17 |
| ***k_39_(P_k_), k > 2*** | min^-1^ | 2e-16 | 2e-17 | 2e-17 | 2e-17 |
| ***k_43_(D_1_)*** | min^-1^ | 0.0406 | 0.0692 | 0.308 | 0.413 |
| ***k_43_(D_2_)*** | min^-1^ | 0.0435 | 0.1 | 1.12 | 4.9 |
| ***k_43_(D_3_)*** | min^-1^ | 0.0462 | 0.1194 | 31.5 | 56 |
| ***k_43_(D_4_)*** | min^-1^ | 0.0473 | 0.1215 | 42 | 56 |
| ***k_43_(D_5_)*** | min^-1^ | 0.0476 | 0.1246 | 49 | 56 |
| ***k_43_(D_6_)*** | min^-1^ | 0.0476 | 0.1267 | 54.6 | 56 |
| ***k_43_(D_k_), k > 6*** | min^-1^ | 0.0476 | 0.1267 | 56 | 56 |
| ***k_43_(P_1_)*** | min^-1^ | 0.0512 | 0.1526 | 56 | 56 |
| ***k_43_(P_2_)*** | min^-1^ | 0.0945 | 1.12 | 56 | 56 |
| ***k_43_(P_3_)*** | min^-1^ | 0.1344 | 38.5 | 56 | 56 |
| ***k_43_(P_4_)*** | min^-1^ | 0.1540 | 56 | 56 | 56 |
| ***k_43_(P_5_)*** | min^-1^ | 0.1603 | 56 | 56 | 56 |
| ***k_43_(P_6_)*** | min^-1^ | 0.1603 | 56 | 56 | 56 |
| ***k_43_(P_k_), k > 6*** | min^-1^ | 0.1603 | 56 | 56 | 56 |
| ***Sic1_tot_(D_k_), k≥1*** | min^-1^ | 190 | 190 | 190 | 190 |
| ***Sic1_tot_(P_k_), k≥1*** | min^-1^ | 140 | 140 | 140 | 140 |

**Table K. *Hy-iMeGroCy-2* population output parameters. Light cyan rows refer to control experimental data**

| **Parameters** | **Meas. Unit** | **glc 0.05%** | **glc 0.2%** | **glc 2%** | **glc 5%** |
| --- | --- | --- | --- | --- | --- |
| ***MDT*** | Min | 130.8 | 110.9 | 97.4 | 96.3 |
| ***MDT*** | Min | 117 | 104 | 97 | 96 |
| **2^-^*^TD/MDT^* +**  **2^-^*^TP/MDT^*** | - | 0.989 | 0.990 | 0.989 | 0.986 |
| **2^-^*^TD/MDT^* +**  **2^-^*^TP/MDT^*** | - | 1.008 | 1.007 | 1.003 | 1.003 |
| ***T_G1_(D)*** | min | 54.5 ± 8.9 | 29.2 ± 4.7 | 16.6 ± 3.0 | 15.3 ± 2.8 |
| ***T_G1_(D)*** | min | 35 | 22 | 21 | 17 |
| ***T_G1_(P)*** | min | 31.8 ± 7.6 | 16.0 ± 4.1 | 11.2 ± 0.7 | 11.2 ± 0.7 |
| ***T_G1_(P)*** | min | 11 | 8 | 9 | 6 |
| ***T_B_*** | min | 90.0 ± 2.7 | 90.1 ± 2.7 | 85.1 ± 2.6 | 85.1 ± 2.6 |
| ***T_B_*** | min | 93 ± 2 | 88 ± 1 | 82 ± 2 | 84 ± 1 |
| ***T_D_*** | min | 144.5 ± 9.3 | 119.2 ± 5.4 | 101.7 ± 4.0 | 100.4 ± 3.8 |
| ***T_D_*** | min | 128 ± 1 | 110 ± 0 | 103 ± 1 | 101 ± 0 |
| ***T_P_*** | min | 121.8 ± 8.1 | 106.1 ± 4.9 | 96.3 ± 2.7 | 96.3 ± 2.6 |
| ***T_P_*** | min | 104 ± 0 | 96 ± 0 | 91 ± 1 | 90 ± 0 |
| ***F_G1*_*** | % | 10.6 ± 0.9 | 9.8 ± 0.4 | 9.7 ± 0.7 | 9.6 ± 0.4 |
| ***<P>/<P>_2%_*** | - | 0.55 | 0.92 | 1.0 | 1.11 |
| ***<P>/<P>_2%_*** | - | 0.60 | 0.79 | 1.0 | 1.13 |

**6 - Simulations of mutant populations**

As previously hinted in Section 2, the model is conceived to predict the experimental behavior of mutants whenever the genetic alteration is known (or is supposed) to be related to the variation of a specific set of model parameters. This is the case of a pair of mutants defecting of the upper part of the glycolysis (*hxk2 hxk1*) and of the glucose transport (TM6*), with the model able to catch the qualitative behavior of the variations of the output population features by suitably varying a couple of *MeGro-2* module parameters. On the other hand, we played the opposite game according to a standard of reverse engineering: given a pair of mutants whose lab experiments provided specific growth and temporal variations with respect to the wild type case, we exploited the *iMeGroCy-2* sensitivity heat map of Fig 3D to understand what parameter variations could be responsible for it. Such a task has been performed on mutants of ribosome biogenesis (*rsa1*) and of cell cycle (*whi5Δ*, *whi5-4E*). This last mutant has been also simulated exploiting the molecular plug-in accounting for the required molecular details (*Hy-iMeGroCy-2* simulation). It is worth noticing that the reverse engineering approach can be exploited also without relying on any biological assumption for the mutant under investigation (as well as for any apparent output variations occurring because of an unknown genetic/environmental perturbation).

**6.a – Mutant *hxk2 hxk1***

Mutant *hxk2 hxk1* lacks two of the phosphorylating isoenzymes that catalyze the first glycolytic step [16,17]. To reproduce the *hxk2 hxk1* low efficiency of glycolysis, under the nutrient conditions of 2%, 0.1% glucose, we reduced the wild type catalytic coefficient of the fluxes $\upsilon_{gly}$ and $\upsilon_{gly2}$ (namely, $k_{cat,gly}$ and $k_{cat,gly2}$) by a factor of 1/2, which is roughly consistent with the reduction in the glucose kinase activity measured in this mutant strain, Fig S, panel J. No loss of efficiency is assumed in ethanol 2%. All other *iMeGroCy-2* parameters are set according to Tables C and E, except for *T*_B_ length, set at the experimental mutant values. The *hxk2 hxk1* input parameter variations and outputs are reported in Table L, and show the following variations w.r.t. the wild type *MeGro-2* outputs in the glucose environments reported inTable E as inputs to *GroCy-2*: a reduction of both the ribosome-over-protein ratio ρ (~3% for 0.1% and ~5% for 2%) and the exponential growth rate *λ* (~21% for 0.1% and ~32% for 2%), as well as a reduction of the protein synthesis rate *K_2_* (~17% for 0.1% and ~27% for 2%) for the glucose environments. Clearly, no alterations are expected in the ethanol environment, since no parameter variations are considered. These results are reported in Fig 7.

**6.b – Mutant TM6***

Mutant TM6* exhibits a strongly reduced sugar uptake capacity ([18]; Fig S, panel I). In order to model the TM6* low efficiency of glucose transport, we reduced the wild type catalytic coefficient of the *MeGro-2* flux $\upsilon_{hxt}$ (namely, $k_{cat,hxt}$) by a factor of 5. This parameter variation is consistent with direct *in vivo* measurements of the glucose uptake capacity for this mutant (Fig S, panel I); [18]). Besides, we increased the protein investment $\gamma_{hxt}$ for simulations in ethanol environment: as in a pure ethanol environment the flux through the transporter ‘hxt’ vanishes (see Subsection 3.b), the TM6* low efficiency of the glucose transport cannot be rendered by simply changing the *MeGro-2* parameter $k_{cat,hxt}$ as done in glucose, because the parameter variation would not produce any effect on the mutant growth in ethanol. Therefore, for the *MeGro-2* simulation of TM6* in ethanol we tested different fixed investments $\gamma_{hxt}$ from 0 to 0.1 to obtain the optimal experimental MDT, finding that a satisfactory setting can be obtained for the upper bound $\gamma_{hxt}$ = 0.1. All other *iMeGroCy-2* parameters are set according to Tables C and E, except for T_B_ length, set at the experimental mutant values. The *MeGro-2* outputs related to the modified parameter setting are reported in Table L and show the following variations w.r.t. the wild type *MeGro-2* outputs in the glucose environments reported in Table E as inputs to *GroCy-2*: (a) a reduction of both the ribosome-over-protein ratio ρ (~8% for 0.1% and ~5% for 2%) and the exponential growth rate *λ* (~18% for 0.1% and ~24% for 2%), as well as a reduction of the protein synthesis rate *K_2_* (~9% for 0.1% and ~18% for 2%), for case (i); (b) a reduction of both the ribosome-over-protein ratio *ρ* (~27%) and the exponential growth rate *λ* (~11%), and an increasing of *K_2_* (~22%), for case (ii); (c) an increase in the population parameter CV(P_cd_), i.e. the coefficient of variation of the protein/ribosome at division for the glucose nutritional environment. The results of the TM6* simulations are reported in Fig 7.

**Table L. *iMeGroCy-2* input/output parameter variations for mutants *hxk2 hxk1 and* TM6***

| **Parameters** | **TM6*** | | | ***hxk2 hxk1*** | | |
| --- | --- | --- | --- | --- | --- | --- |
|  | **EtOH 2%** | **glc 0.1%** | **glc 2%** | **EtOH 2%** | **glc 0.1%** | **glc 2%** |
|  | $\boldsymbol{F}\boldsymbol{=0}$ | $\boldsymbol{F}\boldsymbol{=}\mathbf{0.460}$ | $\boldsymbol{F}\boldsymbol{=}\mathbf{0.608}$ | $\boldsymbol{F}\boldsymbol{=0}$ | $\boldsymbol{F}\boldsymbol{=}\mathbf{0.242}$ | $\boldsymbol{F}\boldsymbol{=}\mathbf{0.511}$ |
|  | $\boldsymbol{\gamma}_{\boldsymbol{hxt}}\boldsymbol{=0.1}$ | **free invest.** | **free invest.** | $\boldsymbol{\gamma}_{\boldsymbol{hxt}}\boldsymbol{=0.01}$ | **free invest.** | **free invest.** |
|  | **k_cat,hxt_= k_cat,hxt_^nom^/5** | **k_cat,hxt_= k_cat,hxt_^nom^/5** | **k_cat,hxt_= k_cat,hxt_^nom^/5** | **No MeGro parameter variations** | **k_cat,gly_= k_cat,gly_^nom^/2,**  **k_cat,gly2_= k_cat,gly2_^nom^/2** | **k_cat,gly_= k_cat,gly_^nom^/2,**  **k_cat,gly2_= k_cat,gly2_^nom^/2** |
| ***ρ*** | 6.085e-06 | 1.850e-05 | 1.980e-05 | 8.327e-06 | 1.945e-05 | 1.986e-05 |
| ***K****_2_* | 645.14 | 287.51 | 300.10 | 526.80 | 263.66 | 267.76 |
| ***K****_2_^1^* | 611.37 | 265.11 | 274.45 | 493.82 | 243.82 | 246.90 |
| ***K****_2_^2^* | 345.70 | 143.84 | 146.98 | 274.77 | 132.89 | 133.96 |
| ***K****_2_^3^* | 168.59 | 62.99 | 62.00 | 128.74 | 58.93 | 58.67 |
| ***K****_2_^4^* | 139.08 | 49.52 | 47.84 | 104.40 | 46.60 | 46.12 |
| ***K****_2_^5^* | 124.32 | 42.78 | 40.76 | 92.23 | 40.44 | 39.85 |
| ***K****_2_^s^, s > 5* | 112.51 | 37.39 | 35.09 | 82.49 | 35.51 | 34.83 |
| ***T****_B_* | 146 | 87 | 79 | 137 | 103 | 100 |
| ***CV(P_cd_)*** | 0.05 | 0.10 | 0.10 | - | - | - |

**6.c – Mutant *rsa1***

Mutant *rsa1* is defective in ribosome biogenesis [19]. From experiments in glucose 2% it comes out that the population of *rsa1* shows an increased MDT, an increased G_1_ length for both daughter and parent cells as well as a reduction of the average protein content, see Fig P.

As previously stated, we faced the setting of the *iMeGroCy-2* to comply the mutant features by suitably exploiting the sensitivity analysis and the heat map as a library that sheds light on how parameter variations impact on population outputs. From the heat map in Fig 3D, it is apparent that crucial parameters involved are the ones related to growth (as expected from the kind of mutant). Among the ones highlighted from the heat map, we chose to modify (to reduce, actually, by about 36%) the relative protein synthesis rate. However, a satisfactory agreement with experimental population features did not arise from just one growth parameter variation, therefore we looked for parameters that could be of use. After a trial-and-error we opted to increase the Sizer parameter *k_on_* (the Cln3-Far1 binding coefficient is doubled), and to work on Timer parameters like *T_2_* (reduced by 40%) and *W_1_* (slightly increased of 0.32%). All these modifications (taken alone) help in improving one (or more) of the aforementioned mutant outputs: the fine-tuned combination of them all helps in reproducing *in silico* the qualitative behaviour provided by *rsa1* experiments. The length of Timer *T_B_* has been preliminary set to the mutant experimental value. On top of that, we furtherly increased the population parameter CV(P_cd_), i.e. the coefficient of variation of the protein/ribosome at division, to smooth the shape of the protein distribution and to avoid bimodality. This result suggests that mutant *rsa1* somehow provides a noisier partition at division. See Table M for a summary of the input parameter variations. Protein distributions and temporal features are reported in Fig P, compared to their experimental counterparts.


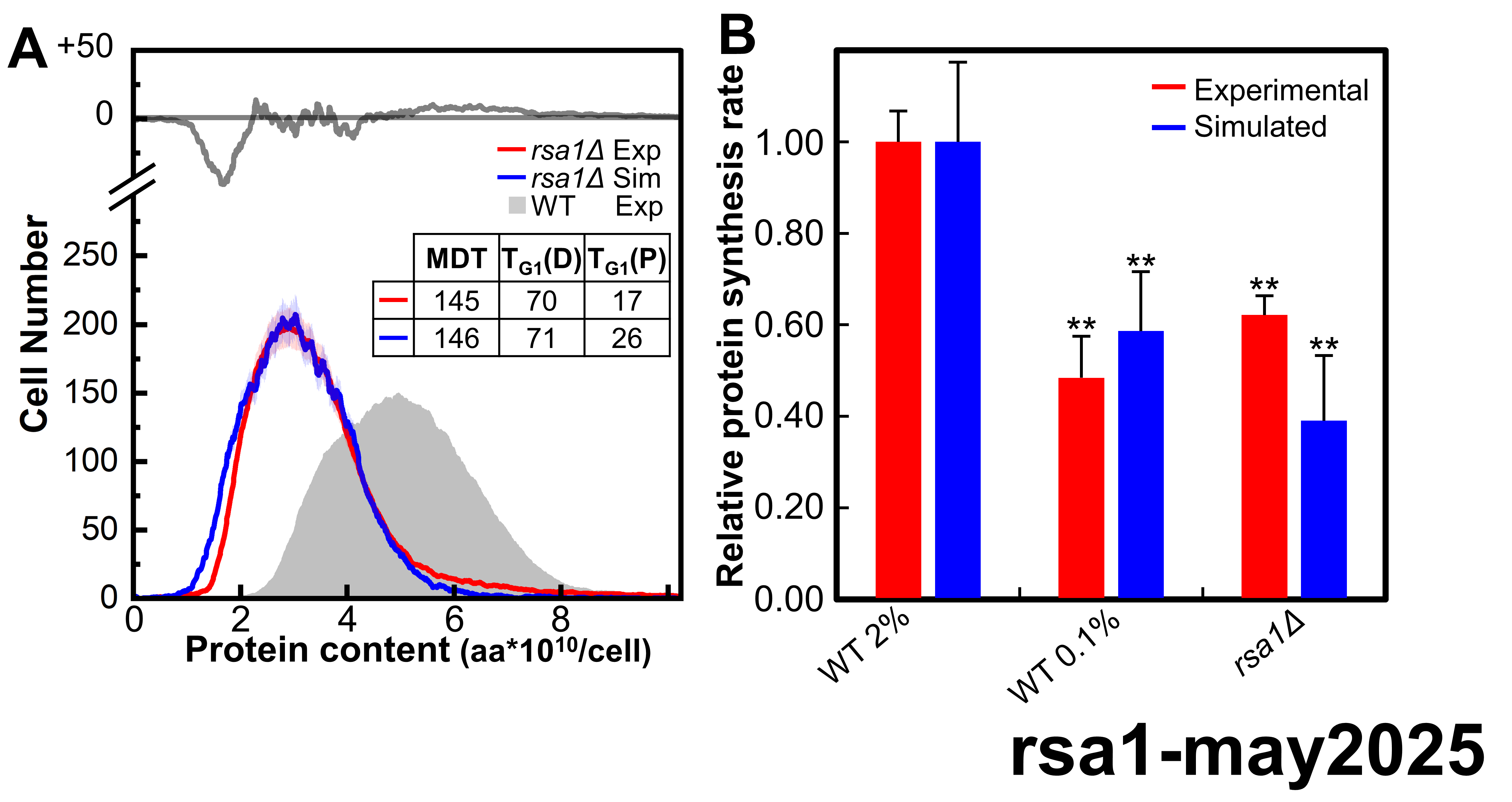


**Fig P. Mutant *rsa1*: simulated and experimental distributions and protein synthesis rate.** A) Comparison of experimental (red lines) and simulated (blue lines) protein distributions for the mutants rsa1 grown in glucose 2% media. Mean ± SD values are shown. The gray line in the upper insert in each panel is the difference between experimental and simulated values. The experimental distribution of wild-type cells cultivated in 2% glucose (filled gray) is also reported as a reference. The lower inserts show the temporal parameters for both experimental and simulated populations. B) Experimental (red bars) and simulated (blue bars) protein synthesis rates. Data are relative to the values measured for the wild-type strain cultivated in 2& glucose medium. Mean + SD values from at least three biological replicates are reported. Statistical significance: *p < 0.05, **p < 0.01, Student's t-test.

**6.d - *Mutants whi5Δ*** ***and whi5^4E^ (iMeGroCy-2 simulations)***

The mutants *whi5Δ* and *whi5^4E^* are two mutants of the *WHI5* gene [15,20,21]. In particular, the mutant *whi5Δ* lacks the *WHI5* gene while mutant *whi5^4E^* expresses a Whi5 protein whose functional phosphorylation sites have been mutated to phospho-mimetic glutamate residues. Both the mentioned mutations anticipate the entry into the S phase, originating small cells [1]. As *iMeGroCy-2* does not take the molecular details of the *WHI5* dynamics into account (which are described by *Hy-iMeGroCy-2* instead), we cannot perform fine simulations of these mutants. So, we try to render their qualitative behavior by means of the same *iMeGroCy-2* simulation, obtained by changing only one cycle parameter. Experiments in glucose 2% suggest that both *whi5Δ* and *whi5^4E^* mutants show, with respect to the WT, a reduction of the mean protein content and a light reduction of the G_1_ length, as well as a light increase of the budded phase and essentially the same MDT (there is a light increase only for *whi5^4E^*, see Table L, panel B). So, in order to reproduce the behavior of the pair of mutants we decided to: (i) not change parameters related to the growth module and to the timer *T_B_*, as the simulated MDT and *T_B_* length of the wild type population (99 min and, respectively, 85 min, see 2% glucose in Tables E and G,) already coarsely match the mutant experimental values, i.e., they are in the middle of the experimental pairs of MDT and *T_B_* (see Table L, panel B); (ii) increase parameter *W_1_* (of the 1.21% w.r.t. the WT value) in order to reduce the mean protein content of the mutant and, at the same time, obtain a little reduction of the T_G1_ length. The variation of the cycle parameter *W_1_* has been evaluated on the basis of the heat map reported in Fig 3D. See Table M for a summary of input parameter variations. Fig 8A-B reports experimental and simulated protein distributions and temporal features (*iMeGroCy-2* simulations are in dark blue).

**Table M. *iMeGroCy-2* input parameter variations for mutants *rsa1*, *whi5* and *whi5^4E^***

| **Parameters** | ***rsa1*** | ***whi5^4E^/whi5*** |
| --- | --- | --- |
|  | **glc 2%** | **glc 2%** |
| ***K****_2_* | 255.19 | - |
| ***K****_2_^1^* | 236.04 | - |
| ***K****_2_^2^* | 128.69 | - |
| ***K****_2_^3^* | 57.13 | - |
| ***K****_2_^4^* | 45.20 | - |
| ***K****_2_^5^* | 39.23 | - |
| ***K****_2_^s^, s > 5* | 34.46 | - |
| ***T****_B_* | 100 | - |
| ***T****_2_* | 4 | - |
| ***W****_1_* | 62.3 | 62.85 |
| ***k****_on_* | 3.26e-15 | - |
| ***CV(P_cd_)*** | 0.10 | - |

**6.e – Mutants *whi5^Δ^* and *whi5^4E^* (*Hy-iMeGroCy-2* simulations)**

By exploiting a mathematical model detailing about the molecular machinery where Whi5 is involved, we attempted to vary specific parameter values of the G_1_/S molecular module to obtain a population simulations predictive of the qualitative behavior of the mutant under investigation, similarly to what we did for mutants *hxk2 hxk1* and TM6*. Indeed, the fine-grain model *Hy-iMeGroCy-2* accounts for the specific *whi5Δ* mutant by simply setting total Whi5 = 0 in the molecular module whilst, for the simulation of the *whi5^4E^* mutant, expressing a protein whose 4 functional sites have been mutated to glutamate that acts as a constitutive phosphomimetic [15], we assume that *whi5^4E^* keeps unchanged the possibility to bind to (and therefore inhibit) SBF, but with a lower affinity, that means we reduce the value of *k_14_* to 1/10 of the nominal value in Table J. Moreover, because of the 'natural' phosphorylation state of Whi5, soon after the binding of SBF/Whi5, Whi5 is ready to be released without waiting for further phosphorylation(s). However, this happens at a lower rate with respect to the wild-type case, so that we modified the corresponding parameter, named *α_W_* in [15]. Fig 8A-B reports experimental and simulated protein distributions and temporal features (*Hy-iMeGroCy-2* simulations are in light blue).

***Table N Panel A. Growth parameters of the CEN.PK wild type strain***

***Table N Panel B. Growth parameters of various mutants***

Mutant strains are isogenic to either the CEN.PK or W303-1A wild type strains. Growth parameters were monitored during exponential growth at 30°C in SC/YNB medium supplemented with the indicated carbon sources. Data are means ± SDs of at least 3 independent experiments

**MDT** = Mass doubling time; **F_B_** = percentage of budded cells (determined by direct scoring of at least 400 cell); **T_B_** (length of budded S+G2+M phases) was calculated according to formula log_2_(1+FB)*MDT. **T_P_** (average doubling time of parent cells), **T_D_** (average doubling time of daughter cells) were evaluated by bud scar analysis.

**V** = mean cellular volumes (evaluated by Coulter counter cell analyzer). **P** = avg protein content; **P_s_** = avg protein content at START; **P_0_** = avg protein cells of daughetr cells at birth. Protein content were evaluated by cytofluorimetric analysis. **P_P_** (average content of parent cells) and **P_cd_** (avg protein content at division) were calculated from bud scar analysis according to the formulas described in Methods.

|  | **Ethanol yield** | |
| --- | --- | --- |
| **Medium [glucose]** | ***wild type*** | ***snf3 rgt2 gpa2 gpr1*** |
| **0.05%** | 0.73 (*) | 0.93 |
| **0.1%** | 0.91 * | 0.88 |
| **0.2%** | 1.04 * | 0.81 |
| **0.5%** | 1.07 * | 0.83 |
| **2%** | 1.32 | NA |
| **5%** | 1.50 | 0.78 |
| Ethanol yield. * indicates statistical significance in a parallelism test (95% confidence) relative to the 5% glucose medium (chosen as the reference growth condition). NA = data not available. | | |

**Table O. Ethanol yield**


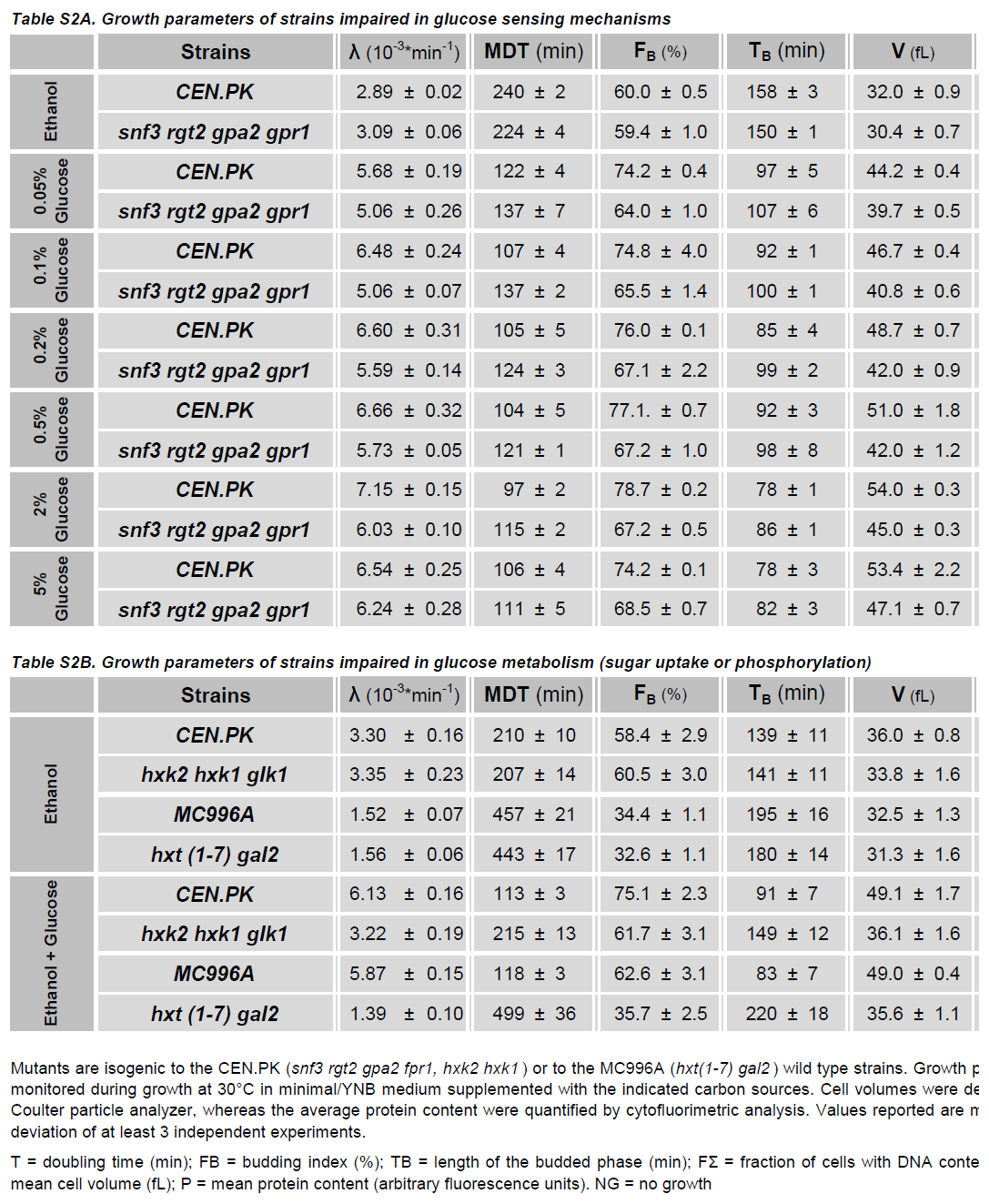
**Table P. Growth parameters of strains impaired in glucose sensing mechanisms**

Mutant strains are isogenic to either the CEN.PK wild type strains. Growth parameters were monitored during exponential growth at 30°C in SC/YNB medium supplemented with the indicated carbon sources. Data are means ± SDs of at least 3 independent experiments.

** = growth rate; **MDT** = Mass doubling time; **FB** = percentage of budded cells (determined by direct scoring of at least 400 cell); **TB** (length of budded S+G2+M phases) was calculated according to formula

log_2_(1+FB)*MDT.


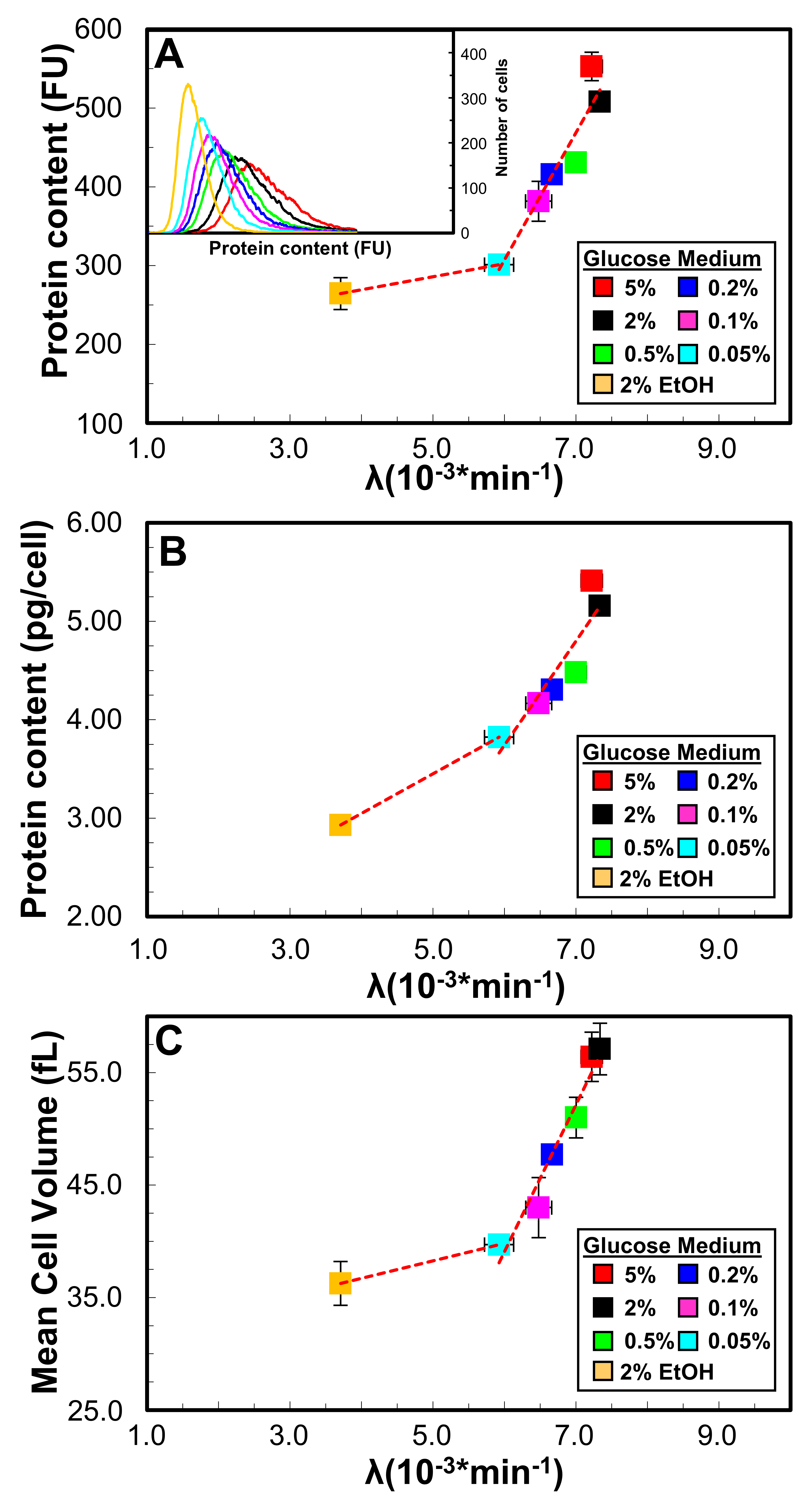


**Fig Q. Yeast cell size evaluated by different methods.**

**A)** The Average cellular protein content of cell population (measured by flow cytometry and expressed in Fluorescence Units (FU); **B)** the average protein content/cell (obtained by chemical dosage, expressed in pg/cell; **C)** the mean cell volume (measured by Coulter Counter, in fL). Values were plotted *vs.* the growth rate (*λ*, min^-1^) for wild type cells cultivated in media supplemented with different glucose concentrations or ethanol. Inserts in panel A show representative protein distribution profiles, determined by flow cytometry.

**
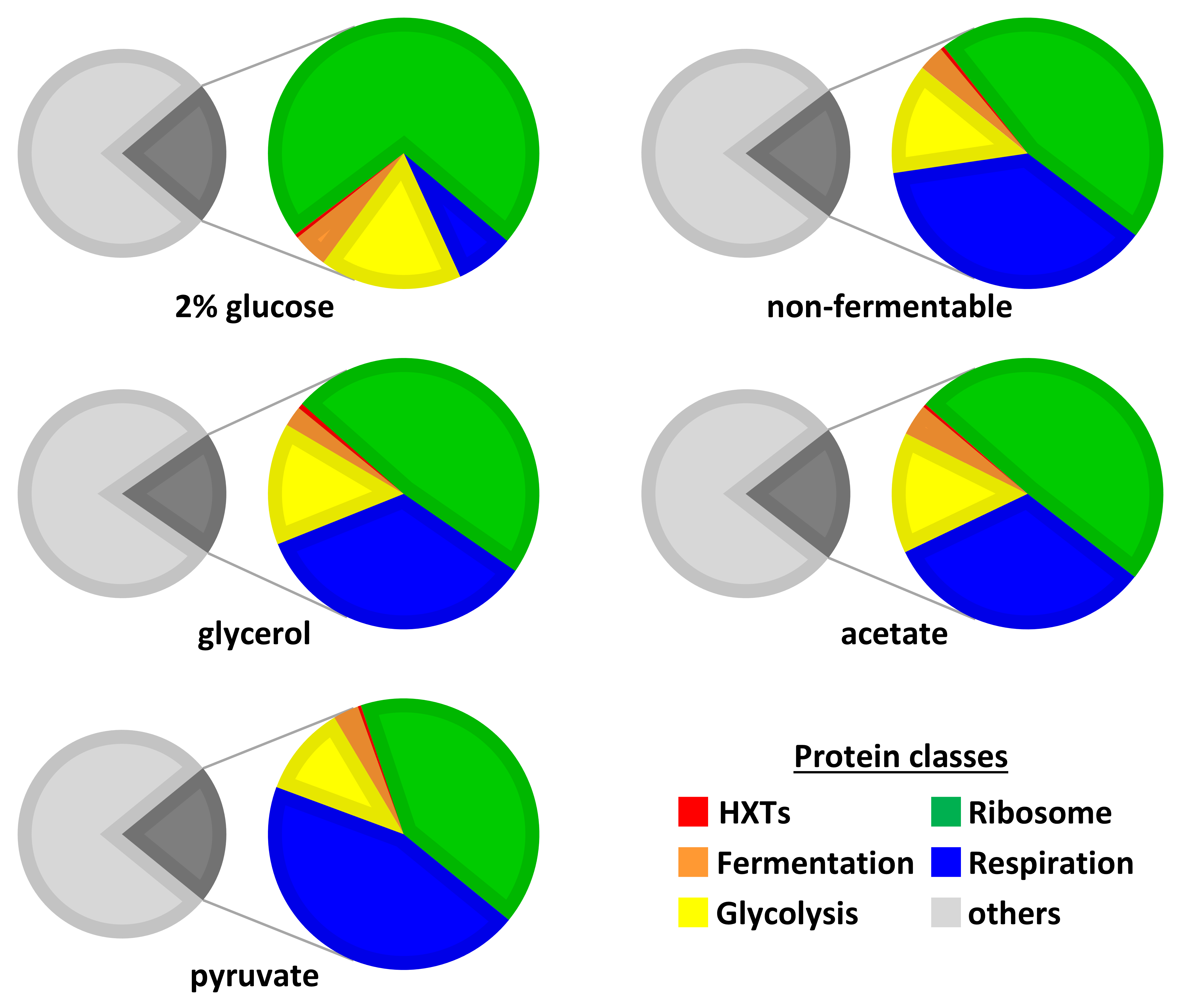
*Fig R. In vivo yeast proteome allocation during growth on various carbon sources.*** Values reported are % total proteins #/cell (data taken from [22]). “non-fermentable” is the average allocation during growth on three carbon sources glycerol, acetate and pyruvate (here also reported). Proteins were manually grouped into 6 classes according to their physiological functions: “glycolysis”, “fermentation”, “HXTs” (glucose carriers), “respiration”, “ribosome/translation” and “others”. In this dataset, these classes cover ~20% of the entire proteome in all the tested C-sources. For a complete list of all the proteins included in each class see S1 Data.

**

**

**Fig S*. Metabolism-driven modulation of cell size***

**A-D)** Growth rate (*λ*, min^-1^) vs. protein contents (*P, P_s_, P_0_*, Fluorescence Units) in media supplemented with different glucose concentrations or ethanol. *P* = average protein content of the entire cell population; *P_s_* = protein content at the onset of DNA replication; *P_0_* = protein content at the beginning of the cell cycle.

**E-H)** protein contents (*P, P_s_, P_0_*, Fluorescence Units) *vs.* ethanol yield

**I)** Glucose uptake capacity monitored by evaluating incorporation of the fluorescent 2-NBDG glucose analogue. Values are relative to the glucose uptake capacity of wild type cells cultivated in 2% glucose medium. The anomalous results of the *hxk2 hxk1* mutant are likely due to artifacts.

**J****)** Reduction of glucose kinase activity in the *hxk2 hxk1* mutant.


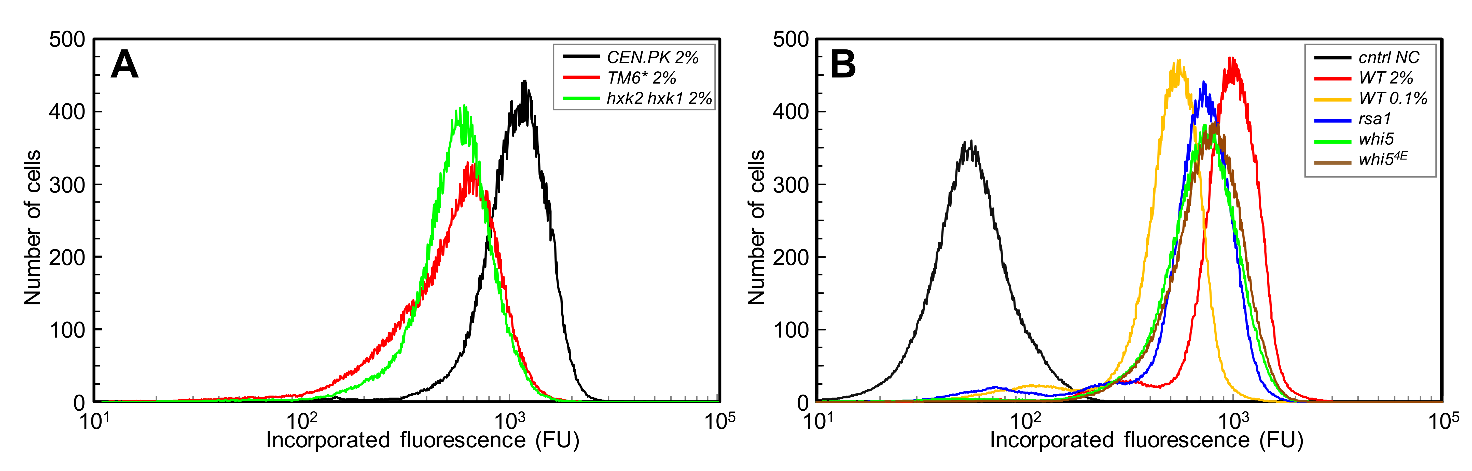


**Fig T. Protein Synthesis rate**

Fluorescence incorporated in neosynthesised proteins was measured by cytofluorimetric analysis. Representative distribution profiles from various strains and growth conditions are shown.

**References**

1. Palumbo P, Vanoni M, Papa F, Busti S, Wortel M, Teusink B, et al. An integrated model quantitatively describing metabolism, growth and cell cycle in budding yeast. In Communications in Computer and Information Science  830; 2018.

2. Molenaar D, van Berlo R, de Ridder D, Teusink B. Shifts in growth strategies reflect tradeoffs in cellular economics. Mol Syst Biol. 2009;5:323.

3. Alberghina L, Mariani L, Martegani E. Cell cycle modelling. Biosystems. 1986;19(1):23–44.

4. Palumbo P, Papa F, Vanoni M, Alberghina L. A Coarse-Grain Model of Growth and Cell Cycle in Saccharomyces Cerevisiae: a Mathematical Analysis. Acta Polytechnica Hungarica. 2019;16(10):205–24.

5. Porro D, Vai M, Vanoni M, Alberghina L, Hatzis C. Analysis and modeling of growing budding yeast populations at the single cell level. Cytometry Part A. 2009;75A(2):114–20.

6. Wortel MT, Bosdriesz E, Teusink B, Bruggeman FJ. Evolutionary pressures on microbial metabolic strategies in the chemostat. Vol. 6, Sci Rep. 2016. p. 29503.

7. Woolford JL, Baserga SJ. Ribosome Biogenesis in the Yeast Saccharomyces cerevisiae. Genetics. 2013;195(3):643–81.

8. Sánchez BJ, Zhang C, Nilsson A, Lahtvee P, Kerkhoven EJ, Nielsen J. Improving the phenotype predictions of a yeast genome‐scale metabolic model by incorporating enzymatic constraints. Molecular Systems Biology. 2017;13(8):935.

9. Alberghina L, Rossi RL, Querin L, Wanke V, Vanoni M. A cell sizer network involving Cln3 and Far1 controls entrance into S phase in the mitotic cycle of budding yeast. J Cell Biol. 2004;167(3):433–43.

10. Schmidt-Glenewinkel H, Barkai N. Loss of growth homeostasis by genetic decoupling of cell division from biomass growth: implication for size control mechanisms. Mol Syst Biol. 2014;10(12):769.

11. Murray LE, Veinot-Drebot LM, Hanic-Joyce PJ, Singers RA, Johnston GC. Effect of ploidy on the critical size for cell proliferation of the yeast Saccharomyces cerevisiae. Curr Genet. 1987;11(8):591–4.

12. Sherman F. Getting started with yeast. In: Guthrie C, Fink GR, editors. Methods in Enzymology [Internet]. Academic Press; 2002 [cited 2025 May 19]. p. 3–41. (Guide to Yeast Genetics and Molecular and Cell Biology - Part B; vol. 350). Available from: https://www.sciencedirect.com/science/article/pii/S007668790250954X

13. Jorgensen P, Nishikawa JL, Breitkreutz BJ, Tyers M. Systematic identification of pathways that couple cell growth and division in yeast. Science. 2002;297(5580):395–400.

14. Zhang J, Schneider C, Ottmers L, Rodriguez R, Day A, Markwardt J, et al. Genomic Scale Mutant Hunt Identifies Cell Size Homeostasis Genes in *S*. *cerevisiae*. Current Biology. 2002;12(23):1992–2001.

15. Palumbo P, Vanoni M, Cusimano V, Busti S, Marano F, Manes C, et al. Whi5 phosphorylation embedded in the G1/S network dynamically controls critical cell size and cell fate. Nat Commun. 2016;7:11372.

16. Diderich JA, Raamsdonk LM, Kruckeberg AL, Berden JA, Dam KV. Physiological Properties of Saccharomyces cerevisiae from Which Hexokinase II Has Been Deleted. Applied and Environmental Microbiology. 2001;67(4):1587–93.

17. Raamsdonk LM, Diderich JA, Kuiper A, van Gaalen M, Kruckeberg AL, Berden JA, et al. Co-consumption of sugars or ethanol and glucose in a Saccharomyces cerevisiae strain deleted in the HXK2 gene. Yeast. 2001;18(11):1023–33.

18. Otterstedt K, Larsson C, Bill RM, Ståhlberg A, Boles E, Hohmann S, et al. Switching the mode of metabolism in the yeast Saccharomyces cerevisiae. EMBO Rep. 2004;5(5):532–7.

19. Kressler D, Doère M, Rojo M, Linder P. Synthetic lethality with conditional dbp6 alleles identifies rsa1p, a nucleoplasmic protein involved in the assembly of 60S ribosomal subunits. Mol Cell Biol. 1999;19(12):8633–45.

20. de Bruin RAM, McDonald WH, Kalashnikova TI, Yates J, Wittenberg C. Cln3 Activates G1-Specific Transcription via Phosphorylation of the SBF Bound Repressor Whi5. Cell. 2004;117(7):887–98.

21. Costanzo M, Nishikawa JL, Tang X, Millman JS, Schub O, Breitkreuz K, et al. CDK Activity Antagonizes Whi5, an Inhibitor of G1/S Transcription in Yeast. Cell. 2004;117(7):899–913.

22. Paulo JA, O’Connell JD, Everley RA, O’Brien J, Gygi MA, Gygi SP. Quantitative mass spectrometry-based multiplexing compares the abundance of 5000 S. cerevisiae proteins across 10 carbon sources. J Proteomics. 2016;148:85–93.
