# Supplementary material for: A modular model integrating metabolism, growth, and cell cycle predicts that fermentation is required to modulate cell size in yeast populations
Source: PLoS Comput Biol. 2025 Jul 21;21(7):e1013296. doi: 10.1371/journal.pcbi.1013296 (PMC12377586; doi:10.1371/journal.pcbi.1013296)
Supplement: Attachment — Submitted filename: Authors_Response_Final.docx [file pcbi.1013296.s004.docx]

**REVIEWER #1**

*In this work, Vanoni et al. present a coarse-grain mathematical model linking metabolism, cell growth and cell cycle to predict the regulatory role of fermentation in modulating cell size within yeast populations. The model builds on the author’s previous model (Palumbo et al 2018) where MeGro and GroCy modules are presented. Comments:*

R1.1. *Considering that the model builds on the author's previous model, it would be good to be clear about the actual novelty of this work. This is also reflected in the mathematical description of the models, which comes directly from previous publications.*

**Answer:**

We thank the Reviewer for the observation. To highlight the originality and the novelty of the presented work, we did the following:

- As explicitly requested by another Reviewer, a unified detailed description of the model is presented in the Supplementary Material, which also compares the current and old versions of the different models and their integration. The novel Supplementary Figure S1 and Tables 1-2 compare the structure of the old *MeGro* (Ref [39] of the new manuscript and Ref [Ref1-1] of this document) and the novel *Megro-2* modules and the features that differentiate them.

Supplementary Figure S1, and Tables 1-2 (see next paragraphs) are reported below for convenience.

**Supplementary Figure S1—** Comparison between the extended version of the Metabolism & Growth module newly presented in this manuscript (*MeGro-2*) and its previously published version (*MeGro*, on the left hand-side). *MeGro-2* can now have ethanol as a nutrient (bold ovals for internal and external ethanol), therefore allowing ethanol respiration as well as gluconeogenesis from ethanol (bold lines in *MeGro-2*).

**Table 1 – *MeGro-2-vs-MeGro* differences**

| **Feature** | ***MeGro* [39]** | ***MeGro-2* (this paper)** |
| --- | --- | --- |
| Usable Carbon source | Glucose only | Glucose and ethanol, tunable to other carbon sources |
| Gluconeogenesis | No | Yes |
| Respiration | Only from pyruvate | From both pyruvate and ethanol |

**Table 2 – *iMeGroCy-2-vs-iMeGroCy* applications**

| **Application** | ***iMeGroCy* [39]** | ***iMeGroCy*-2 (this paper)** |
| --- | --- | --- |
| Single cell | Yes | Yes |
| Population-level | No, only small cell clusters can be analyzed | Yes, allowing direct comparison of simulation results with readily determinable features (i.e., protein distributions obtained by flow cytometry) of populations in balanced or perturbed exponential growth |
| Mutant analysis | Yes, in principle, but no direct comparison with readily determinable population features is possible | Yes, allowing direct comparison of simulation results with readily determinable features (i.e., protein distributions obtained by flow cytometry) of populations in balanced or perturbed exponential growth |
| Open to plug-ins | Yes, in principle, but no direct comparison with readily determinable population features is possible | Yes, allowing direct comparison of simulation results with readily determinable features (i.e., protein distributions obtained by flow cytometry) of populations in balanced or perturbed exponential growth |

- To allow an understanding of the design and potentiality of the integrated Model without resorting to Supplementary Materials, a brief outline of the current model is presented in the first section of the Results. Table 1 and Table 2 (the latter having been moved from one of the supplementary files of version 1 of the manuscript) indicate why all computational simulations (and their comparison with experimental data) presented in Figures 4-6 of the revised manuscript *would not be possible* with the old version of the integrated model *and require* the novel model.

In fact:

- The original *MeGro* does not support growth in Ethanol. *MeGro-2* is now able to sustain ATP production and the synthesis of building blocks not only from glucose-fed (as in the original model) but also through ethanol-fed metabolic pathways: (1) the ethanol respiration and (2) the gluconeogenic pathways producing pyruvate from ethanol, which in turn can feed both fermentation and respiration. So, the internal ethanol can be both a by-product of the fermentation and a metabolic source for *MeGro-2*, and it can be exchanged with the external environment (in both directions). This modification is *required* to investigate how cells modulate their size according to different environments and/or genetic perturbations, which is the primary biological insight of the manuscript (see following points).
- The original integrated model does not include a population layer and does not allow direct comparison with experimentally growing cell populations. Therefore, the inclusion in the updated model of a population layer is mandatory to reproduce *in silico* experimental features of cell populations in balanced or perturbed exponential growth. Because the size distribution of asynchronously growing yeast cells is related to protein (biomass) accumulation during the cell cycle, the law of cell division of the population, and its structure, the size (protein) distribution acts as a fingerprint for each condition of balanced exponential growth (Figure 1B and reference [14] of the new manuscript and reference [Rev1-2] of this document). Therefore, we use the distribution of the protein content - measured on cell populations stained by fluorescein isothiocyanate and analyzed by cytofluorimetric analysis as a segregated parameter – as the major experimental parameter to be compared with the simulation outputs.
- Applying the (novel) population model and Occam Razor’s limited tuning set of input parameter(s) allows us to:
  - describe cell populations growing in a medium supplemented with different quality and quantity of carbon source, from 2% ethanol to 5% glucose;
  - identify the emergent property that cells with constitutive respiration do not modulate cell size according to the growth conditions and validate the prediction with mutants unable to ferment;
  - pinpoint the cellular functions altered in mutants;
- As a proof-of-principle of the ability of our integrated model to act as a scaffold for molecularly detailed models of a selected function (that in the coarse-grain model is described by one or a few equations), we inserted a previously published molecular module for the G_1_/S transition that partly replaces the *GroCy* machinery. The resulting hybrid *Hy-iMeGroCy-2* model retains the general population dynamics of cell growth and division, quantitatively describes the temporal features and the protein distributions of a wild-type population, and allows molecular interpretation of the behavior of cell cycle mutants that could only be described at the cellular level by the integrated coarse-grain model.

R1.2. *Many of the simulation results presented indicated that some sort of noise is incorporated into the model. On page 25 under Statistical analysis the Authors refer to some sort of binning procedure. But I wonder if this can actually correctly represent the noise of the system. Can you please explain how the noise is incorporated?*

**Answer:**

In the original manuscript, *binning* refers to the procedure required to directly compare experimental and simulated protein distributions. To avoid misunderstanding, we cleared the term from the paper. In the revised manuscript, the paragraph “Statistical analysis”, refers to the statistical analyses carried out on the main outputs of the population, such as the protein content, timer lengths, and Mass Duplication Time, while a separate section (“Comparisons of experimental and simulated distributions” reported at the end of Methods Section of the main text) details the procedure to compare experimental and simulated distributions.

Noise is incorporated in the population simulations, and it involves the timer lengths and the accuracy of asymmetrical cell division. More in detail:

- whenever a cell is born, the lengths of the timers T_1b_, T_2_ or T_B_ are sampled from lognormal distributions, with average values coming from Eq.(S30) for T_1b_ and from Supplementary Table S5 for T_2_ and T_B_, and a coefficient of variation equal to 5%.
- at cellular division, the newborn parent cell starts with an initial protein content drawn from a lognormal distribution with an average value given by the critical size Ps achieved by the parent cell at the onset of the budded phase. The coefficient of variation is taken to be smaller and smaller for newborn parent cells of increasing genealogical age, that is 5% for a parent born from a daughter or from a parent *P_k_*, *k* = 1,…,5, while CV = 4%, 3% and 2% for a parent born from *P_6_*, *P_7_* and *P_k_*, *k* > 7, respectively; the newborn daughter cell starts with an initial protein content given by the difference between the protein content at division and the newborn parent cell initial size; the newborn daughter/parent ribosome content proportion mimics the newborn daughter/parent protein content. In the revised version of the manuscript, the Reviewer can find this part in Supplementary Material, Section 5.c, where we also added a sentence to better explain where noise is incorporated in the population model.

R1.3. *The procedure for ‘adjusting the parameters’ sounds like an ad hoc method with no proper quantification of the trustworthiness of the obtained parameters. Instead of the classical trial and error procedure, as the authors name it, it would be better to use more established methods (for example, some Bayesian approaches to automatically tune the enzyme kcat values from proteomics-constrained GEMs) The way that procedure is now done may also be the reason that the parameters are led to the state in which they will show ‘excellent agreement between experimental and computational protein distribution (as indicated on p12 and Fig 6), but there are no quantitative values shown to evaluate the fitness.*

**Answer:**

Of the reactions included in *MeGro-2*, 5 over 7 are not individual biochemical reactions but rather fluxes of lumped reactions that do not have a direct counterpart in GEM models, as summarized in the Table reported below (red fluxes). Moreover, unlike reactions in GEM models, *MeGro-2* reactions are modeled by Michaelis-Menten functions (see Eqs.(S6) in the Supplementary Material, Section 3), making the direct transfer of *k_cat_* values from proteomics-constrained GEMs not straightforward.

| **Fluxes** | |
| --- | --- |
| $v_{hxt}$ | Glucose transport |
| $v_{gly}$ | Glycolysis |
| $v_{resp}$ | Respiration from pyruvate |
| $v_{ferm}$ | Fermentation |
| $v_{rib}$ | Biomass production |
| $v_{{EtOH}_{tr}}$ | Ethanol transport |
| $v_{gly2}$ | Gluconeogenesis |
| $v_{resp2}$ | Respiration from ethanol |

Since *MeGro-2* is an extension of the corresponding *MeGro* program published in [Ref1-1], most of the module parameters are inherited from that reference (where the parameters have been set following the same criteria developed in [Ref1-3]) since they refer to pathways shared by both *MeGro* and *MeGro-2* (minor modifications have been done for some of them, mostly related to the use of different units). We do not change any of the internal parameters of *MeGro-2* when simulating different glucose concentrations and make only minor adjustments when modeling ethanol growth (see Supplementary for further details). For the glucose simulations, the outputs of the program only depend on the two input parameters, i.e., the glucose concentration and the corresponding respiratory ratio that we calculate from the following formula (Eq.(S21) in Supplementary Material)

$$F\approx20\frac{Y_{EtOH/glc}}{7+ 15 Y_{EtOH/glc}}$$

using values determined from biochemical assays of glucose consumption and ethanol production in any growth condition (the *F* parameter has no meaning for ethanol simulation).

As the graph below shows, *MeGro-2* accurately predicts the growth rate *λ* in all the simulated conditions. This indicates that the parameter set used in *MeGro-2* captures growth rate modulations of yeast cells in various environmental conditions, which gives solid grounds for the robustness of this parameter set.

iMeGroCy2 λ (relative)

| Experimental λ (relative) | lambda |
| --- | --- |
| 0.493151 | 0.561644 |
| 0.808219 | 0.767123 |
| 0.876712 | 0.835616 |
| 0.917808 | 0.890411 |
| 0.958904 | 0.958904 |
| 1 | 1 |
| 0.986301 | 1.0137 |

**Supplementary Figure S2. Correlation between experimental and simulated values for the growth rate λ parameter**. Values reported are relative to the 2 % growth condition, taken as reference. The green bisector line indicates the ideal, perfect correlation between the two data series.

This conclusion is reinforced by a sensitivity analysis of the *MeGro-2* model parameters in which we analyzed the effect of the variation of the catalytic coefficients *k_cat_* of the kinetic equations of the fluxes of *MeGro-2*, on the major output parameter, the exponential growth rate *λ*. The sensitivity analysis has been performed by fixing the *MeGro-2* inputs *F* and $c_{glc,ex}$ to the experimental values related to 2% glucose reported in the heading of Supplementary Table S.5. The results of this analysis are depicted in Supplementary Fig. S6 and reported below for convenience. Each panel shows the variations of the output parameter *λ* with respect to its value in 2% glucose when the nominal *k_cat_* (reported in Supplementary Table S.3) is varied of 0.01x, 0.1x, 10x, 100x. The analysis shows that only the catalytic coefficient *k_cat,gly_* has a sizable effect on the output parameter *λ*. Note that this analysis indicates that this parameter is already set at the highest value compatible with a biologically meaningful growth rate.

**Supplementary Figure S6 – Sensitivity analysis of the *MeGro-2* output λ with respect to *k_cat_* parameters** Relative ratios of *λ* (normalized w.r.t. the values in 2% glucose) as a function the coefficient of variation of *k_cat,x_*, *x ∈ Prot2.*

[Ref1-1] Palumbo P, Vanoni M, Papa F, Busti S, Wortel M, Teusink B, et al. An integrated model quantitatively describing metabolism, growth and cell cycle in budding yeast. In Communications in Computer and Information Science 830; 2018.

[Ref1-2] Porro D, Vai M, Vanoni M, Alberghina L, Hatzis C. Analysis and modeling of growing budding yeast populations at the single cell level. Cytometry Part A. 2009;75A(2):114–20.

[Ref1-3] Wortel MT, Bosdriesz E, Teusink B, Bruggeman FJ. Evolutionary pressures on microbial metabolic strategies in the chemostat. Vol. 6, Sci Rep. 2016. p. 29503.

**REVIEWER #2**

*R2-1	In this manuscript, the authors update an existing modular model of yeast growth with more detailed modules to make some predictions on how changes in environmental and genetic conditions affect the distribution of cell sizes in yeast. Given the way that the results are currently presented in the manuscript, however, I was very confused as to how the model was built, how their parameters were determined, and what outputs from the model are actual “predictions” as opposed to model outputs that were simply fit to experimental data.*

**Answer:** Overall, the model's explanation and usage have been streamlined. The first two chapters of the **Results** summarize the structure of the integrated model (now called *iMeGroCy-2*), its components (*Megro-2* and *Grocy-2*), and their interconnection. They also discuss the significance of the input parameters and their robustness with respect to the output parameters. Finally, they briefly summarize the construction of the sensitivity analysis.

This part of the main text has been almost wholly rewritten compared to the original manuscript. We have pasted below the text and relevant figures for the reviewer's convenience.

**From Results**

**General structure and novel functionalities of the *iMeGroCy-2* model**

*iMeGroCy-2* is a substantial evolution of the previously published *iMegroCy* model [39] and consists of the interconnection of two modules: (i) a Metabolism & Growth (*MeGro-2*) and a Growth & Cycle (*GroCy-2*) module. *MeGro-2* includes lumped glycolytic and gluconeogenic pathways, a fermentation pathway, and a respiratory pathway, fed by either pyruvate or ethanol, a glucose transporter unit, bidirectional ethanol transport, and ribosomes devoted to biomass production. Ethanol derived from the outside can be respired or used in a simplified gluconeogenic pathway.

*MeGro-2* converts the available carbon source into energy (ATP) and macromolecules and uses a growth optimization algorithm to provide growth-related parameters to the *GroCy-2* module. Therefore, *MeGro-2* acts as a parameter generator, receiving two inputs for each growth condition: the external carbon source concentration ($c_{{glc}_{ex}}$ or $c_{{EtOH}_{ex}}$) and the fermentation ratio *F*, calculated from the experimental ethanol/glucose yield Y_EtOH_ for glucose-grown cells or set to 0 for ethanol-grown cells. Using these inputs, *MeGro-2* maximizes the exponential growth rate *λ*, accurately predicting it in different conditions that use as input different glucose concentrations (and their corresponding ethanol/glucose yield) or ethanol (Supplementary Fig. S2).

In *GroCy-2,* growth and cell cycle parameters not directly provided by *MeGro-2* are directly input by the user (see the Supplementary Material for the details on how *iMeGroCy-2* works and how its parameter values are set, Section 5). Cell growth is modeled by an Ordinary Differential Equation (ODE) system describing ribosome and protein dynamics (synthesis and degradation). A cyclin-mediated switch triggers the cell cycle, modeled as a series of consecutive timers. Timers *T_1a_*, *T_1b_*, and *T_2_* encompass the unbudded, G_1_ phase. *T_B_* corresponds to the budded phase, which includes the S, G_2_+M, and G_1*_ phases, of which the latter corresponds to the period that separates nuclear and cell division. The end of the last timer triggers cell division. The model incorporates a pedigree structure that follows the destiny of 5-10 cell clusters, each originating from a single founder cell (Fig.2), whose parameters are recorded. Cell-to-cell noise is introduced at cell division in newborn cells, giving rise to a simulated yeast population whose output temporal and cell growth parameters (notably protein distributions) can be directly compared with the equivalent parameters of experimental populations. Supplementary Fig. S5E presents a prototypical protein distribution generated by *iMeGroCy-2* for the case of 2% glucose in comparison with its corresponding experimental distribution. The Supplementary Materials file presents a detailed description of the integrated *iMeGroCy-2* model.

Tables 1 and 2 indicate why all computational simulations (and their comparison with experimental data) presented in Figures 4-6 *are only possible* with the new version of the integrated model. In fact: (i) only the revised *MeGro-2* supports growth in ethanol (see Supplementary Fig. S1 and accompanying text for a detailed comparison of *MeGro* and *MeGro-2)*; the presence of the population layer in *GroCy-2* is required (ii) to investigate how cell populations modulate their size according to different environments and/or genetic perturbations (which is the central biological insight of the manuscript) and (iii) to allow the model to act as a host for molecularly detailed modules, whose behavior can be studied within a context in which the general population dynamics are retained and act as constraints on the plugged-in module.

***iMegroCy-2* robustly describes the structure of exponentially growing yeast populations.**

To evaluate the robustness and biological significance of the identified parameter set, we first assessed the effect of each input parameter on the output growth and temporal parameters. Using the glucose 2% parameter set as a reference set, we conducted a sensitivity analysis by changing a single input parameter at a time while keeping the remaining input parameters constant. The monitored growth outputs are the average value of the protein distribution (<P>), its standard deviation (SD(P)), and coefficient of variation (CV(P)). The monitored temporal outputs are the mass duplication time of the population (MDT) and the length of the G_1_ phase of parent and daughter cells (T_G1D_ and T_G1P_, respectively). Each input parameter has been moved "forward" and "backward" compared to its nominal value. For each parameter setting (resulting from each parameter variation), the exponential growth of the yeast population was simulated, linking the *GroCy* input parameters to the quantitative features of the cell population. Supplementary Material, Subsection 5.d. provides details on the procedure. Figures 3A-C report an example of the sensitivity analysis for input parameter *T_2_*. Supplementary Figures S7-10 show the complete results of the sensitivity analysis summarized as a heat map in Fig. 3D. The heat map shows that the perturbation of many input parameters has limited or no effect on the tested outputs, indicating that the model is robust against parameter alterations. At the same time, the map provides suggestions on which parameters could be worth changing to obtain a simulated cell population more closely resembling an experimental yeast population perturbed by chemical or genetic means, thereby hinting at the cellular functions altered by the perturbation (see later chapters for examples).

**Figure 1.** **Cell cycle and cell size in yeast**

(A-B)	Schematic view of the cell cycle with cycle times for parents (P) and daughters (D) with different genealogical ages; T_G1_ and T_B_ are the lengths of the unbudded (G_1_) and budded (S+G_2_+M) phases, respectively.

(B) Cell size (protein content distribution obtained by flow cytometry).

**Figure 2.** **Structure and utilization of the integrated Metabolism Growth and Cycle (*iMeGroCy-2*) model**

Upper part: *iMeGroCy-2* (purple box) modules interconnections. *MeGro-2* (red box) and *GroCy-2* (orange box), with the *Growth* (blue box) and *Cycle* (green box) modules included in *GroCy-2*. Black and grey arrows inside *MeGro-2* box denote metabolic conversions and protein synthesis, respectively, whilst rectangles and ovals denote protein classes and metabolites, respectively.

Lower part: *GroCy-2* also incorporates a population pedigree framework following the destiny of germinal lines originated from distinct single progenitor cells. Parent (P, grey) and daughter (D, white) cells of different genealogical ages (as defined by the subscript numbers) are depicted in the pedigree map (lower part of the cartoon).

**Figure 3.** **Sensitivity analysis**

(A-C)	Sensitivity analysis with respect to T_2_ (glucose 2%). (A): simulated protein distributions drawn for different values of T_2_ and compared to the experimental one related to 2% glucose. (B): protein features (average and initial cell protein content, critical cell size and size at division) extracted from the simulated populations of panel A, endowed with their standard deviations. (C): G_1_ phase length for the whole population and for the subpopulations of daughters and parents, related to the simulated populations of panel A, endowed with their standard deviations.

(D)	Heat map describing the alterations in major output growth and temporal parameters obtained by changing the input parameters of the *GroCy-2* module. The considered temporal parameters are the mass duplication time of the population (MDT) and the G_1_ phase lengths of parent (T_G1Par_) and daughter cells (T_G1Dau_). The growth parameters are the average value (P), standard deviation (SD(P)) and coefficient of variation (CV(P)) of the cellular protein content.

(E)	Flow chart of the identification procedure for mutational and environmental perturbations.

The procedures followed for analysis of the different growth conditions or the mutants are now better detailed in the Supplementary Materials and summarized in the Main text. By way of example, the following text is pasted from the third chapter of the revised manuscript:

***Metabolism drives the coordination between cell growth and division***

First, we proved that our model could simulate different nutritional conditions using roughly the same parameters. As a first step, we run *MeGro-2,* varying its inputs (i.e., different glucose or ethanol concentrations and the fermentative-to-respiratory ratio), keeping all internal model parameters fixed at all nutritional conditions. Indeed, a change in the values of *K*_2_ and *ρ* alone (i.e., the *MeGro-2*-produced outputs) satisfactorily reproduces the output features of yeast populations grown in 5% and 0.5 % glucose (Fig. 4 C-E). For lower glucose concentrations, this approach captures the reduction in overall dimension and the decrease in growth rate. Still, it leaves space for further improvements, as shown for protein distributions in Supplementary Fig. S4. As explained in Supplementary Materials, a minimum of 2 and a maximum of 7 parameters (out of a total of 31) required minor adjustments from the values of the 2% condition. These changes were suggested mainly by experimental data (Supplementary Table S12A) and literature (see Subsection 5b in Supplementary Material for the details) and allowed the final distributions and corresponding outputs to be obtained, as reported in Fig.4 and Supplementary Table S7.

The new **Supplementary Material**, whose structure is reported below for the reviewer's convenience, details the integrated mathematical model, **its significance** and setting of input parameters, and the simulation of mutant populations.

1) ***iMeGroCy-2* in a nutshell**
2) ***iMeGroCy-2 vs iMeGroCy***
3) ***The MeGro2 module***
   a. *The optimization problem associated to pure glucose environment (*$c_{{EtOH}_{ex}}=0$*)*
   b. *The optimization problem associated to pure ethanol environment (*$c_{{glc}_{ex}}=0$*)*
4) ***The GroCy-2 module***
   a. *Asymmetrical division and genealogical age heterogeneity*
5) ***Working of iMeGroCy-2 and parameter setting***
   a. *MeGro-2 parameter setting*
   b. *GroCy-2 parameter setting*
   c. *Simulations of cell populations through iMeGroCy-2*
   d. *Sensitivity analysis*
   e. *Simulations of cell populations through Hy-iMeGroCy-2*
6) ***Simulation of mutants populations***
   a. *Mutant hxk1 hxk2*
   b. *Mutant TM6**
   c. *Mutant rsa1*
   d. *Mutants whi5^Δ^ and whi5^4E^ (iMeGroCy-2 simulations)*
   e. *Mutants whi5^Δ^ and whi5^4E^ (Hy-iMeGroCy-2 simulations)*
7) ***Additional experimental Tables and Figures***

*R2.2	While I can see the computational model presented in the manuscript as being potentially useful to the scientific community, I am not entirely persuaded by the points made by the authors in the current version of the manuscript. I would like to see the authors address these suggestions before I can make a decision on this manuscript: The paper adds a few updates to an existing model of yeast metabolism, growth, and cell cycle that enables new functionalities. As someone without any prior knowledge of this original model, it was extremely difficult to understand how the original model is structured and the significance of these updates. The authors briefly summarize the changes made to the MeGro module and the structure of the GroCy module in the first few paragraphs of the Results section, but I would suggest starting off with a few paragraphs on just describing the mathematical structures of these modules and how the two modules are connected, some of which the authors get to in later paragraphs of the same section. I understand that the detailed explanation of the original model must be delegated to previous publications / supplementary materials to a certain extent, but readers should not be expected to have any knowledge about the previous version to follow this work just from the main text. I also think somewhere like Figure 2A would be a great place to explicitly highlight which parts of the model were newly added with this update versus which parts of the model existed in the original version.*

**Answer:** As outlined above in response to question R2.1, the revised manuscript has been heavily restructured to answer the concerns of this and other reviewers. As requested here, the paper now starts with a section that recapitulates the structure and wiring of the *iMeGroCy-2* Integrated Model and its components. To highlight *iMegroCy-2* improvements and unique abilities over the original *iMegroCy*, we added Supplementary Figure S1 (reported below for the reviewer’s convenience) that compares the wiring of the old *MeGro* module to its extended version *MeGro-2*. This figure highlights the significant differences allowing *MeGro-2* to sustain the production of energy synthesis of the building blocks in the absence of extracellular glucose through two new metabolic pathways fed by ethanol (i.e., the ethanol respiration and the gluconeogenic pathway producing pyruvate from ethanol).

**Supplementary Figure S1 –** The panel on the right-hand side shows the structural differences between the extended version of the metabolism & growth module (*MeGro-2*) compared to its preliminary version (*MeGro*, on the left hand-side). *MeGro-2* is now able to have also ethanol as nutrient (bold ovals for internal and external ethanol), according to which ethanol respiration is now allowed as well as gluconeogenesis from ethanol (bold lines in *MeGro-2*).

**Table 1** and **Table 2** (the latter having been moved from one of the supplementary files of version 1 of the manuscript) indicate why all computational simulations (and their comparison with experimental data) presented in Figures 4-6 of the revised manuscript *would not be possible* with the old version of the integrated model *and require* the novel model.

**Tables 1** and **2** are reported below for the reviewer’s convenience

**Table 1 – *MeGro-2*-vs-*MeGro* differences**

| **Feature** | ***MeGro* [39]** | ***MeGro-2* (this paper)** |
| --- | --- | --- |
| Usable Carbon source | Glucose only | Glucose and ethanol, tunable to other carbon sources |
| Gluconeogenesis | No | Yes |
| Respiration | Only from pyruvate | From both pyruvate and ethanol |

**Table 2 – *iMeGro-2*-vs-*iMeGro* applications**

| **Application** | ***iMeGroCy* [39]** | ***iMeGroCy*-2 (this paper)** |
| --- | --- | --- |
| Single cell | Yes | Yes |
| Population-level | No, only small cell clusters can be analyzed | Yes, allowing direct comparison of simulation results with readily determinable features (i.e., protein distributions obtained by flow cytometry) of populations in balanced or perturbed exponential growth |
| Mutant analysis | Yes, in principle, but no direct comparison with readily determinable population features is possible | Yes, allowing direct comparison of simulation results with readily determinable features (i.e., protein distributions obtained by flow cytometry) of populations in balanced or perturbed exponential growth |
| Open to plug-ins | Yes, in principle, but no direct comparison with readily determinable population features is possible | Yes, allowing direct comparison of simulation results with readily determinable features (i.e., protein distributions obtained by flow cytometry) of populations in balanced or perturbed exponential growth |

*R2.3	To my understanding, all of the heterogeneity in cell sizes between individual cells, which seems to play a key role in later analyses, seems to be coming from randomizing some model parameters in the GroCy model at every cell cycle. From my understanding of single cell heterogeneity, I would expect much of the differences in cell sizes must also come from differences in the growth rate lambda, but it doesn’t seem like the authors are adding any noises to the MeGro module of their model. I understand that mechanistically incorporating all sources of heterogeneity is something that is out of scope for this paper, but given that the “prediction” of cell size distributions seems to be a big selling point of this paper, I don’t think enough attention is being given to how the heterogeneity was achieved. The coefficient of variation the authors use (0.05) for the random sampling of GroCy, for example, should be part of Figure 2D – I would expect that outputs SD(P) and CV(P) are fairly sensitive to these very arbitrary parameters.*

**Answer:**

First, we would like to stress that in the integrated *iMeGroCy-2* population model, *λ* is not an input parameter but an emergent property of the simulation of the model itself. The complex structure of the yeast population, even in the absence of any added variability to timers and mother/daughter protein partition at division, originates a nontrivial variability in the mass duplication time (i.e., the time from birth to division). This heterogeneity – apparent between parents and daughters and within parent and, more so, daughter cells is mainly due to differences in the length of the G_1_ phase. In keeping with classic and more recent experimental results [Ref2-1; Ref2-2], the G_1_ length in our model is inversely correlated with the initial size. When we add variability at division, i.e., by setting a non-zero value for the Coefficient of variability (CV) applied to timers and protein partitioning at division, we observe a negligible difference in the MDT and G_1_ length of mothers and daughters (and a minor difference in the average protein content), but a significant increase in the standard deviation of these output parameters. These observations are summarized in the Table reported below, included in the **Supplementary Materials** as Table S6 of Section 5.c. We preferred to include this subject here rather than in the revised sensitivity map because this CV value would have been the only population parameter in a figure panel wholly dedicated to cell parameters.

**Supplementary Table S6 – Impact of variability in MDT and protein partitioning at division**

|  | Daughters | | | Parents | | |
| --- | --- | --- | --- | --- | --- | --- |
|  | Protein content  (average ± SD) | Cycle length (average ± SD) | G_1_ length  (average ± SD) | Protein content  (average ± SD) | Cycle length  (average ± SD) | G_1_ length  (average ± SD) |
| CV=0 | 4.03e10 ± 1.01e10 | 100.2 ± 3.1 | 15.2 ± 3.1 | 4.44e10 ± 1.03e10 | 96.3 ± 0.04 | 11.3 ± 0.04 |
| CV=0.05 | 4.28e10 ± 1.12e10 | 100.8 ± 7.1 | 15.7 ± 5.7 | 4.88e10 ± 1.18e10 | 96.7 ± 4.4 | 11.5 ± 1.25 |

The Reviewer correctly states that we do not add any noise in *MeGro*-2 parameters. In fact, *MeGro-2* is run only once for any given growth condition and its outputs fed as input parameters to *Grocy-2*. We do not change any of the internal parameters of *MeGro-2* when simulating different glucose concentrations and make only minor adjustments when modeling ethanol growth (see Supplementary for further details). For the glucose simulations, the outputs of the program only depend on the two input parameters, i.e., the glucose concentration and the corresponding respiratory ratio that we calculate from the following formula (Eq.(S21) in Supplementary Material)

$$F\approx20\frac{Y_{EtOH/glc}}{7+15Y_{EtOH/glc}}$$

using values determined from biochemical assays of glucose consumption and ethanol production in any growth condition (the *F* parameter has no meaning for ethanol simulation).

As the graph below shows, *MeGro-2* accurately predicts the growth rate *λ* in all the simulated conditions. This indicates that the parameter set used in *MeGro-2* captures growth rate modulations of yeast cells in various environmental conditions, which gives solid grounds for the robustness of this parameter set.

iMeGroCy2 λ (relative)

| Experimental λ (relative) | lambda |
| --- | --- |
| 0.493151 | 0.561644 |
| 0.808219 | 0.767123 |
| 0.876712 | 0.835616 |
| 0.917808 | 0.890411 |
| 0.958904 | 0.958904 |
| 1 | 1 |
| 0.986301 | 1.0137 |

**Supplementary Figure S2. Correlation between experimental and simulated values for the growth rate λ parameter**. Values reported are relative to the 2 % growth condition, taken as reference. The green bisector line indicates the ideal, perfect correlation between the two data series.

A sensitivity analysis of the *MeGro-2* model parameters reinforces this conclusion. We analyzed the effect of the variation of the catalytic coefficients *k_cat_* of the kinetic equations of the fluxes of *MeGro-2* on the primary output parameter, the exponential growth rate *λ*. The sensitivity analysis has been performed by fixing the *MeGro-2* inputs *F* and $c_{glc,ex}$ to the experimental values related to 2% glucose reported in the heading of Supplementary Table S.5. The results of this analysis are depicted in Supplementary Fig. S6 and reported below for convenience. Each panel shows the variations of the output parameter *λ* with respect to its value in 2% glucose when the nominal *k_cat_* (reported in Supplementary Table S.3) is varied of 0.01x, 0.1x, 10x, 100x. The analysis shows that only the catalytic coefficient *k_cat,gly_* has a sizable effect on the output parameter *λ*, that is later used to calculate the actual parameters fed to *GroCy-2* (see answer 4 below)

**Supplementary Figure S6 – Sensitivity analysis of the *MeGro-2* output *λ* with respect to *k_cat_* parameters** Relative ratios of *λ* (normalized with respect to the values in 2% glucose) as a function of the coefficient of variation of *k_cat,x_*, *x* ∈ *Prot2.*

*R2.4In Figure 3, the authors briefly describe their process of fitting parameters of their GroCy model such that the distributions of cell sizes match the experimental distributions for each media condition. Given the large number of parameters they were able to modify and the simplicity of the distributions, I am inclined to think that there must exist multiple combinations of parameter values that can achieve the same distributions and that the set of parameters that the authors have chosen is merely one of these many parameter sets. With a very limited discussion on their parameter fitting process, it is difficult to understand how the authors would argue that these parameter sets are the best sets of parameters. Looking at Table S3, it seems that very few of the parameters were adjusted in terms of GroCy inputs that do not come from MeGro. Could the authors provide justification for why they would think those specific parameters would be different depending on the environmental condition?*

**Answer:** The protein distribution may seem simple in shape, but it derives from complex biological relationships. Rather than brute force fitting of the protein distributions themselves, *iMeGrocy-2* aims to capture this complexity: its ability to provide simulated protein distributions that vary according to the nutritional and genetic conditions is the primary output feature of the model that is obtained through selected alteration of as few input parameters as possible. The Reviewer correctly highlights that, because of the large number of model parameters, different sets of values could provide the same satisfying fittings reported in old Fig.3, now Fig.4 (or even better). In this specific set of simulations, we feed to Grocy-2 some parameters derived from *MeGro-2*: one is *MeGro-2* output *ρ*, straightforwardly given as *GroCy-2* input parameter, the other are *K_2_* and τ*_2_*, constrained through Eq.(S.27) reported :

*λ = ρ*K_2_ - 1/*τ*_2_*

Infinite pairs (*K_2_* τανδ K*_2_*) exist that satisfy the constraint above, whose manifold is reported in the blue line in the figure below for the glucose 2% case. The five red markers refer to the pair of values chosen for the glucose 2% (the central marker) and for the other 4 choices of pairs (*K_2_,* τ*_2_*) satisfying constraint *λ = ρ*K_2_ - 1/*τ*_2_*, obtained by arbitrarily varying τ*_2_* as reported in the following Table.

Simulations results in the case of 2% glucose using parameter couples from Figure above show only negligible variations in population outputs.

| Glucose 2% | <P>, aa | MDT, min | <TG1>, min | <TG1D>, min | <TG1P>, min | Budding  Index |
| --- | --- | --- | --- | --- | --- | --- |
| τ*_2_* = 15000min  *K_2_* = 354.68 aa/rib/min | 4.46e10 | 99.24 | 14.30 | 16.70 | 11.81 | 0.81 |
| τ*_2_* = 6000min  *K_2_* = 359.56 aa/rib/min | 4.43e10 | 99.24 | 14.36 | 16.79 | 11.83 | 0.81 |
| τ*_2_* = 3000min  *K_2_* = 367.69 aa/rib/min | 4.57e10 | 99.29 | 14.34 | 16.78 | 11.81 | 0.81 |
| τ*_2_* = 1500min  *K_2_* = 383.95 aa/rib/min | 4.39e10 | 99.32 | 14.52 | 17.07 | 11.87 | 0.81 |
| τ*_2_* = 600min  *K_2_* = 432.73 aa/rib/min | 4.36e10 | 99.42 | 14.77 | 17.46 | 11.95 | 0.80 |

Dealing with the setting of the other *GroCy-2* parameters (the ones not related to *MeGro-2* outputs) in different nutrient environments, we decided to keep most values fixed to the ones of glucose 2%, letting a minimal set of model parameters to vary according to experimental evidence of literature. Actually, the richest nutritional environments (glucose 5%, 0.5%) did not need any further adjustment; regards to the poorest (glucose 0.2% 0.1%, 0.05% and ethanol 2%) it has to be stressed that, even if we had only changed the pair (*K_2_*_,_*ρ*) as coming from *MeGro-2*, we would have obtained a reasonable recap of the experimental features of the aforementioned conditions, as shown in the figure below and summarized in the Table below where main population output features are compared with or without further tuning.

**Supplementary Figure S4 –** Protein distributions for different low glucose and ethanolcon centrations, drawn for *GroCy-2* model parameters inherited from glucose 2%, without any further tuning than the one provided by the *MeGro-2* interconnection (green curves), with also a further (though minimal) tuning (blue curves), compared to the experimental ones (red curves). The grey shade refers to the glucose 2% experimental distribution reference.

|  | <P>, aa | SD, aa | CV | MDT, min | <TG1D>, min | <TG1P>, min |
| --- | --- | --- | --- | --- | --- | --- |
| Ethanol simulation from *iMeGroCy-2* tuned | 2.16e10 | 0.74e10 | 0.35 | 186.83 | 59.20 | 50.88 |
| Ethanol simulation from *iMeGroCy-2* not tuned | 1.77e10 | 0.68e10 | 0.39 | 189.26 | 196.92 | 39.62 |
|  |  |  |  |  |  |  |
| Glucose 0.05% simulation from *iMeGroCy-2* tuned | 2.40e10 | 0.70e10 | 0.29 | 132.01 | 56.91 | 29.06 |
| Glucose 0.05% simulation from *iMeGroCy-2* not tuned | 2.56e10 | 0.77e10 | 0.30 | 132.75 | 70.53 | 29.53 |
|  |  |  |  |  |  |  |
| Glucose 0.1% simulation from *iMeGroCy-2* tuned | 3.21e10 | 0.86e10 | 0.27 | 121.27 | 42.61 | 21.06 |
| Glucose 0.1% simulation from *iMeGroCy-2* not tuned | 2.93e10 | 0.82e10 | 0.28 | 122.30 | 52.16 | 24.34 |
|  |  |  |  |  |  |  |
| Glucose 0.2% simulation from *iMeGroCy-2* tuned | 3.74e10 | 0.94e10 | 0.25 | 112.46 | 29.82 | 15.51 |
| Glucose 0.2% simulation from *iMeGroCy-2* not tuned | 3.37e10 | 0.89e10 | 0.26 | 113.24 | 38.70 | 18.67 |

The *iMeGroCy-2* tuning procedure is now reported in all the details for both *MeGro-2* and *GroCy-2* in the Supplementary Material (Subsections 5.a and 5.b, respectively), with a specific care on the motivation that brought us to vary the few *GroCy* parameters in different nutritional environments. We briefly recap here the procedure adopted for the identification.

i) ***MeGro-2*.** Since *MeGro-2* is an extension of an available module of Metabolism & Growth published in [39], half of the module parameters are inherited from that reference, since they refer to pathways shared by both *MeGro* and *MeGro-2*. To avoid misunderstanding, we added a column to the *MeGro-2* input parameters table (renamed Table S5 in Supplementary Material) to clearly highlight what is taken from the literature and what is set by the authors. On the other hand, regards to *MeGro-2* parameters related to the new pathways not included in *MeGro* (i.e., the ethanol respiration and the gluconeogenic pathway producing pyruvate from ethanol), we follow two criteria for their tuning: (i) when glucose is the only external nutrient, *MeGro-2* must give the same outputs of *MeGro* (that can perform only simulations in glucose environment); this is obtained by making the transport of the produced internal ethanol more favorable than its recycling through the new pathways; (ii) when ethanol is the only external nutrient, *MeGro-2* must replicate the experimental data of our lab related to the growth rate of the wild type CEN.PK strain in an ethanol 2% (see Subsection 5.a of the Supplementary Material). We stress that the *MeGro-2* parameters do not vary with the nutritional environment; on the contrary, *MeGro-2* processes the nutritional availability providing different growth features according to different external conditions.
ii) ***GroCy*.** Dealing with the Growth & Cycle module, part of the growth parameters (ρ, *K_2_*) are provided by *MeGro-2* that senses different nutrients and straightforwardly provides different pairs of (ρ, *K_2_*). Concerning the remaining *GroCy* parameters, the ones related to glucose 2% environment are inherited from [39]; regards to the ones related to richer (glucose 5%) or poorer (glucose 0.5%, 0.2%, 0.1%, 0.05% and ethanol 2%) media we chose to keep them fixed to the values of glucose 2%, in the absence of experimental evidence suggesting a variation with respect to the nutrient. The only ones chosen to vary with respect to the reference media of glucose 2% are: the Far1 initial content (at cell birth, *Far1(0)*), the protein-over-volume ratio (*H*), Cln3-over-protein ratio (θ) and the parameters related to the three timers (i.e., T_1bmin_ and *W_0_* for T_1_ and the length of T_2_ and T_B_). Note that this is only a little subset of the whole set of *GroCy* parameters and initial conditions (7 out of 36) and no fine-tuning has been attempted for it. In particular, according to experimental data on the budded phase length, we roughly set T_B_ to three levels: low (85 min) for high glucose (5%, 2%, 0.5%), medium (90 min) for low glucose (0.2%, 0.1%, 0.05%) and high (133 min) for ethanol; concerning the remaining five parameters and *Far1(0)*, we coarsely adjust their values only for the poorest media, i.e., for ethanol 2% and for glucose 0.05% (in fact only *Far1(0)* and *W_0_* for glucose 0.05%), on the basis of experimental data on the temporal features. The Reviewer can find the details in the new Supplementary Material, Subsection 5.b.
iii) **Population.** A few rules govern the population simulations. They substantially refer to the rules of the asymmetrical division and to the noise affecting the setting of the timer lengths and the distribution of protein and ribosome contents in the newborn cells. These details can be found in the new Supplementary Material, Subsection 5.c.

In summary, the coarse setting of the input parameters mentioned above for the different nutritional environments, based on experimental or literature evidence, satisfactorily predicts the population outputs (protein distributions and temporal features) without a formal trial-and-error fitting procedure for the protein distribution. These concepts are also summarized in the main text, which is pasted below for your convenience.

First, we proved that our model could simulate different nutritional conditions using roughly the same parameters. As a first step, we run *MeGro-2,* varying its inputs (i.e., different glucose or ethanol concentrations and the fermentative-to-respiratory ratio), keeping all internal model parameters fixed at all nutritional conditions. Indeed, a change in the values of *K*_2_ and *ρ* alone (i.e., the *MeGro-2*-produced outputs) satisfactorily reproduces the output features of yeast populations grown in 5% and 0.5 % glucose (Fig. 4 C-E). For lower glucose concentrations, this approach captures the reduction in overall dimension and the decrease in growth rate. Still, it leaves space for further improvements, as shown for protein distributions in Supplementary Fig. S4. As explained in Supplementary Materials, a minimum of 2 and a maximum of 7 parameters (out of a total of 31) required minor adjustments from the values of the 2% condition. These changes were suggested mainly by experimental data (Supplementary Table S12A) and literature (see Subsection 5b in Supplementary Material for the details) and allowed the final distributions and corresponding outputs to be obtained, as reported in Fig.4 and Supplementary Table S7.

*R2.5	I have a similar comment for Figure 4. The authors provide very little discussion on what parameters they adjusted to simulate the two mutants, and how these adjustments were done. I can check Table S5 to see that adjustments were made on particular parameters in the MeGro model, which presumably led to changes in the growth rate, and one parameter from the GroCy model. Are these parameters relevant to the biological consequences of the mutations of these strains? Are there alternative parameter adjustments that could have achieved the same desired outcome? I am assuming that these parameters were chosen to be adjusted because they are relevant to the first steps of glucose metabolism, but this needs to be more explicitly explained. Also, it is unclear from the text how these parameters were refit for these mutations. The authors state that these parameters “comply with the mutant features and the outputs obtained in the simulations”. What mutant features and outputs are these? If these parameters were fit to match the experimental distribution of cell sizes, as was the case in Figure 3, the authors cannot state that their model “predicted” any of the values that are shown in Figures 4E through 4G*

**Answer:** As anticipated in the previous reply (R2.4) we have completely rewritten the Section on the setting of *iMeGroCy-2* parameters. Dealing with the mutants (Supplementary Material, Section 6), we aimed to show that *iMeGroCy-2* is able also to deal with genetic perturbations: such a task has been performed in a twofold way and the results of the double approach are reported in Figures 5 and 6. On the one hand, in Figure 5, we show that the model can predict the correct experimental behavior of mutants defective in the upper part of the glycolysis and glucose transport, by properly varying the correct parameters of the *MeGro-2* module. More in details, dealing with the mutants *hxk2 hxk1* and TM6* of Figure 4, we knew they are mutant defecting of the upper part of the glycolysis and of the glucose transport, respectively, therefore we suitably modified the specific parameters involved in the genetic alterations and find out how this focused parameter variation was able to reproduce the mutant population features. Indeed, the Reviewer was right saying that “*I am assuming that these parameters were chosen to be adjusted because they are relevant to the first steps of glucose metabolism”.* In this sense, we may say that our model was able to make predictions since, according to a very reduced set of model variations (biologically motivated by some *a priori* knowledge on the mutants) we have been able to qualitatively reproduce the alterations in the output populations of those mutants. The other way to treat mutants is dealt with in the next reply R2.6

*R2.6	Figure 5, again, there needs to be more discussion on which parameters they ended up altering, how the parameters are relevant to the biology of the mutations, and what model outputs they were pitted against.*

**Answer:** As anticipated in the answers R2.5, also the results reported in old Figure 5 deal with genetic perturbations, but in this figure, we played the opposite game to the one reported in Figure 4. In particular, in old Figure 5 (that has been reorganized in the new Figures 6 and Supplementary Fig. S12), we followed a reverse engineering approach to characterize the behavior of a pair of mutants defective in ribosome biogenesis (*rsa1*) and cell cycle (*whi5Δ and whi5^4E^*). In both cases, we exploited the experimental variations in growth and temporal population outputs with respect to the wild-type case to understand what parameter variations could be responsible for it. To this end, we exploited the heat map (redrawn and reported in new Fig.3D) as a tool to infer information when there is no *a priori* knowledge about what biological functions may be defective in specific mutants or the biological machinery is not modeled in detail in *iMeGroCy*-2 (as is the case for ribosome biosynthesis and cycle). The Reviewer may look at Subsection 6.c and 6.d for *rsa1* and *whi5*, respectively. However, in the revised version of the manuscript, we preferred to fuse in a unique figure (new Fig.6, see also the answer R2.8 for the motivation) the *whi5* mutant simulations with and without the molecular plug-in, leaving the *in silico* results on *rsa1* in the Supplementary Fig.S12.

*R2.7 The results that the glucose metabolism mutants and the rsa1 mutant, which is defective in ribosome biogenesis, have a significant reduction in protein synthesis rates does seem particularly interesting or valuable as a conclusion that is coming from a systems biology study that uses an integrated genome-wide computational model. Are there any nontrivial or emergent properties that arise from the completed model that are noteworthy?*

**Answer:**

As outlined above, the description of the *rsa1* mutant has been moved to the Supplementary. However, the discussion of the model outputs of different nutritional conditions and mutants and their highlighting of nontrivial and emergent properties has been extended in the Discussion. A relevant extract from the revised paper is reported below for convenience. Note that this discussion and a similar one on the cell cycle and Whi5 are presented in the frame of the ability of the integrated model to host molecularly models (see question R2.8)

**From Discussion**

Another foreseeable expansion of the integrated model could be to plug in a model of ribosome assembly – a very complex process involving more than 200 proteins and assembly factors [84] that could substitute the simple equations currently describing ribosome biosynthesis. In this framework, the coarse-grain model prediction of a defect in protein biosynthesis of the *rsa1* mutant (Supplementary Fig. S12), suggests that the Rsa1 protein is involved in a rate-limiting step in ribosome biosynthesis leading to a severe reduction of the protein synthesis rate [85]. Applying the same approach to other proteins involved in ribosome biosynthesis could thus contribute to validating the assembly process and identifying other regulatory steps that could not be disambiguated one from the other by simulating the coarse-grained model alone.

*R2.8	I don’t really understand what Figure 6 is trying to convey, except that the authors were able to replace a part of their GroCy module with a much more complicated sub-model and get pretty much the same outputs as the original version. Unless the authors were able to utilize this much complicated sub-model to do new types of analyses that work with the more fine-grained parameters, I don’t see the value in including this work in this manuscript.*

**Answer:**

The provided example is now described in revised **Figure 6,** which describes the analysis and interpretation of *whi5* mutants at both cellular and molecular levels. We use the figure as a proof-of-principle of the *iMeGrocy-2* model's ability to act as a scaffold for whole-cell models, where refined molecular models of a target molecular function can substitute one or more equations of the coarse-grain model. For this approach to work, the hybrid molecular model and the scaffold coarse-grain model simulations MUST provide the same output. This is explicitly mentioned in the first paragraph, where we describe the functions of the iMegrocy-2, and in the last chapter of the revised manuscript, where we discuss the utility of such an approach in relation to metabolism, cell cycle, and growth functions.

Thanks to the plug-in molecular details, we can properly select the kind of molecular players responsible for the apparent variations in the experimental outputs of the population: by suitably varying such parameters, we qualitatively reproduce the experimental data. We stress that such a task was not affordable without a molecular detail that explains the kind of genetic mutation. To stress the message and the biological insight of the use of the molecular plug-in, we have dedicated a unique figure to the whi5 mutants, with or without the plug-in.

**From Discussion** … while the coarse-grained model could only indicate the generic function affected in the mutants, the hybrid molecular model can describe the molecules involved in molecular terms. Note that modeling the ablation of the Whi5 protein or its substitution with a constitutively phosphorylated form can be tested individually, giving access to a broader range of simulations. Moreover, if actual interaction constants for wild type and mutant Whi5 should become experimentally available, the model could be improved, and the accuracy of the prediction tested.

…

Modelling system approaches may contribute to the extraction of the governing principles of microbial cell metabolism. Since different growth conditions result in sizeable metabolic rewiring ([58,64,65], the granularity level of the employed model critically affects the questions that can be asked.

…. As already done for the simpler organism *Mycoplasma genitalium* [33]*,* constraint-based metabolic models could be integrated with ODE-based models, and the allocation problem reformulated according to actual proteomic constraints.

*R2.9	 I was able to access the code for the model from the GitHub link that the authors provided, which contained detailed documentation for the general usage of the model, which I greatly appreciated. What I couldn’t find, however, were instructions on how I could reproduce the distributions of cell sizes that are shown in the figures presented in the manuscript. The version of MATLAB the authors used to generate the figures, as well as a clear list of commands that need to be run to reproduce the figures, would ideally be included in the documentation. I would also ask that the different parameter sets that are specific to each condition and mutant be included in a spreadsheet file that can be directly imported into the code, such that users would be able to quickly run simulations for all conditions and mutants presented in the manuscript. With the current version, it seems like users will need to manually replace the values in parameters.txt with the values provided in the supplementary tables, which substantially hurts the general accessibility of the model.*

**Answer:** We have rewritten the interface to insert the *iMeGroCy-2* parameters. Now, the user simply has to run the program and answer a short sequence of sentences asking what kind of simulation she/he wants to run. To this end, the user can choose among a set of 7 nutritional environments (the ones dealt with in the manuscript and whose input data are available from Supplementary Table S6) + 1 “neutral” environment where inputs can be manually given by the user from an Excel sheet. The whole program has now been rewritten in Python according to a parallel algorithm that dramatically reduces the computational burden, allowing the simulation of a population of 50 thousand cells in less than 15 minutes. Output data are now available both as raw data and as figures. We also added a MATLAB script to compare simulated protein distributions with the corresponding experimental ones. The MATLAB program implements the procedure described in the statistical analysis reported in the main text. In the MATLAB file folder, we added two folders containing the Python simulation results related to glucose 2% and glucose 0.05% and two MATLAB files (.mat) containing our experimental data related to the same nutrient conditions. These files can be used to test the system and may be substituted by user-provided ones.

R2.10. *Here are some of the more minor comments I had:*

*- There’s an unpaired parentheses in the last paragraph of the introduction.*

**Answer:** Fixed

R2.11. *The Figures are not referenced in the main text in the same order as they are presented (Fig 1AB -> Fig2 -> Fig1CD).*

**Answer:**

Fixed

R2.12. *The color palette used in some of the schematic figures (Figure 2A, 2B, 3A, 6A) are confusing and inconsistent. I would suggest using less colors in these figures overall, and clearly specifying what the different colors mean if they end up being used*

**Answer:** We thank the Reviewer for the suggestion. We limited as much as possible the use of color. In particular: in order to make the *iMeGroCy-2* scheme clearer, panels A and B of old Fig.2 are now fused into a unique panel in Fig.2 where colors are minimally exploited to denote the *MeGro-2* module (in red) and the *GroCy-2* module (in Orange), this last furtherly divided into the *Growth* module (in blue) and the *Cycle* module (in green). No other colors are necessary since, for instance, metabolites and proteins in *MeGro-2* are “shape-sensitive” (metabolites in ovals and proteins in rectangles). Fig.6A has been straightforwardly modified (it is Fig.6D in the revised version of the manuscript). Also, old Fig.3A (now Fig.3E in the revised version of the manuscript) has been reported without useless colors.

R2.13. *What is the “Yeast Genome Transcriptional Control” box supposed to represent in Figures 2A and 6A? Is this another module in the model? I don’t remember any mentions of this module in the main text.*

**Answer:** According to the Reviewer suggestion we have removed the “Yeast Genome Transcriptional Control”

R2.14. *For the heatmap in Figure 2D, the positive and negative directions should have entirely different color profiles (e.g. one in red, one in blue, 0% in white). With the current color profile it’s extremely difficult to see which parameters have positive or negative changes.*

***Answer:*** Thank you for the advice. The heatmap has been completely redrawn and should now be easier to follow. The new heatmap of Figure 3D depicts positive parameter variations with red shades and negative parameter variations with blue shades. The reference value (no variation) is indicated by light grey.

**References Reviewer #2**

[Ref2-1] (Johnston, Pringle, Hartwell. Coordination of growth with cell division in the yeast Saccharomyces cerevisiae. Exp Cell Res. 1977;105:79–98.

[Ref2-2] Brauer MJ, Huttenhower C, Airoldi EM, Rosenstein R, Matese JC, Gresham D, Boer VM, Troyanskaya OG, Botstein D. Coordination of growth rate, cell cycle, stress response, and metabolic activity in yeast. Mol Biol Cell. 2008 Jan;19(1):352-67.

**REVIEWER #3**

*Cell size homeostasis in budding yeast is well-studied under different carbon sources, including fermentable sugars and non-fermentative sources like ethanol. However, few models have integrated metabolism, growth, and cell cycle regulation. This manuscript expands on a previous computational model designed for glucose respiro-fermentation (iMeGroCyc, Ref. 39), which linked these processes. The key innovation of iMeGroCyc 2.0 (Figure 2) is the incorporation of ethanol energy production through respiration in the metabolism-to-growth module (MeGro) and the creation of a computational pipeline to simulate a yeast population (10^5 cells) undergoing balanced growth. This feature is essential for comparing the model with experimental data, such as flow cytometry, and for using these data to train and refine model parameters, as demonstrated in Figures 3-6. A significant finding of the manuscript is that cell size correlates with growth rate during fermentative growth but not during respiratory growth. This relationship is inferred from their fitted model (Fig. 3) and subsequently tested with experiments on fermentation-deficient mutant strains (Fig. 4). The authors further refine their model with ribosome-biogenesis mutations (rsa1) and cell cycle mutations (whi5_Delta and whi5^{4E}) in Figure 5. The final figure demonstrates that mechanistic molecular networks of the G1/S regulatory pathway can be added to the GroCyc module, allowing the Hybrid model (HyG1_S-iMeGroCy 2.0) to replicate cell cycle mutant data presented in Figure 5.*

*The strength of this new model (iMeGroCyc 2.0) lies in its ability to be directly compared with experimental data, which enables the model to be fitted and trained using flow cytometry data. Additionally, the model was tested and then refined with experimental data from various genetic mutants.*

R3.1. *However, I found the manuscript challenging to follow, mainly because the computational model's details are scattered throughout the main text, Methods section, and Supporting Information. The average PLoS Computational Biology reader would likely prefer all model details consolidated in one place.*

**Answer:**

Overall, the model's explanation and usage have been streamlined. The first two chapters of the **Results** summarize the structure of the integrated model (now called *iMeGroCy-2*), its components (*Megro-2* and *Grocy-2*), and their interconnection. They also discuss the significance of the input parameters and their robustness with respect to the output parameters. Finally, they briefly summarize the construction of the sensitivity analysis.

This part of the main text has been almost wholly rewritten compared to the original manuscript. We have pasted below the text and relevant figures for the reviewer's convenience.

**From Results**

**General structure and novel functionalities of the *iMeGroCy-2* model**

*iMeGroCy-2* is a substantial evolution of the previously published *iMegroCy* model [39] and consists of the interconnection of two modules: (i) a Metabolism & Growth (*MeGro-2*) and a Growth & Cycle (*GroCy-2*) module. *MeGro-2* includes lumped glycolytic and gluconeogenic pathways, a fermentation pathway, and a respiratory pathway, fed by either pyruvate or ethanol, a glucose transporter unit, bidirectional ethanol transport, and ribosomes devoted to biomass production. Ethanol derived from the outside can be respired or used in a simplified gluconeogenic pathway.

*MeGro-2* converts the available carbon source into energy (ATP) and macromolecules and uses a growth optimization algorithm to provide growth-related parameters to the *GroCy-2* module. Therefore, *MeGro-2* acts as a parameter generator, receiving two inputs for each growth condition: the external carbon source concentration ($c_{{glc}_{ex}}$ or $c_{{EtOH}_{ex}}$) and the fermentation ratio *F*, calculated from the experimental ethanol/glucose yield Y_EtOH_ for glucose-grown cells or set to 0 for ethanol-grown cells. Using these inputs, *MeGro-2* maximizes the exponential growth rate *λ*, accurately predicting it in different conditions that use as input different glucose concentrations (and their corresponding ethanol/glucose yield) or ethanol (Supplementary Fig. S2).

In *GroCy-2,* growth and cell cycle parameters not directly provided by *MeGro-2* are directly input by the user (see the Supplementary Material for the details on how *iMeGroCy-2* works and how its parameter values are set, Section 5). Cell growth is modeled by an Ordinary Differential Equation (ODE) system describing ribosome and protein dynamics (synthesis and degradation). A cyclin-mediated switch triggers the cell cycle, modeled as a series of consecutive timers. Timers *T_1a_*, *T_1b_*, and *T_2_* encompass the unbudded, G_1_ phase. *T_B_* corresponds to the budded phase, which includes the S, G_2_+M, and G_1*_ phases, of which the latter corresponds to the period that separates nuclear and cell division. The end of the last timer triggers cell division. The model incorporates a pedigree structure that follows the destiny of 5-10 cell clusters, each originating from a single founder cell (Fig.2), whose parameters are recorded. Cell-to-cell noise is introduced at cell division in newborn cells, giving rise to a simulated yeast population whose output temporal and cell growth parameters (notably protein distributions) can be directly compared with the equivalent parameters of experimental populations. Supplementary Fig. S5E presents a prototypical protein distribution generated by *iMeGroCy-2* for the case of 2% glucose in comparison with its corresponding experimental distribution. The Supplementary Materials file presents a detailed description of the integrated *iMeGroCy-2* model.

Tables 1 and 2 indicate why all computational simulations (and their comparison with experimental data) presented in Figures 4-6 *are only possible* with the new version of the integrated model. In fact: (i) only the revised *MeGro-2* supports growth in ethanol (see Supplementary Fig. S1 and accompanying text for a detailed comparison of *MeGro* and *MeGro-2)*; the presence of the population layer in *GroCy-2* is required (ii) to investigate how cell populations modulate their size according to different environments and/or genetic perturbations (which is the central biological insight of the manuscript) and (iii) to allow the model to act as a host for molecularly detailed modules, whose behavior can be studied within a context in which the general population dynamics are retained and act as constraints on the plugged-in module.

***iMegroCy-2* robustly describes the structure of exponentially growing yeast populations.**

To evaluate the robustness and biological significance of the identified parameter set, we first assessed the effect of each input parameter on the output growth and temporal parameters. Using the glucose 2% parameter set as a reference set, we conducted a sensitivity analysis by changing a single input parameter at a time while keeping the remaining input parameters constant. The monitored growth outputs are the average value of the protein distribution (<P>), its standard deviation (SD(P)), and coefficient of variation (CV(P)). The monitored temporal outputs are the mass duplication time of the population (MDT) and the length of the G_1_ phase of parent and daughter cells (T_G1D_ and T_G1P_, respectively). Each input parameter has been moved "forward" and "backward" compared to its nominal value. For each parameter setting (resulting from each parameter variation), the exponential growth of the yeast population was simulated, linking the *GroCy* input parameters to the quantitative features of the cell population. Supplementary Material, Subsection 5.d. provides details on the procedure. Figures 3A-C report an example of the sensitivity analysis for input parameter *T_2_*. Supplementary Figures S7-10 show the complete results of the sensitivity analysis summarized as a heat map in Fig. 3D. The heat map shows that the perturbation of many input parameters has limited or no effect on the tested outputs, indicating that the model is robust against parameter alterations. At the same time, the map provides suggestions on which parameters could be worth changing to obtain a simulated cell population more closely resembling an experimental yeast population perturbed by chemical or genetic means, thereby hinting at the cellular functions altered by the perturbation (see later chapters for examples).

**Figure 1.** **Cell cycle and cell size in yeast**

(A-B)	Schematic view of the cell cycle with cycle times for parents (P) and daughters (D) with different genealogical ages; T_G1_ and T_B_ are the lengths of the unbudded (G_1_) and budded (S+G_2_+M) phases, respectively.

(B) Cell size (protein content distribution obtained by flow cytometry).

**Figure 2.** **Structure and utilization of the integrated Metabolism Growth and Cycle (*iMeGroCy-2*) model**

Upper part: *iMeGroCy-2* (purple box) modules interconnections. *MeGro-2* (red box) and *GroCy-2* (orange box), with the *Growth* (blue box) and *Cycle* (green box) modules included in *GroCy-2*. Black and grey arrows inside *MeGro-2* box denote metabolic conversions and protein synthesis, respectively, whilst rectangles and ovals denote protein classes and metabolites, respectively.

Lower part: *GroCy-2* also incorporates a population pedigree framework following the destiny of germinal lines originated from distinct single progenitor cells. Parent (P, grey) and daughter (D, white) cells of different genealogical ages (as defined by the subscript numbers) are depicted in the pedigree map (lower part of the cartoon).

**Figure 3.** **Sensitivity analysis**

(A-C)	Sensitivity analysis with respect to T_2_ (glucose 2%). (A): simulated protein distributions drawn for different values of T_2_ and compared to the experimental one related to 2% glucose. (B): protein features (average and initial cell protein content, critical cell size and size at division) extracted from the simulated populations of panel A, endowed with their standard deviations. (C): G_1_ phase length for the whole population and for the subpopulations of daughters and parents, related to the simulated populations of panel A, endowed with their standard deviations.

(D)	Heat map describing the alterations in major output growth and temporal parameters obtained by changing the input parameters of the *GroCy-2* module. The considered temporal parameters are the mass duplication time of the population (MDT) and the G_1_ phase lengths of parent (T_G1Par_) and daughter cells (T_G1Dau_). The growth parameters are the average value (P), standard deviation (SD(P)) and coefficient of variation (CV(P)) of the cellular protein content.

(E)	Flow chart of the identification procedure for mutational and environmental perturbations

The procedures followed for analysis of the different growth conditions or the mutants are now better detailed in the Supplementary Materials and summarized in the Main text. By way of example, the following text is pasted from the third chapter of the revised manuscript:

***Metabolism drives the coordination between cell growth and division***

First, we proved that our model could simulate different nutritional conditions using roughly the same parameters. As a first step, we run *MeGro-2,* varying its inputs (i.e., different glucose or ethanol concentrations and the fermentative-to-respiratory ratio), keeping all internal model parameters fixed at all nutritional conditions. Indeed, a change in the values of *K*_2_ and *ρ* alone (i.e., the *MeGro-2*-produced outputs) satisfactorily reproduces the output features of yeast populations grown in 5% and 0.5 % glucose (Fig. 4 C-E). For lower glucose concentrations, this approach captures the reduction in overall dimension and the decrease in growth rate. Still, it leaves space for further improvements, as shown for protein distributions in Supplementary Fig. S4. As explained in Supplementary Materials, a minimum of 2 and a maximum of 7 parameters (out of a total of 31) required minor adjustments from the values of the 2% condition. These changes were suggested mainly by experimental data (Supplementary Table S12A) and literature (see Subsection 5b in Supplementary Material for the details) and allowed the final distributions and corresponding outputs to be obtained, as reported in Fig.4 and Supplementary Table S7.

The new **Supplementary Material**, whose structure is reported below for the reviewer's convenience, details the integrated mathematical model, **its significance** and setting of input parameters, and the simulation of mutant populations.

1) ***iMeGroCy-2* in a nutshell**
2) ***iMeGroCy-2 vs iMeGroCy***
3) ***The MeGro2 module***
   a. *The optimization problem associated to pure glucose environment (*$c_{{EtOH}_{ex}}=0$*)*
   b. *The optimization problem associated to pure ethanol environment (*$c_{{glc}_{ex}}=0$*)*
4) ***The GroCy-2 module***
   a. *Asymmetrical division and genealogical age heterogeneity*
5) ***Working of iMeGroCy-2 and parameter setting***
   a. *MeGro-2 parameter setting*
   b. *GroCy-2 parameter setting*
   c. *Simulations of cell populations through iMeGroCy-2*
   d. *Sensitivity analysis*
   e. *Simulations of cell populations through Hy-iMeGroCy-2*
6) ***Simulation of mutants populations***
   a. *Mutant hxk1 hxk2*
   b. *Mutant TM6**
   c. *Mutant rsa1*
   d. *Mutants whi5^Δ^ and whi5^4E^ (iMeGroCy-2 simulations)*
7) ***Additional experimental Tables and Figures***
R3.2. *Major comments:*

*** Figure 1 feels disjointed and lacks coordination with the main text. The authors use Fig. 1A-B to discuss population size heterogeneity (mother vs. daughter, generations), while Fig. 1C-D presents experimental data on ethanol production and respiro-fermentation. It might make more sense to integrate Fig. 1A-B with the modeling in Fig. 2, whereas Fig. 1C-D could be moved to the Supporting Information or potentially combined with Fig. 3, which fits the model to size distributions across different glucose and ethanol concentrations.*

**Answer:** We have extensivelyrearranged the Figures have integrated panels A and B of old Fig.1 with the scheme of the model (now panel C in new Fig.1). We have straightforwardly moved panels C and D of old Fig.1 to new Fig.4

R3.3. *** The predictive model offers the advantage of providing deeper insights. A key result of this manuscript is that cell size scales with growth rate during fermentative growth but not during respiratory growth. I couldn't find a straightforward explanation or modeling insight for this phenomenon. Could you please elaborate on the underlying reason for this key difference in cell size scaling with growth rate between fermentation and respiration in your manuscript.*

**Answer:**

We have rearranged the discussion—also in response to another reviewer's question—and expanded it. *The relevant extract from the novel Discussion is reported below for convenience.*

Fast-growing cells (bacteria, fungi, and mammals) often favor fermentative metabolism instead of respiration to generate ATP under nutrient-rich conditions, even when oxygen is available (aerobic glycolysis: Crabtree and Warburg effect). Rather than “wasteful”, such a metabolic strategy is considered a trade-off between energy yield and protein costs to maximize growth [55,56]. In fact, despite the lower ATP yield of fermentation relative to respiration, glycolytic enzymes have been reported to have a superior ATP synthesis capacity per enzyme mass than respiratory enzymes. As a result, fermentation has been proposed to produce energy faster and at significantly lower proteomic costs than respiration [55–63]. Therefore, forcing respiration under nutrient-rich conditions (such as in the TM6* and *hxk2 hxk1* mutants) may prevent the optimal allocation of limited proteomic resources, severely limiting the capacity of yeast cells to maximize growth and modulate their size. Adopting fermentation under nutrient-rich conditions may allow yeast cells to allocate a more significant fraction of the limited proteome resource to activities required to maximize their growth rate (including synthesis of new ribosomes), and possibly to modulate their size. In keeping with this view, our model correctly predicts that forcing respiratory metabolism in glucose-rich media reduces the protein synthesis rate, slow growth, and loss of cell size modulation, as observed in the non-fermenting mutants.

Recent findings, however, indicate that respiration is more proteome efficient than fermentation and that respiratory yeasts (such as *I. orientalis)* can grow faster than glycolytic yeasts like *S. cerevisiae* [64]. The authors postulate that fermentation evolved to enhance robustness under poor oxygen availability or hypoxia conditions. Since cells are relatively inefficient at metabolic proteome remodeling, adopting a fermentative metabolism that works well under aerobic and oxygen-limited environmental conditions would be more advantageous.

*MeGro-2* does not make specific assumptions regarding the proteome efficiency of respiration *vs.* fermentation but uses an optimization algorithm to allocate resources to different cellular tasks, including the production of ATP and macromolecules required to sustain growth, such as ribosomes and enzymes for glucose transport, glycolysis, respiratory and fermentative pathways*,* its performance being constrained by the concentration of the available carbon source and its mode of utilization. The regulatory connections within *GroCy-2* then produce the integrated model's emergent outputs. Modelling system approaches may contribute to the extraction of the governing principles of microbial cell metabolism. Since different growth conditions result in sizeable metabolic rewiring ([58,64,65], the granularity level of the employed model critically affects the questions that can be asked. Genome-wide computational models of yeast metabolism describe the salient features of yeast growth, which are modeled as an increase in total biomass [37,66]. These models excel in describing steady-state situations. However, despite some promising attempts [36], they do not provide the best framework for integrating metabolism with macromolecular synthesis, cell cycle, and division. Because complex biological functions, which may involve hundreds or thousands of gene products, arise as emergent properties of a network [67,68] and not merely as the sum of the activities of all proteins involved in that network, dynamic molecular models are required to understand how a genotype interacts with the surrounding environment to produce a given phenotype [69–72]. Resource allocation optimization can be performed at the genome-scale, as was recently described for *E. coli* [73], and applied to central metabolism in yeast under the simplifying condition that flux and protein levels are coupled [58,63], as experimentally observed in *E. coli* [74]. As already done for the simpler organism *Mycoplasma genitalium* [33]*,* constraint-based metabolic models could be integrated with ODE-based models, and the allocation problem reformulated according to actual proteomic constraints.

R3.4. *Minor comments:*

*** The model is an updated version of iMeGroCyc in Ref. 39. Why not call it iMeGroCyc 2.0?*

**Answer:** We agree with the Reviewer; we call it *iMeGroCy-2* throughout the paper.

R3.5. *** The justification and details of the sensitivity analysis for Figure 2D are unclear to me. The criteria seem arbitrary (for example, why separate parameter variation into positive and negative classes?), and the explanations are vague (such as how much the parameters were varied). Could you please clarify? One suggestion might be to create a stand-alone main figure that combines Fig. S3 (an example of T_2 sensitivity analysis) with Fig. 2D (sensitivity analysis results for all parameters).*

**Answer:** We thank the Reviewer for the comments. The original version of the manuscript was lacking a clear development of the sensitivity analysis. In the revised **Supplementary Material** we provide all the details of the sensitivity analysis and motivation for the use of different input parameter variations. An example of the simulation run to construct the heat map is presented As Fig 3A-C, and all the original data are provided as Supplementary Figures S7-S10 within section 5.4 of the **Supplementary**.

Figure 3 was pasted previously for the convenience of the reviewer

R3.6. *** I am not sure if the model currently supports glucose and ethanol as dynamic variables. My assumption is that it does not, and that both experimental work and modeling are carried out under conditions of logarithmic growth where nutrients remain mostly constant. For future models, it would be valuable to incorporate factors like storage carbohydrates, secreted metabolites, and the interplay between the cell cycle and metabolism. This could provide deeper and testable insights into the origin of yeast metabolic rhythms observed in respiration and fermentation under specific bioreactor conditions.*

**Answer:** The Reviewer correctly highlights that our model does not account for nutrient variation. We thank the Reviewer for the suggestion about future models: indeed, it is a work in progress.

R3.7. *** Typo at bottom of page 11. "As shown in Fig. 5 ..." should be "As shown in Fig. 4 ..."*

**Answer:** Figure numbering has changed. The correspondence between citation and actual Figures and Table has been double checked.
