# Supplementary material for: A modular model integrating metabolism, growth, and cell cycle predicts that fermentation is required to modulate cell size in yeast populations
Source: PLoS Comput Biol. 2025 Jul 21;21(7):e1013296. doi: 10.1371/journal.pcbi.1013296 (PMC12377586; doi:10.1371/journal.pcbi.1013296)
Supplement: Attachment — Submitted filename: Answer_Reviewer_1.docx [file pcbi.1013296.s005.docx]

Reviewer #1:
While I acknowledge the additional work you have incorporated, I must express that my core concerns remain unresolved. It is still unclear what the primary aim of this study is and what its main findings are. While the manuscript builds upon your previous iMeGroCy model, which is not inherently an issue, it does not introduce new results or insights that significantly go beyond the capabilities of the earlier model. Specifically, while iMeGroCy-2 incorporates the ability to model growth on ethanol and adds a population layer, the fundamental question remains: what novel understanding does this model provide that was not accessible with the previous version? The manuscript should more clearly articulate its core contribution beyond these modifications.

***Answer in brief***

*iMeGroCy-2* explicitly analyzes the exponential growth of yeast populations in liquid media. This method of growing yeast cells encompasses not only the growth of shaken flasks but also growth in fermenters and chemostats, which are *significant* growth conditions in both basic science and applied fields, as yeast that produces valuable products, such as metabolites or proteins, is cultivated in this manner (Shi & Li, 2025).

*Considering that*

- protein distributions are accurate descriptors of the physiological state of yeast population growing in liquid media;
- the ability to describe growth in ethanol-supplemented media is unique to the novel *iMeGroCy*-2 model; the original model (*iMeGroCy)* CANNOT be used to analyze protein distributions and the population structure inherently produced by *iMeGroCy-2* is necessary to extract information from experimental protein distributions properly,
- in this paper we use protein distributions as the principal biological readout to study yeast populations growing in different media and with different genotype

*we conclude that*

the revised *iMeGroCy-2* model introduces significant novel elements compared to its predecessor and enables analyses that the original *iMeGroCy* does not allow.

During the revision process of this paper, a study was published that draws inspiration from our work and further strengthens our hypothesis that the rate of glucose metabolism sets cell size in many yeast species (Li et al, 2025).

***Novel content included in the paper***

*Enhanced explanation of protein distributions (extracted from revised* ***Introduction****)*

The protein distribution (i.e., the frequency of cells in a population with a given protein content), measured *via* FITC staining and flow cytometry, provides a quantitative proxy for cellular growth and division status. It is closely linked to the age distribution, which relates to the “actual” state of population growth and depends on the growth law of an individual cell, i.e., the law of protein accumulation during a cell cycle. Protein distributions indicate how a population is growing at the time of analysis and thus provide an accurate snapshot of the current physiological state of a yeast population (Porro *et al*, 2009). For instance, protein distributions differ in cell populations that are exponentially growing on different carbon sources (Vanoni *et al*, 1983) or as cells approach and reach the stationary phase due to glucose exhaustion and later resume growth by using ethanol as a carbon source (Alberghina *et al*, 1983). Experimental distributions differ from theoretical ones due to biological variability and instrumental noise, which must be considered in modeling efforts. When considering biological and instrumental noise, these theoretical distributions change in shape and closely resemble experimental distributions (Fig. 1B; (Alberghina *et al*, 1983)).

In line with the criteria proposed in (Tummler & Klipp, 2024), we extend a previously developed coarse-grained model (*iMeGroCy*) that integrates metabolism, ribosome biogenesis, protein synthesis, and cell cycle progression in an average yeast cell (Palumbo *et al*, 2018). The updated model (*iMeGroCy-2*) incorporates population structure, enabling direct comparison with experimental protein distributions obtained by flow cytometry, used as the principal biological readout to study yeast populations growing in different media and with different genotypes. Additionally, the model now includes ethanol metabolism, broadening its applicability to different nutrient conditions.

**Revised Figure 1** **Cell cycle and cell size in yeast**

(A)	Schematic view of the cell cycle for parents (P) and daughters (D) with different genealogical ages. T_G1_ and T_B_ are the duration of the unbudded (G_1_) and budded (S+G_2_+M) phases, respectively.

(B)	Size distribution of a yeast cell population. The red line is the theoretical intracellular protein content density function (adapted from Alberghina et al., 1983). The blue line is the fitting of experimental protein content distribution (obtained by flow cytometry) with “protein density functions with variability” originated by theoretical function. The lower strip schematically depicts how cells of different genealogical ages but with similar size distribute along the size profile.

*Enhanced textual and graphic explanation of the revised* iMeGroCy-2 *model structure and wiring (extracted from revised* ***Results****)*

*iMeGroCy-2* (Fig. 2) is a significant evolution of the previously published *iMeGroCy* model (Palumbo *et al*, 2018)*.* In summary, the integrated model identifies three functions: *(i)* *Metabolism, (ii) Cell growth, and (iii) Cell cycle and division.* The interconnection of two modules, a Metabolism & Growth (*MeGro-2*) module and a Growth & Cycle (*GroCy-2*) module, allows the modeling of the complete biological cycle of yeast populations (Fig. 2).

*(i) Metabolism*: Through a resource allocation algorithm, *MeGro-2* feeds parameters (the desired RNA/protein ratio and the rate of protein synthesis per ribosome) to a dynamic module (*GroCy-2*). *MeGro-2* includes ……..

*(ii) Cell Growth - i.e., cell mass accumulation -* and *(iii)* *Cell cycle and division*: In *GroCy-2,* growth and cell cycle parameters not directly provided by *MeGro-2* are directly input by the user ……

…… The *GroCy-2* module is simulated for each cell. Starting from one or more founder cells, the population structure is dynamically constructed over the course of the simulation (Fig. 2).

…… Tables 1 and 2 show why all computational simulations (and their comparison with experimental data) presented in Figures 4-8 *are only feasible* with the new version of the integrated model. Notably, the population structure that emerges from the *GroCy-2* simulation *is essential* for analyzing and extracting biological information from protein distributions (see **Introduction** for more details) obtained during the exponential growth of yeast populations in liquid media. This method of growing yeast cells encompasses not only the growth of shaken flasks but also growth in fermenters and chemostats, *significant* growth conditions in both basic science and applied fields, as yeast that produces valuable products, such as metabolites or proteins, is cultivated in this manner (Shi & Li, 2025). The population structure is also a prerequisite for using the model as a host for molecularly detailed modules, whose behavior can be studied within a context in which the general population dynamics are retained and act as constraints on the plugged-in module. Finally, only the revised *MeGro-2* supports growth in ethanol (see Supplementary Fig. S1 and accompanying text for a detailed comparison of *MeGro* and *MeGro-2*).

**Revised Figure 2.** **Structure of the integrated Metabolism Growth and Cycle (*iMeGroCy-2*) model.**

The *iMeGroCy-2* model (purple box) and its modules’ interconnections are depicted, including *MeGro-2* (red box) and *GroCy-2* (orange box), which incorporates the *Growth* (blue box) and *Cycle* (green box) modules. Black and grey arrows inside *MeGro-2* box denote metabolic conversions and protein synthesis, respectively, while rectangles and ovals denote classes of proteins and metabolites, respectively. The *GroCy-2* module simulates a population of several thousand cells, starting from 10 germinal lines, each originating from 10 distinct single progenitor cells. Parent (P) and daughter (D) cells of varying genealogical ages (as defined by their subscript numbers) are illustrated here in the pedigree map. Similar to real-life populations, these cells are linked by a Parent-Daughter connection. As each cell is simulated according to *GroCy-2* , represented by the *GroCy-2* thumbnail inside the cell, the population structure forms as an emergent property of the overall population. The dice symbolize the “random noise” introduced at each division event in newborn cells. *iMeGroCy-2* yields population temporal and growth parameters. An example plot showing the temporal outputs for a 50k population of daughter and parents cells is here reported. The growth outputs histogram plot shows how *iMeGroCy-2* groups together cells of different genealogical ages but similar sizes (i.e. protein content) within a population. A typical cell size distribution plot is also displayed (blue line graph).

*Enhanced analysis of the relevance of modeling cell populations (extracted from* ***Discussion****)*

An emerging trend in cell modeling is moving toward multi-scale integration, where individual cell behavior is modeled with molecular precision, allowing researchers to simulate and predict population-level phenomena with high fidelity. (Skalnik *et al*, 2023) introduced a multi-scale modeling framework that extends their prior whole-cell model of *E. coli* to the colony level. By embedding individual cell models within a shared dynamic environment, the framework captures both intracellular processes and collective colony behavior. Applied to antibiotic resistance -a major public health concern driven in part by phenotypic heterogeneity- the model enabled detailed simulation of *E. coli* responses to tetracycline and ampicillin, two antibiotics with distinct mechanisms of action, thus bridging molecular and population scales. *SimulCell* is an agent-based simulator for eukaryotic cell proliferation, migration, and division on flat substrates, grounded in time-lapse microscopy data (Toscano *et al*, 2025). Similar to *SimulCell*, our model captures individual cell proliferation and variability. However, *iMeGroCy* goes beyond mere rule-based approaches, as it leverages a low-granularity whole-cell algorithm, allowing proliferation to emerge from molecular-level interactions. By incorporating higher-resolution molecular models (see later for a fuller discussion), our model offers cell-specific molecular insights comparable to those of (Skalnik *et al*, 2023).

***More content relevant to the subject that could not fit in the paper***

Protein distributions (i.e., the frequency of cells in a population with a given protein content) are obtained through FITC staining of the total protein content in a cell. The protein distribution is closely linked to the age distribution, which relates to the “actual” state of population growth and depends on the growth law of an individual cell, i.e., the law of protein accumulation during a cell cycle. Protein distributions indicate how a population is growing at the time of analysis and thus provide an accurate snapshot of the current physiological state of a yeast population (Porro *et al*, 2009). The ability of protein distributions to reflect changes in the environment is shown in **Fig. Ref1-1** below (taken from (Alberghina *et al*, 1983) that shows how protein distributions change as cells approach and reach the stationary phase because of glucose exhaustion and later recover growth by using ethanol as a carbon source. *In this paper we use protein distributions as the principal biological readout to study yeast populations growing in different media and with different genotype*.

**Fig.Ref1-1** Left: Growth curve for a yeast culture from the inoculation (t = 0) to the resumption of growth in ethanol after glucose exhaustion (post-diauxic shift). Right: Experimental protein distributions of yeast cells sampled at different hours after inoculation: (A) 3 h; (B) 6 h; (C) 15 h; **(D)**16 h; (E) 18 h; **(F)** 21 h; (G) 23 h; (H) 25 h; (I) 28 h. Solid lines represent protein distribution of mid-exponential phase (12 h). Taken from (Alberghina et al, 1983).

The left panels of **Fig.Ref1-2** show the theoretical protein distributions that originate from three different assumptions: **A)** parent (or daughter) cells are born at the same mass regardless of genealogical age; **B)** the mass of newborn parent cells increases linearly with genealogical age; **C)** the mass of newborn parent cells increases with genealogical age according to a saturation function. Situation **C** closely describes the experimental situation and corresponds substantially to the assumptions we used in this manuscript.

These theoretical distributions differ significantly from the actual protein distributions obtained by flow cytometry. When considering biological and instrumental noise, these theoretical distributions can be utilized to fit experimental distributions, as shown in the right panels of **Fig.Ref1-2**.

**Fig. Ref1-2** Left panels. Theoretical protein density functions for alternative growth models (I-III) of yeast. Right panels. Fitting of experimental yeast protein distributions with *protein density functions with variability* obtained by theoretical functions reported in the left panels. Taken from (Alberghina *et al*, 1983).

We may now ask to what extent and with what accuracy the original *iMeGroCy* model could analyze the data reported in Figure 4 of the first revision of the manuscript.

Single-cell simulations are the only simulations in (Palumbo *et al*, 2018), in which *MeGro* was able to handle only the 2% glucose condition. An analysis conducted for all glucose conditions (but not for ethanol, a nutritional condition that we cannot simulate following the pipeline *MeGro* to *GroCy* in the original iMeGroCy) would provide the length of the cell cycle but would not reproduce the average protein content. We could approximate the average protein content in some ways, for instance, by using the protein content of a simulated cell after 50% of the cycle has elapsed. This would produce Figure **Ref1-4A** below. Even if we do not consider the variability inherent in the complex yeast population structure, some cell-to-cell variability is expected. If we run 10 different simulations, considering an initial protein content variability of 5% and a CV of 10% in the Timer lengths (i.e., the same variability utilized in the population simulations of our manuscript), we obtain Fig. **Ref1-4B**. Clearly, this population-free structure does not allow for proper analysis of experimental protein distributions.

We can take an additional step by increasing the number of generic simulated cells to 50,000, applying the same variability described above. **Figure Ref1-4** panels **C** and **D** present simulation results for the two extreme glucose cases: 2% and 0.05% glucose. Now, we observe smoother size distributions, yet the overall fitting remains poor. Not surprisingly, when we explicitly incorporate parent cells into the simulation, there is a significant improvement, particularly in the 2% case (**Figure Ref1-4** panel **E)**. The 0.05% simulation (Figure **Ref1-4** panel **F**) accurately reflects the overall reduction in size but does not account for the distribution's shape correctly. The simulated distributions fit the experimental ones only when we consider the appropriate pedigree structure of *iMeGroCy-2~~.0~~*, which, as noted earlier, derives from the simulations themselves (**Figure Ref1-4** panels **G and H**).

**Fig. Ref1-4** – **A**: Experimental protein distribution of CEN.PK 2% glucose *vs* single cell simulation. **B**: Experimental protein distribution of CEN.PK 2% glucose *vs* 10 single cell simulations. **C**: Experimental protein distribution of CEN.PK 2% glucose *vs* simulated population of daughters without pedigree in 2% glucose. **D**: Experimental protein distribution of CEN.PK 0.05% glucose *vs* simulated population of daughters without pedigree in 0.05% glucose; **E**: Experimental protein distribution of CEN.PK 2% glucose *vs* simulated population of daughters and parents without pedigree in 2% glucose; **F**: Experimental protein distribution of CEN.PK 0.05% glucose *vs* simulated population of daughters and parents without pedigree in 0.05% glucose; **G**: Experimental protein distribution of CEN.PK 2% glucose *vs* simulated pedigree population in 2% glucose; **H**: Experimental protein distribution of CEN.PK 0.05% glucose *vs* simulated pedigree population in 0.05% glucose.

Pedigree simulations introduce additional degrees of freedom, such as variability in timer durations and protein/ribosome partitioning at division. In the first revised version of the manuscript, we explored how these coefficients of variation influence key population-level features (old **Supplementary Table S6**, now substituted by a more complete sensitivity analysis Supplementary Fig. S12) In the present revision, we leverage these pedigree-level population parameters (NOT single-cell model parameters) to improve the model's prediction of the *rsa1* mutant phenotypes - specifically by increasing the coefficient of variation in protein partitioning at division: this parameter is not directly associated to any single cell parameter, being inherently related to the pedigree population. Indeed, by doubling the CV of protein/ribosome partitioning at division (and keeping all previous model parameter variations as suggested in Supplementary Table S13), we obtain a simulated protein distribution that better describes the mutant distribution (compare the blue (improved) and green (original) distributions, as reported in **Fig. Ref1-3**).

**Fig. Ref1-3** – Experimental and simulated protein distributions for the *rsa1* mutant. The green line (sim A) is the old distribution already present in the previous version of the manuscript; the blue line (sim B) is the new distribution reported in the current version of the manuscript, obtained varying the CV of protein/ribosome partitioning at division.

We will now answer specific points raised by the reviewer.

1. The updated iMeGroCy-2 model extends the previous model by incorporating ethanol metabolism and a population layer. However, the manuscript struggles to demonstrate how these additions lead to significant new biological insights. For example, the model's prediction that fermentation is required for cell size modulation is interesting, but a more in-depth exploration of the underlying molecular mechanisms would strengthen the manuscript's impact.

***Answer in brief***

- We will present some general considerations on the predictive depth of different resolution models in response to points 4 and 5.
- We performed a more in-depth analysis of the role of fermentation (F-ratio) in cell size modulation, along with comparisons to literature data. We now present the results of *MeGro-2* simulations, which were not included in previous manuscript versions, and a broader selection of results from the *GroCy-2* simulations. Figure 5 of the first revision of the manuscript only included results regarding the average protein content. We now include simulation results concerning all the output parameters presented in the sensitivity analysis.

***Novel content included in the paper***

*Enhanced description of the role of fermentation in cell mass modulation at different glucose concentrations (extracted from revised* ***Results****)*

In glucose-limited continuous cultures, cell size has been consistently shown to be small and relatively constant at low growth rates when only respiration is active. In contrast, it increases steeply at faster growth rates when cells switch to fermentation, leading to ethanol production (Johnston *et al*, 1979; Porro *et al*, 2003). Furthermore, cell size increases even at low growth rates if the cells are forced to shift from respiratory to fermentative metabolism (Porro *et al*, 2003). Data presented in Fig. 4 and in Supplementary Fig. S17, S19 and Table S1 confirmed that glucose can modulate cell size in a dose-dependent manner as a function of the growth rate: increasingly higher sugar concentrations in the growth medium support faster growth rates and larger sizes (Supplementary Fig.S17, Supplementary Table S14A) (Porro *et al*, 2003; Youk & van Oudenaarden, 2009; Soma *et al*, 2014; Schmidt-Glenewinkel & Barkai, 2014). The linear correlation between the yield of ethanol and the protein content of the population grown at different glucose concentrations is consistent with the notion that the type of energy metabolism (respiratory, respiro-fermentative, fermentative) influences both cell growth rate and size (Supplementary Fig. S19E). We first tested whether the outputs of the metabolism-related module *MeGro-2* were consistent with *in vivo* proteome allocations reported in published datasets (Paulo *et al*, 2016) for yeast cells grown under comparable conditions: namely, 2% glucose and fully respiratory, non-fermentable carbon sources (pyruvate, acetate, glycerol).

Proteins were assigned to five classes according to their physiological functions (“glycolysis”, “fermentation”, “*HXTs*” (glucose carriers), “respiration”, “ribosome/translation”) and the relative abundance of each group was calculated from experimental data (Figure 5A, right side; see also Supplementary Fig. S18 and Supplementary File). Although acetate, glycerol, and pyruvate (and ethanol, not used in) are metabolized via partially different metabolic pathways, they all converge on mitochondrial respiration, ultimately sustaining ATP production. Therefore, the values for the “fully respiratory” condition in Fig. 5 represent the average of the allocation values obtained for acetate, glycerol, and pyruvate.

The left part of Figure 5A shows the *MeGro*-2-predicted proteome allocations for the same experimental conditions described in Figure 4. *MeGro-2* predictions for both standard (2% glucose) and fully respiratory conditions (*i.e.,* ethanol) align well with the experimental trends: compared with the reference 2% glucose condition, the respiratory pathway investment significantly rises under fully respiratory conditions, whereas translation-related proteins strongly decreases and glycolytic/fermentation enzymes show a moderate reduction (Fig. 5A). *MeGro-2* predicts a steady, smooth increase in respiratory allocation from 0.5% to 0.05% glucose and a sharp rise for ethanol (full respiration). The increased abundance in respiratory enzymes is accompanied by a parallel decrease in the fraction of fermentation enzymes. *MeGro-2* predicts a reduction in glycolytic enzymes only for the lowest glucose concentrations (< 0.2%) and (above all) for ethanol (Fig. 5A). Moreover, exclusively for ethanol, *MeGro-2* also predicts a significantly reduced allocation in ribosomal synthesis machinery (Fig. 5A), which matches the dramatic change in growth rate observed when moving from 0.05% glucose to ethanol, both *in silico* and *in vivo* (Fig. 4K; Supplementary Table 14A; Supplementary Fig. S18). The close agreement between *MeGro-2*-simulated and experimental growth rate values is confirmed in Fig. 5B.

Next, we analyzed protein allocation as a function of the fermentative ratio (F) across glucose concentrations (Figs. 5C–H). The allocation changes from full respiration (F = 0) to full fermentation (F = 1) closely mirror those in Panel 5A, suggesting that shifts in protein allocation are primarily driven by the F-ratio, rather than by external glucose itself.

While *MeGro-2* does not explicitly model biomass accumulation (*i.e.,* RNA and protein synthesis) and cell division, *GroCy-2* incorporates both functions. Fig. 6 summarizes the *GroCy-2* simulation outputs for growth and temporal parameters obtained by systematically varying the F-ratio at different glucose concentrations, *i.e.,* the same conditions analyzed in Fig. 5 for the *MeGro-2* outputs. Panel 6A reports the average protein content of the population. The dose-dependent modulation of cell size by external glucose (estimated by the average protein level of the population, P) is substantially diminished for F values ≤ 0.8, with protein content remaining uniformly low regardless of the external glucose concentration. Similar heatmap profiles appear with both the Standard Deviation (SD(P): Fig. 6B) and the Coefficient of Variation (CV(P), i.e., SD(P)/P: Fig. 6C). Both values remain constant until glucose metabolism is significantly shifted toward fermentation.

Temporal features, including Mean Doubling Time (MDT: Fig. 6D) and the G_1_-phase length of both parent (Fig. 6E) and daughter cells (Fig. 6F), are also influenced by the F-ratio. Even under enforced respiration, only minimal modulation of the temporal parameters is observed at F ≤ 0.6, becoming more pronounced at higher F values. When respiration prevails, MDT and G_1_ length increase significantly in low-glucose conditions, while for F > 0.6, MDT correlates with glucose levels.

**Revised Figure 5. Proteome allocation and growth rate predicted by *MeGro-2*.**

(A) Proteome allocation for the CEN.PK strain under various nutritional conditions simulated by *MeGro-2*. As indicated, the model considers five protein classes (HXTs (glucose carriers), glycolysis, fermentation, respiration, and ribosome/translation), which *in vivo* account for approximately 40% of the entire proteome (according to YeastGenome.org). (Ho *et al*, 2018)). The experimental values reported were derived from the literature. ((Paulo *et al*, 2016): see Fig. S18; only the five aforementioned classes were considered for the final computation). Since allocation data for ethanol growth conditions were not available, the average value of three non-fermentable carbon sources (glycerol, acetate, and pyruvate) is shown here.
(B) Correlation between the experimental λ growth rate values and values simulated by *MeGro-2*.

(C-H)	MeGro-Simulated proteome allocations under different fermentative conditions (F ratio between [0, 1]) in various growth media (0.05% to 5% glucose). In each panel, we reported as reference the simulated values for the CEN.PK strain and the experimental literature data (same as panel A).

**Revised Figure 6. Modulation of population growth and temporal parameters under various fermentative conditions across different growth media**

*GroCy-2* was used to simulate the growth of yeast populations under various fermentative conditions (F ratio ranging from [0, 1]) across different growth media (spanning from 0.05% to 5% glucose). The model input parameters were set according to the values for the wild-type CEN.PK strain reported in Table S5. All output parameter values are displayed as heatmaps. The “CEN.PK” column on the right side of each heatmap presents the simulated output parameter values obtained from *iMeGroCy-2* by setting the F ratios equal to the experimentally measured values for the CEN.PK strain cultivated in the respective growth medium.

(A-C) Growth parameters: P, SD(P) and (CV(P) of the intracellular protein content

(D-E) Temporal parameters: MDT, T_G1P_ and T_G1D_

*Discussion of the role of fermentation in cell mass modulation at different glucose concentrations (extracted from revised* ***Discussion****)*

*MeGro-2* simulations indicate that nutrient-dependent cell size modulation requires glucose fermentation. *MeGro-2* allocates resources across five core biosynthetic processes: glucose transport, glycolysis, fermentation, respiration, and translation—based on a growth-optimizing algorithm (Fig. 5). Under predominantly respiratory conditions (F-Ratio ≤ 0.8), protein allocation remains largely unchanged (Fig. 5), and *GroCy-2* predicts modest shifts in temporal dynamics across glucose concentrations (Fig. 6). At higher F-Ratios, a significant reallocation from respiratory to fermentative enzymes occurs (Fig. 5C–H). Ribosomal and translational protein levels are stable in glucose but decline in ethanol, consistent with reduced growth rates in ethanol and in 0.05% glucose, a near-fully respiratory condition (Fig. 5B).

Under prevailing fermentative conditions (F-Ratio > 0.8), the growth rate (λ) becomes glucose-dependent. Although *GroCy-2* adopts *MeGro-2*-derived parameters, it computes λ independently as an emergent property of population-level dynamics. Model predictions correlate closely with experimental measurements (Fig. 5B, Supplementary Fig. S5). Elevated glucose accelerates growth, advances Cln3 accumulation, and shortens G_1_ duration. Since the budded phase remains relatively constant, faster-growing cells divide at larger sizes, producing larger newborns and increasing protein distribution heterogeneity, while mean doubling time and G_1_ duration decline (Fig. 6,).

Empirical data show that translation-related proteins in yeast scale linearly with growth rate, whereas glycolytic enzymes and chaperones decrease under respiratory conditions (Fig. 5A; Supplementary Fig. S18; (Xia *et al*, 2022; Metzl-Raz *et al*, 2017; Björkeroth *et al*, 2020; Paulo *et al*, 2016). Fast-growing cells (bacteria, fungi, and mammals) often favor fermentative metabolism instead of respiration to generate ATP under nutrient-rich conditions, even in oxygen-rich environments (aerobic glycolysis: Crabtree and Warburg effect (Alberghina, 2023)). Rather than “wasteful”, such a metabolic strategy is considered a trade-off between energy yield and protein costs to maximize growth (Molenaar *et al*, 2009; Goel *et al*, 2012). In fact, despite the lower ATP yield of fermentation relative to respiration, glycolytic enzymes have been reported to have a superior ATP synthesis capacity per enzyme mass than respiratory enzymes. As a result, fermentation has been proposed to produce energy faster and at significantly lower proteomic costs than respiration (Molenaar *et al*, 2009; Goel *et al*, 2012; Basan *et al*, 2015; Nilsson & Nielsen, 2016; Chen & Nielsen, 2019; Mori *et al*, 2019; Flamholz *et al*, 2013; Pfeiffer *et al*, 2001; Elsemman *et al*, 2022). Consistent with this view, *MeGro-2* predicts that forcing respiration under nutrient-rich conditions may prevent the optimal allocation of limited proteomic resources, severely limiting the capacity of yeast cells to maximize growth and modulate their size according to the available carbon source. Experimental validation supports model predictions: the TM6* and *hxk1 hxk2* mutants, locked into a mostly respiratory metabolism even in glucose media, exhibited slow growth, reduced protein synthesis and no cell size modulation (Fig. 7). Therefore, fermentation in nutrient-rich environments likely facilitates the optimal proteome allocation required for growth and size control.

This view has been challenged by recent findings suggesting that respiration may actually offer superior proteomic efficiency than fermentation, allowing several respiratory yeasts to grow faster and outcompete the glycolytic ones under certain conditions (Shen *et al*, 2024). The study postulate that fermentation may instead have evolved as a robust adaptation to hypoxic environments: since cells are relatively inefficient at metabolic proteome remodeling, adopting a fermentative metabolism that works well under both aerobic and oxygen-limited conditions would be more advantageous. While *MeGro-2* does not impose intrinsic efficiency differences between metabolic modes, it allocates proteome resources optimally based on the availability of carbon sources. *GroCy-2* incorporates these dynamics to generate emergent, system-level behaviors that allow for estimations of protein distributions, average protein content, and intra-population protein content variability.

**Revised Fig.S5 Correlation between growth rate λ values obtained from *MeGro-2* and *iMeGroCy-2*.** *MeGro-2* and *iMeGroCy-2* were used to obtain the growth rate λ values under different fermentative conditions (F ratio between [0, 1]) in various growth media (from 0.05% to 5% glucose).

The model's ability to reproduce experimental data, while important for validation, does not, in itself, constitute a novel finding.

*We hope that the new material and a more in-depth description of results and implications present in the revised paper and discussed above, may convince the reviewer that the paper not only provides a valuable tool but also employs it to yield novel findings.*

In more detail:

The ability of the simulated distributions to fit the experimental ones under various environmental and genetic conditions with minimal tuning of input parameters—guided by experimental evidence and biological reasoning—indicates that:

- The general architecture of the *iMeGroCy-2* model, where metabolism serves as the key driver of growth and the cell cycle, is not speculative but grounded in experimental evidence. This revision provides more detail than previous ones in describing the biological plausibility of our parameter choices and the relevance of the integrated population structure to the model's predictive depth.
- The *iMeGroCy-2* model can effectively serve as a scaffold for models of biological functions with increasing molecular resolution. This is both novel and not trivial, as it represents the first attempt to implement a TOP-DOWN approach to whole-cell modeling—complementary to the BOTTOM-UP approach used mainly until now.
- The ability to describe the altered properties of protein distribution resulting from genetic mutations suggests that the *iMeGroCy-2* model captures the essential functions modified in the mutants. For instance, a change in the timer function can explain the altered protein distributions in the *whi5Δ* mutant. The model's predictions regarding changes in protein synthesis—verified experimentally—are not immediately evident consequences of the timer mechanism's alteration but are emergent properties readily identified through simulation.
- The prediction's level of resolution depends on the model’s granularity, such that the basic *iMeGroCy-2* model can only identify the timer as the affected function. However, when integrating a molecular-level model of the G1/S transition, resolution can be enhanced, and the faulty timer function can be pinpointed as a defect in the Whi5 protein, which may either be absent (*whi5Δ* mutant) or derive from constitutively pseudo-phosphorylated Whi54E protein.
- The integrated prediction of protein allocation and population structure—new to this paper revision—is unprecedented in the literature.

2. The manuscript heavily relies on outdated references, with minimal integration of recent and impactful studies that are highly relevant to the field of whole cell modeling. This not only limits its relevance but also fails to adequately situate the research within the current advancements in the field. The discussion section of the manuscript could be strengthened by a more thorough survey of the recent literature. This is particularly relevant given the increasing sophistication of whole-cell models and the integration of omics data.

***Answer in brief***

We completely rewrote the relevant paragraphs of the Discussion and enlarged the discussion of genome-wide metabolic models (GEMs). Being limited to metabolic reactions, we did not consider GEMs as full-fledged whole-cell models.

The new version of the Discussion:

- includes some more detail and newer references on GEMs and their constraining by post-genomic and thermodynamic data.
- cites more papers on specific yeast cellular functions (reviewed in (Tummler & Klipp, 2024)).
- includes reference to the newly proposed concept of Artificial Intelligence Virtual Cells (AIVCs), which aim to integrate AI and omics data to obtain powerful digital replicas of cellular functions.

***Novel content included in the paper***

*Enhanced description of Whole cell models with newer references and wider discussion of genome -wide metabolic models (extracted from revised* ***Discussion****).* The pasted Discussion piece integrates also the role of blown-up models, relevant for the answer to points **4** and **5 (Text in green)**.

A whole-cell computational model is designed to simulate the complete behavior of a living cell by integrating the complex interactions among cellular components like proteins, metabolites, genes, and regulatory networks, aiming to offer an integrated, systems-level perspective of cellular functions, such as metabolism, signaling, and gene expression (reviewed in (Tummler & Klipp, 2024)). To date, only a handful of whole-cell models are available, since developing and curating these kinds of models is challenging and labor-intensive (Kaizu & Takahashi, 2023; Georgouli *et al*, 2023; Zhong *et al*, 2022). The first whole-cell model was developed for *Mycoplasma genitalium* a human urogenital parasite whose biological functions were modelled by 28 interacting modules (Karr *et al*, 2012). A fully dynamic, 3D spatial whole-cell model of a synthetic minimal bacterium with a reduced genome of 493 genes, a number close to the 525 genes of *M. genitalium*, has also been published (Thornburg *et al*, 2022). Several refined versions of the original whole-cell model of *E. coli* (Macklin *et al*, 2020), whose genome contains more than 4000 genes (https://ecocyc.org/ECOLI/organism-summary) have been developed (Ahn-Horst *et al*, 2022; Sun *et al*, 2021; Choi & Covert, 2023). The current model includes 43% of known gene products that collectively simulate key cellular processes (including metabolism, DNA replication, transcription and translation (Sun *et al*, 2021; Ahn-Horst *et al*, 2022)) and has been recently extended, as mentioned above, to a population-scale, producing a “whole-colony” model (Skalnik *et al*, 2023).

The granularity level of any model critically affects the questions that can be asked*.* Constraint-based models can describe the salient features of yeast growth, which is, however, modeled simply as an increase in total biomass without accounting for macromolecular syntheses (Kerkhoven, 2022; Lu *et al*, 2022). The genome-scale metabolic model (GEM) of yeast metabolism has been continuously refined and enhanced since the initial publication (Förster *et al*, 2003), culminating in the recently published *Yeast9* (Zhang *et al*, 2024). The integration of GEMs with COnstraint-Based Reconstruction and Analysis (COBRA) methods, including Flux Balance Analysis (FBA), has yielded extensive insights into the core structure and functional dynamics of metabolic pathways (Kerkhoven, 2022; Georgouli *et al*, 2023; O’Brien *et al*, 2015). GEMs can also be constrained by incorporating enzyme reaction thermodynamics (*k_cat_*), post-genomic data (including transcriptomics, proteomics, metabolomics and fluxomics), subcellular compartmentalization and energetic costs of biological processes (see for instance (Kerkhoven, 2022; Lu *et al*, 2022; Sánchez *et al*, 2017; Oftadeh *et al*, 2021; Chen *et al*, 2022)), significantly enhancing the predictive capabilities of classical GEMs.

In principle, Genome-scale metabolic models (GEMs) may also provide a solid basis for building increasingly complex multiscale models by incorporating modules of different cellular processes (Tummler & Klipp, 2024; Elsemman *et al*, 2022; Sánchez *et al*, 2017): the development of the WM_S288C computational model is an important first step towards a comprehensive whole-cell model of yeast, that can be used to decipher the complex relations between genotype and phenotype (Ye *et al*, 2020), although significant challenges remain unsolved (Lu *et al*, 2022; Tummler & Klipp, 2024; Elsemman *et al*, 2022).

While established whole-cell models adopt a bottom-up approach, reconstructing cellular complexity from individual components to achieve high-resolution descriptions, *iMeGroCy-2* employs a top-down strategy. It models the core functions of eukaryotic cells—metabolism, growth, and the cell cycle—at low granularity, functionally interlinked to capture emergent cellular behaviors.

The modular design of *iMeGroCy-2* enables substitution of coarse-grained components with increasingly detailed molecular modules. Each major module – or part of it - can be replaced or refined into interconnected submodules, preserving functional behavior and facilitating efficient parameter tuning. This plug-in framework ensures simulation consistency between high- and low-resolution modules and allows resolution choice based on the biological question under investigation. To validate this scaffold approach, we integrated an existing molecular model of the G_1_/S transition (Palumbo *et al*, 2016) into the Cell Cycle module. The resulting hybrid model (Fig. 8D) accurately replicated growth dynamics, timing, and protein distributions for both wild-type and mutant strains, matching results obtained with the coarse-grained model (Fig. 8A–B; Supplementary Fig. S15). While both versions predict decreased protein content and synthesis rates in mutants, the hybrid model offers mechanistic insights by identifying specific molecular alterations. For example, simulations of *whi5* deletion or its constitutively phosphorylated mutant (*whi5^4E^*) reproduced expected phenotypes. While the coarse-grained model can only suggest which macro-functions are affected in mutants, the hybrid molecular model enables detailed analysis of the specific molecular players involved. Simulations of either *whi5* or *whi5^4E^* mutations can be performed independently, expanding the model’s utility. In the case of *whi5^4E^*, the model not only captures the expected shift in protein distribution toward lower values, but also suggests an alteration in the interaction constants between SBF and Whi5^4E^. Refinement of these parameters—pending experimental determination—would enhance the model’s accuracy and predictive capability.

These results confirm the model's utility as a scaffold for integrating mechanistic modules, capable of capturing mutant phenotypes and guiding further refinements. Foreseeable expansions of the integrated model could include both the description of cell growth and metabolism. A model of ribosome assembly – a highly complex process involving more than 200 proteins and assembly factors (Klinge & Woolford, 2019) could replace the simple equations currently describing ribosome biosynthesis. In this context, the coarse-grained model's prediction of impaired protein synthesis in the *rsa1-*null mutant (Supplementary Fig. S16) suggests the involvement of Rsa1 in a limiting step of ribosome biogenesis, leading to a severe reduction of the protein synthesis rate (Kressler *et al*, 1999). Incorporating detailed models of ribosome assembly could validate these findings and reveal regulatory interactions unresolvable at lower resolutions.

Constraint-based models can be integrated with ODE-based frameworks, enabling reformulation of the allocation problem based on measured proteomic constraints. Recent work has demonstrated genome-scale resource allocation optimization in E. coli (O’Brien *et al*, 2013) and applied similar strategies to yeast central metabolism under the assumption of coupled flux and protein levels (Elsemman *et al*, 2022; Nilsson & Nielsen, 2016). Such an approach would allow for effective integration of (multi-) omic data, as discussed above. As shown for Mycoplasma genitalium (Karr *et al*, 2012) and more recently for *E. coli* (Macklin *et al*, 2020; Sun *et al*, 2021) and a living minimal cell (Thornburg *et al*, 2022), constraint-based models can be integrated with ODE-based frameworks, enabling reformulation of the allocation problem based on measured proteomic constraints.

Although a full list of molecular models describing specific yeast cellular functions (reviewed in (Tummler & Klipp, 2024) pluggable in *iMeGroCy-2* goes beyond the scope of this paper, it is worth citing models simulating processes like cells cycle (Münzner *et al*, 2019), DNA replication (Brümmer *et al*, 2010; Spiesser *et al*, 2010), transcription (Amoussouvi *et al*, 2018) and translation (Seeger *et al*, 2023). The online availability of a Yeast Cell Model Data Base (YCMDB, [https://www.tbp-klipp.science/ycmdb/](https://www.tbp-klipp.science/ycmdb/)) may be instrumental in collecting and providing curated, high quality data for model testing and validation.

In conclusion, our coarse-grained algorithm functionally integrates the core macro-processes of proliferating cells—metabolism, growth, and the cell cycle—each governed by thousands of gene products. The sensitivity analysis (Fig. 3D; Supplementary Figs. S8–S11) enables the identification of macro-functions disrupted by environmental or genetic perturbations. The model’s modular structure supports the stepwise integration of detailed molecular modules and subsystem models, whose development can be enhanced through AI-based methodologies (Yu *et al*, 2016). This allows mechanistic models to be quantitatively evaluated within a validated framework reflecting the genetic and environmental influences on yeast populations. Crucially, the model bridges a key gap by linking molecular mechanisms to population-level behaviors—growth, cell cycle progression, and environmental responses—features often absent in isolated molecular models. Given the evolutionary conservation of core proliferative mechanisms from yeast to humans, this framework holds promise for application in mammalian systems. It offers a foundation for understanding deregulated growth in cancer and developing targeted therapies.

Despite advances, current models fall short in capturing the full complexity of living cells. This underscores the need for more integrated approaches that combine omics data with computational models. The emerging concept of Artificial Intelligence Virtual Cells (AIVCs) proposes a promising avenue, leveraging AI to create comprehensive digital twins of cellular functions (Qian *et al*, 2025; Bunne *et al*, 2024).

3. While the model's parameter fitting process is now detailed in the supplementary material, it still relies on a trial-and-error approach with a very limited tuning set of parameters. The model’s robustness is analyzed with a sensitivity analysis. However, more justification is required to address the following:

(a) How do you ensure the parameters you selected are the most biologically relevant?

The parameterization of the two *iMeGroCy-2* modules, *MeGro-2* (with 27 input parameters, see also Supplementary Table S3) and *GroCy*-2 (with 27 input parameters, see also Supplementary Table S3), is initially dependent on biological constraints and can be subsequently fine-tuned through sensitivity analysis. Together, these steps ensure biological plausibility and robustness of the chosen parameter set under each environmental and genetic condition. See Supplementary Information (Section 5a) and the Discussion for further information and in depth discussion (piece taken from revised **Results**).

(b) How do you address the possibility that other parameter sets could also reproduce the same experimental data?

***Answer in brief***

This is a relevant point. We have expanded our single-parameter sensitivity analysis to include an analysis that examines the co-variation of multiple parameters simultaneously. The results of this analysis are presented in the revised **Supplementary Materials** and discussed in the context of sloppy and stiff parameters in the revised **Discussion**. Parameter sloppiness implies that different combinations of parameters may yield similar phenotypic outputs when parameters are allowed to co-vary in sloppy directions (see, e.g., the cloud of simulations with varying combinations of parameter values (Supplementary Fig. S13, reported below) which generate nearly superimposable protein distributions, (Supplementary Fig. S14, reported below). Conversely, there are parameters - for instance, *ρ* and *K_2_ –* belonging to stiff directions whose variations consistently shift the output populations to a much higher protein content (supplementary Fig. S13, reported below).

***Novel content included in the paper***

*Description of sensitivity performed by co-variation of multiple parameters (extracted from the revised* ***Supplementary****)*

To further investigate the robustness of the simulations with respect to a selected set of parameter values, we analyzed what occurs by systematically and simultaneously varying a small set of *GroCy-2* parameters. A thorough analysis would involve in a systematic way the whole parameter space, i.e. a set of 20 parameters. Even by assuming a minimal set of 3 values for each (namely, the nominal value, a proper increase and a proper decrease) we would have 3^20^ (i.e. more than 3 billion) cases. As a matter of fact, such an investigation of the model robustness would have gone out of the scope of the manuscript, therefore we resorted in investigating what happens by varying four sets of selected 5 input parameters. Each set includes parameters from the growth module, the molecular interplay between Cln3 and Far1 and the Timer setting. More in details:

- Set 1: K_1_ (*10, /10), τ_1_ (*10, /10), k_on_ (*5, /5), W_1_ (*1.001, /1.001), T_2_ (*1.2, /1.2)
- Set 2: ρ (*1.1, /1.1), τ_2_ (*1.1, /1.1), k_off_ (*5, /5), W_1_ (*1.001, /1.001), T_2_ (*1.2, /1.2)
- Set 3: K_2_ (*1.1, /1.1), τ_1_ (*1.1, /1.1), k_nc_ (*5, /5), W_0_ (*1.001, /1.001), T_2_ (*1.2, /1.2)
- Set 4: K_1_ (*10, /10), ρ (*1.1, /1.1), Far1(0) (*1.2, /1.2), W_1_ (*1.001, /1.001), T_2_ (*1.2, /1.2)

Each parameter is allowed to vary up and down of the same average amount chosen for the single parameter sensitivity map reported in Fig. 3D. That means, each set of 5-tuple provides 3^5^ = 243 populations (including the one without any parameter variation). For each parameter set we computed the same six outputs exploited to build the sensitivity heatmap, i.e. the average protein content with its standard deviation and coefficient of variation, the Mass Duplication Time, the average G1 phase for both Daughters and Parents. All these simulations refer to the glucose 2% nutritional environment.

Due to the importance of protein distribution as a fingerprint for each growth condition, Supplementary Fig. S13 illustrates the average protein content and its standard deviation for each simulation on an XY plane, where each point corresponds to a different simulation, color-coded according to the 5-tuple set. This graph offers a partial yet comprehensive representation of the entire set of simulations. The simulation results provided by the four 5-tuples clusters in two regions. Most simulations (approximately 80%) form a tight cluster with relatively low average protein content and standard deviation (Cluster Low), while a less populated cluster (Cluster High, about 20%) displays significantly higher values for both outputs.

**Supplementary Figure S13. Clustering of virtual populations as coming from multi-parametric sensitivity analysis**. Each point refers to a population. Different sets are reported according to different colors. Besides, the 7 simulated nutritional environments are reported, as well as 4 mutants discussed later.

**Cluster Low** encompasses most nutritional conditions, except for the two poorest ones, i.e., 0.05% glucose and 2% ethanol, which present an even smaller protein content position: the protein content range spans from approximately 3 to 6x10^10^ (with the nominal protein content being around 4.5x10^10^). Supplementary Table S8 reports, for completeness, the set of multiparametric variations providing the different nutritional conditions and some mutants discussed later. No such variations involve a significant increase in *ρ* or in *K_2_* (indeed, all these populations are in Cluster Low, or in lower positions). We recall that the different nutritional conditions in Cluster Low have largely been addressed automatically by the model without the action of the modeler, because of the interplay between *MeGro-2* and *GroCy-2*, the former automatically tuning the growth parameters (*ρ, K_2_*) fed to the latter, according to its embedded optimization program. By getting poorer and poorer nutritional conditions, the *MeGro-2*/*GroCy-2* automatic adjustments are no longer sufficient, see Subsection 5.b for details.

**Supplementary Table S8. Set of multiparametric variations providing *in silico* different nutritional conditions and mutants**

|  | ρ | K_2_ | Far1(0) | k_on_ | H | Θ | W_0_ | W_1_ | T_2_ | T_B_ |
| --- | --- | --- | --- | --- | --- | --- | --- | --- | --- | --- |
| Glc 5% | *1.0005 | *1.0119 | - | - | - | - | - | - | - | - |
| Glc 0.5% | /1.0043 | /1.0473 | - | - | - | - | - | - | - | - |
| Glc 0.2% | /1.0166 | /1.1063 | - | - | - | - | - | - | - | *1.0588 |
| Glc 0.1% | /1.0342 | /1.1593 | - | - | - | - | - | - | - | *1.0588 |
| Glc 0.05% | /1.0611 | /1.2088 | /1.4118 | - | - | - | /1.0047 | - | - | *1.0588 |
| EtOH 2% | /2.5027 | *1.4327 | /2.1818 | - | /1.1472 | /1.1353 | /1.0252 | - | *3.5 | *1.5647 |
| TM6* | /1.0525 | /1.2252 | - | - | - | - | - | - | - | /1.0759 |
| *hxk2 hxk1* | /1.0493 | /1.3732 | - | - | - | - | - | - | - | *1.1765 |
| *rsa1* | /1.4408 | - | - | *2 | - | - | - | *1.0032 | /2.5 | *1.1765 |
| *whi5* | - | - | - | - | - | - | - | *1.0121 | - | - |

To obtain further details about populations in **Cluster Low**, we analyzed the proximity to the nominal Glucose 2% population based on a distance that considers all six outputs reported in the heatmap. To this end, we utilized the *L_∞_* norm, which provides the maximum percentage displacement among the six outputs. We found 67 populations that have a distance smaller than 5% from the reference population grown in 2% glucose and thus can be considered approximately equivalent to the nominal case.

Among these 67 populations:

- 19 are obtained by varying just 1 parameter. These cases come straightforward from the sensitivity heatmap where we investigated what happened by varying one parameter at a time
- 27 are obtained by varying 2 parameters
- 17 are obtained by varying 3 parameters
- 4 are obtained by varying 4 parameters
- No populations are obtained by varying all 5 parameters

Within these 67 simulated populations, we identified those with protein distributions closest to the nominal one by evaluating the Mean Square Error (MSE) between each distribution and the nominal one. The results show that the closest populations (MSE<50) are:

1) Set 1, 2 varied parameters (τ_1_*10, W_1_*1.001)
2) Set 1, 2 varied parameters (τ_1_/10, k_on_*5)
3) Set 1, 3 varied parameters (τ_1_/10, k_on_*5, W_1_*0.999)
4) Set 2, just 1 varied parameter (k_off_/5)
5) Set 2, 2 varied parameters (τ_2_*0.9091, k_off_*5)
6) Set 2, 3 varied parameters (τ_2_*0.9091, k_off_*5, W_1_*0.999)

Whilst the less close (among the closest, MSE>150) are

7) Set 1, 4 varied parameters (K_1_*10, τ_1_*10, k_on_/5, W_1_*1.001)
8) Set 2, 3 varied parameters (τ_2_*1.1, k_off_*5, W_1_*1.001)
9) Set 3, 2 varied parameters (k_nc_/5, W_0_*0.999)
10) Set 3, 2 varied parameters (τ_1_*1.1, W_0_*0.999)
11) Set 3, 3 varied parameters (τ_1_*1.1, k_nc_/5, W_0_*0.999)

The Supplementary Figure S14 below presents three of the best protein distributions (items 2, 4, 5) and one of the least accurate ones (item 10) in comparison to the nominal case (light cyan shadow). While the three best protein distributions (red, blue, magenta lines) are indeed superimposable with the nominal case, the less closely simulated population (green line) exhibits a protein distribution that starts to separate significantly from the nominal case. Therefore, this particular case provides a reference context that may help in judging the extent to which a specific data set captures the experimental protein distribution.

Item 10 (Set 3)

Item 5 (Set 2)

Item 4 (Set2)

Item 2 (Set 1)

Nominal

**Supplementary Figure S14.** Protein distributions from Cluster Low. Comparison of the protein distributions of 4 simulated populations among the 67 ones that have a distance (*L_∞_* norm) smaller than 5% from the reference population (nominal parameters used for WT 2% glucose) compared to the nominal one: red, blue, magenta distributions have a MSE < 50 while the green one has a MSE > 150 w.r.t. the nominal distribution.

Even the smallest average protein values of simulations of **Cluster High** are almost 70% larger than the 5% conditions. Literature data [13,14] indicate that *large* mutants have a size (mean or median volume) that is about twice that of the wild type, likely setting an upper limit for haploid cells. Since the mean volume of diploid cells is about 70%-100% larger than haploid cells [11,12], and cells with higher ploidy (at least up to 4n) roughly increase their size proportionality, populations with an average protein content larger than 10^11^ might represent cells with a diploid DNA content or higher. It is worth noting that achieving an average protein distribution in the **Cluster High** relies significantly on the parameters *ρ* and *K_2_*, whose slight modifications provide considerable variations of the chosen outputs: indeed (i) Set 1 (which does not change *ρ* and *K_2_*) does not exhibit any such case, while (ii) Sets 2-4, which provide populations in **Cluster High**, all demonstrate an increase in *ρ* or *K_2_*. The following Supplementary Table S9 reports the kind of parameter variations and the number of simulated populations belonging to the **Cluster High**.

**Supplementary Table S9. Set of multiparametric variations providing *in silico* populations in Cluster High**

| **Simulation set** | **Parameter change** | | | | | | |
| --- | --- | --- | --- | --- | --- | --- | --- |
|  | ρ | τ_2_ | k_off_ | W_1_ | | | T_2_ |
| Set 2: 81 populations | *1.1 | any | any | any | | | any |
|  | | | | | | | |
|  | K_2_ | τ_1_ | k_nc_ | | W_0_ | T_2_ | |
| Set 3: 81 populations | *1.1 | any | any | | any | any | |
|  | | | | | | | |
|  | K_1_ | ρ | Far1(0) | | W_1_ | T_2_ | |
| Set 4: 27 populations | - | *1.1 | any | | any | any | |
| Set 4: 27 populations | *10 | *1.1 | any | | any | any | |

*Discussion on the non-uniqueness of parameter sets (extracted from revised* ***Discussion****)*

The quantitative outputs of a multi-parameter model—such as the one presented here—that seeks to describe complex biological functions often lack the resolution necessary to identify each parameter within the parameter space uniquely. This limitation arises from a widely recognized intrinsic property of complex biological models known as sloppiness, which refers to the highly anisotropic geometry of the model’s parameter space (Grabowski *et al*, 2023). Specifically, model behavior tends to be highly sensitive to perturbations along a limited number of stiff parameter combinations, while remaining largely insensitive to variations along a multitude of sloppy directions.

Identifying stiff and sloppy directions within the parameter space—i.e., combinations of parameters whose variations produce substantial or negligible changes in model outputs—offers more meaningful insights into the model's qualitative dynamics than conventional analysis based on individual parameter variation (Grabowski *et al*, 2023). The prevalence of sloppy directions renders the task of accurately estimating all parameters from experimental data fundamentally ill-conditioned, particularly in the presence of measurement noise. This does not preclude the utility of such models in generating reliable simulations and predictive outcomes, even in the face of parameter uncertainty. Analogous to biological cells that maintain robust behavior despite stochastic fluctuations in individual molecular reactions, models can remain predictive within certain bounds of parameter variability (Gutenkunst *et al*, 2007; Apgar *et al*, 2010; Waterfall *et al*, 2006).

We conducted a sensitivity analysis to assess the robustness of selected population-level outputs—specifically, temporal dynamics and protein distributions—to individual parameter perturbations in the *GroCy-2* model. The resulting heatmap (Fig. 3D) reveals that most perturbations occur in sloppy directions, indicated by minimal output changes (gray regions). Similarly, reproducing wild-type temporal and growth phenotypes under varying glucose or ethanol conditions required adjusting only a small subset of parameters (Fig. 4C–I). Based on the sensitivity analysis (Fig. 3D), we also pinpointed key parameters whose variation accounts for the temporal and growth behaviors of mutant strains impaired in one of the model's three core functions: metabolism, growth, or cell cycle (Fig. 7-8; Supplementary Fig. S16). This identification is crucial for linking observed phenotypes to specific biological processes at a resolution consistent with the model’s structure.

Parameter sloppiness implies that various parameter combinations can produce similar phenotypes. As proof of concept, Supplementary Fig. S13 illustrates that different combinations of parameters may yield similar phenotypic outputs when parameters are allowed to co-vary in sloppy directions (see, e.g., the cloud of simulations with varying combinations of parameter values belonging to “Cluster Low,” which generate nearly superimposable protein distributions, Supplementary Fig. S14). Conversely, there are stiff directions within a reduced set of parameter variations that primarily drive changes in phenotypic properties: for instance, this occurs when increasing growth-related parameters *ρ* and *K_2_*, shifting the output populations towards the “Cluster High,” resulting in a dramatic increase in cell size compared to the “Cluster Low.”

(c) Are there other validation strategies beyond those reported, for assessing the model's biological plausibility?

As with all models, additional validations are possible, such as experimental tuning of parameters through genetic engineering and testing the resulting behavior; however, that would be outside the scope of this manuscript. We believe we provide ample validation that the model captures the biology of growth-rate-dependent cell-size regulation. Models are tools to generate hypotheses and insights, and we have explained better what these new insights are (see answers to points **1** and **4, 5**).

4 and 5. While the model can simulate different growth conditions and mutants, the manuscript does not adequately explore the underlying molecular mechanisms. The analysis of mutants, for example, is limited to alterations in a few catalytic parameters, and a more comprehensive approach to understanding the molecular and genetic underpinnings of the observed phenotypes would make a stronger contribution. The integration of molecular data, for instance, the parameters for Whi5 mutants, could also be a model for the analysis of other pathways.

While the hybrid model is presented as an advantage of iMeGroCy-2, it’s not clear what is the specific value in this approach and how it enhances the model's predictive power. The manuscript needs to better explain why it is necessary to replace coarse-grained functions with more detailed molecular sub-models and why the same results cannot be obtained with the simpler model.

The analysis of the role of fermentation in the modulation of cell size has been greatly expanded and discussed within the context of the basic *iMeGroCy-2* model's possibilities. As noted above in reference to the *whi5* mutants, the level of resolution for predictions relies on the model’s granularity. Therefore, the logic of using a molecular plug-in to provide predictions with a higher molecular resolution is explored in more detail for the *whi5* mutants and is considered in light of potential expansion, particularly regarding metabolism and its links to growth. Hopefully, the revised **Discussion**, whose relevant paragraphs are pasted above for reference (they are part of the green text in answer to point **2**), will guide the reader in explaining “why it is necessary to replace coarse-grained functions with more detailed molecular sub-models and why the same results cannot be obtained with the simpler model.”

**Reviewer 1-specific references**

Adler SO, Spiesser TW, Uschner F, Münzner U, Hahn J, Krantz M & Klipp E (2022) A yeast cell cycle model integrating stress, signaling, and physiology. *FEMS Yeast Res* 22: foac026

Adrover MÀ, Zi Z, Duch A, Schaber J, González-Novo A, Jimenez J, Nadal-Ribelles M, Clotet J, Klipp E & Posas F (2011) Time-dependent quantitative multicomponent control of the G₁-S network by the stress-activated protein kinase Hog1 upon osmostress. *Sci Signal* 4: ra63

Agmon E, Spangler RK, Skalnik CJ, Poole W, Peirce SM, Morrison JH & Covert MW (2022) Vivarium: an interface and engine for integrative multiscale modeling in computational biology. *Bioinformatics* 38: 1972–1979

Ahn-Horst TA, Mille LS, Sun G, Morrison JH & Covert MW (2022) An expanded whole-cell model of E. coli links cellular physiology with mechanisms of growth rate control. *NPJ Syst Biol Appl* 8: 30

Alberghina L, Mariani L, Martegani E & Vanoni M (1983) Analysis of protein distribution in budding yeast. *Biotechnol Bioeng* 25: 1295–1310

Alberghina L, Rossi RL, Querin L, Wanke V & Vanoni M (2004) A cell sizer network involving Cln3 and Far1 controls entrance into S phase in the mitotic cycle of budding yeast. *J Cell Biol* 167: 433–43

Altenburg T, Goldenbogen B, Uhlendorf J & Klipp E (2019) Osmolyte homeostasis controls single-cell growth rate and maximum cell size of Saccharomyces cerevisiae. *NPJ Syst Biol Appl* 5: 34

Amoussouvi A, Teufel L, Reis M, Seeger M, Schlichting JK, Schreiber G, Herrmann A & Klipp E (2018) Transcriptional timing and noise of yeast cell cycle regulators-a single cell and single molecule approach. *NPJ Syst Biol Appl* 4: 17

Apgar JF, Witmer DK, White FM & Tidor B (2010) Sloppy models, parameter uncertainty, and the role of experimental design. *Mol Biosyst* 6: 1890–1900

Barberis M, Klipp E, Vanoni M & Alberghina L (2007) Cell size at S phase initiation: an emergent property of the G1/S network. *PLoS Comput Biol* 3: e64

Basan M, Hui S, Okano H, Zhang Z, Shen Y, Williamson JR & Hwa T (2015) Overflow metabolism in Escherichia coli results from efficient proteome allocation. *Nature* 528: 99–104

Björkeroth J, Campbell K, Malina C, Yu R, Di Bartolomeo F & Nielsen J (2020) Proteome reallocation from amino acid biosynthesis to ribosomes enables yeast to grow faster in rich media. *Proc Natl Acad Sci U S A* 117: 21804–21812

Brümmer A, Salazar C, Zinzalla V, Alberghina L & Höfer T (2010) Mathematical modelling of DNA replication reveals a trade-off between coherence of origin activation and robustness against rereplication. *PLoS Comput Biol* 6: e1000783

Bunne C, Roohani Y, Rosen Y, Gupta A, Zhang X, Roed M, Alexandrov T, AlQuraishi M, Brennan P, Burkhardt DB, *et al* (2024) How to build the virtual cell with artificial intelligence: Priorities and opportunities. *Cell* 187: 7045–7063

Chen KC, Calzone L, Csikasz-Nagy A, Cross FR, Novak B & Tyson JJ (2004) Integrative analysis of cell cycle control in budding yeast. *Mol Biol Cell* 15: 3841–62

Chen Y, Li F & Nielsen J (2022) Genome-scale modeling of yeast metabolism: retrospectives and perspectives. *FEMS Yeast Research* 22: foac003

Chen Y & Nielsen J (2019) Energy metabolism controls phenotypes by protein efficiency and allocation. *Proc Natl Acad Sci U S A* 116: 17592–17597

Choi H & Covert MW (2023) Whole-cell modeling of E. coli confirms that in vitro tRNA aminoacylation measurements are insufficient to support cell growth and predicts a positive feedback mechanism regulating arginine biosynthesis. *Nucleic Acids Res* 51: 5911–5930

Deisboeck TS, Wang Z, Macklin P & Cristini V (2011) Multiscale cancer modeling. *Annu Rev Biomed Eng* 13: 127–55

Di Talia S, Skotheim JM, Bean JM, Siggia ED & Cross FR (2007) The effects of molecular noise and size control on variability in the budding yeast cell cycle. *Nature* 448: 947–51

Di Talia S, Wang H, Skotheim JM, Rosebrock AP, Futcher B & Cross FR (2009) Daughter-specific transcription factors regulate cell size control in budding yeast. *PLoS Biol* 7: e1000221

Dunayevich P, Baltanás R, Clemente JA, Couto A, Sapochnik D, Vasen G & Colman-Lerner A (2018) Heat-stress triggers MAPK crosstalk to turn on the hyperosmotic response pathway. *Sci Rep* 8: 15168

Eissing T, Kuepfer L, Becker C, Block M, Coboeken K, Gaub T, Goerlitz L, Jaeger J, Loosen R, Ludewig B, *et al* (2011) A computational systems biology software platform for multiscale modeling and simulation: integrating whole-body physiology, disease biology, and molecular reaction networks. *Front Physiol* 2: 4

Elsemman IE, Rodriguez Prado A, Grigaitis P, Garcia Albornoz M, Harman V, Holman SW, van Heerden J, Bruggeman FJ, Bisschops MMM, Sonnenschein N, *et al* (2022) Whole-cell modeling in yeast predicts compartment-specific proteome constraints that drive metabolic strategies. *Nat Commun* 13: 801

Flamholz A, Noor E, Bar-Even A, Liebermeister W & Milo R (2013) Glycolytic strategy as a tradeoff between energy yield and protein cost. *Proc Natl Acad Sci U S A* 110: 10039–44

Förster J, Famili I, Fu P, Palsson BØ & Nielsen J (2003) Genome-Scale Reconstruction of the Saccharomyces cerevisiae Metabolic Network. *Genome Res* 13: 244–253

Georgouli K, Yeom J-S, Blake RC & Navid A (2023) Multi-scale models of whole cells: progress and challenges. *Front Cell Dev Biol* 11

Gerber S, Fröhlich M, Lichtenberg-Fraté H, Shabala S, Shabala L & Klipp E (2016) A Thermodynamic Model of Monovalent Cation Homeostasis in the Yeast Saccharomyces cerevisiae. *PLoS Comput Biol* 12: e1004703

Goel A, Wortel MT, Molenaar D & Teusink B (2012) Metabolic shifts: A fitness perspective for microbial cell factories. *Biotechnology Letters* 34: 2147–2160

Grabowski F, Nałęcz-Jawecki P & Lipniacki T (2023) Predictive power of non-identifiable models. *Sci Rep* 13: 11143

Gutenkunst RN, Waterfall JJ, Casey FP, Brown KS, Myers CR & Sethna JP (2007) Universally Sloppy Parameter Sensitivities in Systems Biology Models. *PLOS Computational Biology* 3: e189

Hartwell LH & Unger MW (1977) Unequal division in Saccharomyces cerevisiae and its implications for the control of cell division. *J Cell Biol* 75: 422–35

Johnston GC, Ehrhardt CW, Lorincz A & Carter BL (1979) Regulation of cell size in the yeast Saccharomyces cerevisiae. *J Bacteriol* 137: 1–5

Jorgensen P, Nishikawa JL, Breitkreutz BJ & Tyers M (2002) Systematic identification of pathways that couple cell growth and division in yeast. *Science* 297: 395–400

Kahm M & Kschischo M (2016) Mathematical Modelling of Cation Transport and Regulation in Yeast. *Adv Exp Med Biol* 892: 291–305

Kaizu K & Takahashi K (2023) Technologies for whole-cell modeling: Genome-wide reconstruction of a cell in silico. *Dev Growth Differ* 65: 554–564

Karr JR, Sanghvi JC, Macklin DN, Gutschow MV, Jacobs JM, Bolival B, Assad-Garcia N, Glass JI & Covert MW (2012) A whole-cell computational model predicts phenotype from genotype. *Cell* 150: 389–401

Kerkhoven EJ (2022) Advances in constraint-based models: methods for improved predictive power based on resource allocation constraints. *Curr Opin Microbiol* 68: 102168

Klipp E, Nordlander B, Krüger R, Gennemark P & Hohmann S (2005) Integrative model of the response of yeast to osmotic shock. *Nat Biotechnol* 23: 975–982

Lao-Martil D, Schmitz JPJ, Teusink B & van Riel NAW (2023) Elucidating yeast glycolytic dynamics at steady state growth and glucose pulses through kinetic metabolic modeling. *Metab Eng* 77: 128–142

Lord PG & Wheals AE (1980) Asymmetrical division of Saccharomyces cerevisiae. *J Bacteriol* 142: 808–18

Lu H, Kerkhoven EJ & Nielsen J (2022) Multiscale models quantifying yeast physiology: towards a whole-cell model. *Trends Biotechnol* 40: 291–305

Lubitz T, Welkenhuysen N, Shashkova S, Bendrioua L, Hohmann S, Klipp E & Krantz M (2015) Network reconstruction and validation of the Snf1/AMPK pathway in baker’s yeast based on a comprehensive literature review. *NPJ Syst Biol Appl* 1: 15007

Macklin DN, Ahn-Horst TA, Choi H, Ruggero NA, Carrera J, Mason JC, Sun G, Agmon E, DeFelice MM, Maayan I, *et al* (2020) Simultaneous cross-evaluation of heterogeneous E. coli datasets via mechanistic simulation. *Science* 369: eaav3751

Metzl-Raz E, Kafri M, Yaakov G, Soifer I, Gurvich Y & Barkai N (2017) Principles of cellular resource allocation revealed by condition-dependent proteome profiling. *eLife* 6: e28034

Molenaar D, Van Berlo R, De Ridder D & Teusink B (2009) Shifts in growth strategies reflect tradeoffs in cellular economics. *Molecular Systems Biology* 5: 1–10

Mori M, Marinari E & De Martino A (2019) A yield-cost tradeoff governs Escherichia coli’ s decision between fermentation and respiration in carbon-limited growth. *NPJ Syst Biol Appl* 5: 16

Münzner U, Klipp E & Krantz M (2019) A comprehensive, mechanistically detailed, and executable model of the cell division cycle in Saccharomyces cerevisiae. *Nat Commun* 10: 1308

Murray LE, Veinot-Drebot LM, Hanic-Joyce PJ, Singers RA & Johnston GC (1987) Effect of ploidy on the critical size for cell proliferation of the yeast Saccharomyces cerevisiae. *Curr Genet* 11: 591–594

Nilsson A & Nielsen J (2016) Metabolic Trade-offs in Yeast are Caused by F1F0-ATP synthase. *Sci Rep* 6: 22264

O’Brien EJ, Lerman JA, Chang RL, Hyduke DR & Palsson B (2013) Genome-scale models of metabolism and gene expression extend and refine growth phenotype prediction. *Mol Syst Biol* 9: 693

Oftadeh O, Salvy P, Masid M, Curvat M, Miskovic L & Hatzimanikatis V (2021) A genome-scale metabolic model of Saccharomyces cerevisiae that integrates expression constraints and reaction thermodynamics. *Nat Commun* 12: 4790

Österberg L, Domenzain I, Münch J, Nielsen J, Hohmann S & Cvijovic M (2021) A novel yeast hybrid modeling framework integrating Boolean and enzyme-constrained networks enables exploration of the interplay between signaling and metabolism. *PLOS Computational Biology* 17: e1008891

Palumbo P, Vanoni M, Cusimano V, Busti S, Marano F, Manes C & Alberghina L (2016) Whi5 phosphorylation embedded in the G1/S network dynamically controls critical cell size and cell fate. *Nat Commun* 7: 11372

Palumbo P, Vanoni M, Papa F, Busti S, Wortel M, Teusink B & Alberghina L (2018) An integrated model quantitatively describing metabolism, growth and cell cycle in budding yeast. In Communications in Computer and Information Science  830

Paulo JA, O’Connell JD, Everley RA, O’Brien J, Gygi MA & Gygi SP (2016) Quantitative mass spectrometry-based multiplexing compares the abundance of 5000 S. cerevisiae proteins across 10 carbon sources. *J Proteomics* 148: 85–93

Pfeiffer T, Schuster S & Bonhoeffer S (2001) Cooperation and competition in the evolution of ATP-producing pathways. *Science* 292: 504–507

Pomeroy AE, Peña MI, Houser JR, Dixit G, Dohlman HG, Elston TC & Errede B (2021) A predictive model of gene expression reveals the role of network motifs in the mating response of yeast. *Sci Signal* 14: eabb5235

Porro D, Brambilla L & Alberghina L (2003) Glucose metabolism and cell size in continuous cultures of Saccharomyces cerevisiae. *FEMS Microbiol Lett* 229: 165–71

Porro D, Vai M, Vanoni M, Alberghina L & Hatzis C (2009) Analysis and modeling of growing budding yeast populations at the single cell level. *Cytometry Part A* 75A: 114–120

Qian L, Dong Z & Guo T (2025) Grow AI virtual cells: three data pillars and closed-loop learning. *Cell Res* 35: 319–321

Sánchez BJ, Zhang C, Nilsson A, Lahtvee P, Kerkhoven EJ & Nielsen J (2017) Improving the phenotype predictions of a yeast genome‐scale metabolic model by incorporating enzymatic constraints. *Molecular Systems Biology* 13: 935

Schaber J, Baltanas R, Bush A, Klipp E & Colman-Lerner A (2012) Modelling reveals novel roles of two parallel signalling pathways and homeostatic feedbacks in yeast. *Mol Syst Biol* 8: 622

Schnitzer B, Österberg L, Skopa I & Cvijovic M (2022) Multi-scale model suggests the trade-off between protein and ATP demand as a driver of metabolic changes during yeast replicative ageing. *PLoS Comput Biol* 18: e1010261

Schützhold V, Hahn J, Tummler K & Klipp E (2016) Computational Modeling of Lipid Metabolism in Yeast. *Front Mol Biosci* 3: 57

Seeger M, Flöttmann M & Klipp E (2023) A dynamical stochastic model of yeast translation across the cell cycle. *Heliyon* 9: e13101

Shen Y, Dinh HV, Cruz ER, Chen Z, Bartman CR, Xiao T, Call CM, Ryseck R-P, Pratas J, Weilandt D, *et al* (2024) Mitochondrial ATP generation is more proteome efficient than glycolysis. *Nat Chem Biol* 20: 1123–1132

Sherman F (2002) Getting started with yeast. In *Methods in Enzymology*, Guthrie C & Fink GR (eds) pp 3–41. Academic Press

Shi S & Li C (2025) Innovative Tools for Customized Yeast Cell Factory. *BioDesign Research*: 100010

Skalnik CJ, Cheah SY, Yang MY, Wolff MB, Spangler RK, Talman L, Morrison JH, Peirce SM, Agmon E & Covert MW (2023) Whole-cell modeling of E. coli colonies enables quantification of single-cell heterogeneity in antibiotic responses. *PLoS Comput Biol* 19: e1011232

Spiesser TW, Diener C, Barberis M & Klipp E (2010) What influences DNA replication rate in budding yeast? *PLoS One* 5: e10203

Stojanovski K, Ferrar T, Benisty H, Uschner F, Delgado J, Jimenez J, Solé C, de Nadal E, Klipp E, Posas F, *et al* (2017) Interaction Dynamics Determine Signaling and Output Pathway Responses. *Cell Rep* 19: 136–149

Sun G, Ahn-Horst TA & Covert MW (2021) The E. coli Whole-Cell Modeling Project. *EcoSal Plus* 9: eESP00012020

Sunchu B & Cabernard C (2020) Principles and mechanisms of asymmetric cell division. *Development* 147: dev167650

Szigeti B, Roth YD, Sekar JAP, Goldberg AP, Pochiraju SC & Karr JR (2018) A blueprint for human whole-cell modeling. *Curr Opin Syst Biol* 7: 8–15

Thornburg ZR, Bianchi DM, Brier TA, Gilbert BR, Earnest TM, Melo MCR, Safronova N, Sáenz JP, Cook AT, Wise KS, *et al* (2022) Fundamental behaviors emerge from simulations of a living minimal cell. *Cell* 185: 345-360.e28

Toscano E, Cimmino E, Boccia A, Sepe L & Paolella G (2025) Cell populations simulated in silico within SimulCell accurately reproduce the behaviour of experimental cell cultures. *npj Syst Biol Appl* 11: 1–17

Transtrum MK & Qiu P (2012) Optimal experiment selection for parameter estimation in biological differential equation models. *BMC Bioinformatics* 13: 181

Tummler K & Klipp E (2024) Data integration strategies for whole-cell modeling. *FEMS Yeast Research* 24: foae011

Van Hoek P, Van Dijken JP & Pronk JT (1998) Effect of specific growth rate on fermentative capacity of baker’s yeast. *Appl Environ Microbiol* 64: 4226–33

Walpole J, Papin JA & Peirce SM (2013) Multiscale computational models of complex biological systems. *Annu Rev Biomed Eng* 15: 137–54

Wang X (2025) Overflow metabolism originates from growth optimization and cell heterogeneity. *eLife* 13

Waterfall JJ, Casey FP, Gutenkunst RN, Brown KS, Myers CR, Brouwer PW, Elser V & Sethna JP (2006) Sloppy-model universality class and the Vandermonde matrix. *Phys Rev Lett* 97: 150601

Welkenhuysen N, Schnitzer B, Österberg L & Cvijovic M (2018) Robustness of Nutrient Signaling Is Maintained by Interconnectivity Between Signal Transduction Pathways. *Front Physiol* 9: 1964

Wortel MT, Bosdriesz E, Teusink B & Bruggeman FJ (2016) Evolutionary pressures on microbial metabolic strategies in the chemostat. *Sci Rep* 6: 29503

Xia J, Sánchez BJ, Chen Y, Campbell K, Kasvandik S & Nielsen J (2022) Proteome allocations change linearly with the specific growth rate of Saccharomyces cerevisiae under glucose limitation. *Nat Commun* 13: 2819

Ye C, Xu N, Gao C, Liu G, Xu J, Zhang W, Chen X, Nielsen J & Liu L (2020) Comprehensive understanding of Saccharomyces cerevisiae phenotypes with whole-cell model WM_S288C. *Biotechnol Bioeng* 117: 1562–1574

Zhang C, Sánchez BJ, Li F, Eiden CWQ, Scott WT, Liebal UW, Blank LM, Mengers HG, Anton M, Rangel AT, *et al* (2024) Yeast9: a consensus genome-scale metabolic model for S. cerevisiae curated by the community. *Mol Syst Biol* 20: 1134–1150

Zhang J, Schneider C, Ottmers L, Rodriguez R, Day A, Markwardt J & Schneider BL (2002) Genomic Scale Mutant Hunt Identifies Cell Size Homeostasis Genes in *S*. *cerevisiae*. *Current Biology* 12: 1992–2001

Zhong X, Zhao J & Sun L (2022) Integrative modeling of the cell. *Acta Biochim Biophys Sin (Shanghai)* 54: 1213–1221
